# Cation Dehydration by Surface-Grafted Phenyl Groups for Enhanced C_2+_ Production in Cu-Catalyzed Electrochemical CO_2_ Reduction

**DOI:** 10.1021/jacs.5c11313

**Published:** 2025-09-08

**Authors:** Miyeon Chang, Suhwan Yoo, Wenchao Ma, Hubert Girault, Yun Jeong Hwang, Xile Hu

**Affiliations:** a Laboratory of Inorganic Synthesis and Catalysis (LSCI), Institute of Chemical Sciences and Engineering, 27218 École Polytechnique Fédéralede Lausanne (EPFL), Lausanne 1015, Switzerland; b Department of Chemistry, College of Natural Science, 26725 Seoul National University (SNU), Seoul 08826, Republic of Korea; c Institute of Chemical Sciences and Engineering, École Polytechnique Fédéralede Lausanne (EPFL), Lausanne 1015, Switzerland

## Abstract

The challenge to produce multicarbon (C_2+_) products in high current densities in the electrochemical reduction of carbon dioxide (CO_2_RR) has motivated intense research. However, the ability of solvated cations to tune and activate water for C_2+_ production in the CO_2_RR has been overlooked. In this study, we report the incorporation of a covalently grown layer of functionalized phenyl groups on the Cu surface that leads to a 7-fold increase in ethylene production (to −530 mA cm^–2^) and a 6-fold increase in C_2+_ products (to −760 mA cm^–2^). Our mechanistic study, notably by in situ infrared, isotope effect, and electrochemical impedance spectroscopy, reveals that the surface grafting provokes the reduction of the hydration shell around alkali cations at the electrode–electrolyte interface. This reduction weakens the hydrogen bond network and thus enhances water dissociation to provide a proton for the CO_2_RR. Moreover, it increases cation density at the outer Helmholtz plane, resulting in a larger local electric field that further stabilizes intermediates en route to C_2+_ production. Our study brings to light the crucial role of cations in modulating the interfacial water structure, which has to be optimal for efficient C_2+_ production in CO_2_RR.

## Graphical abstract

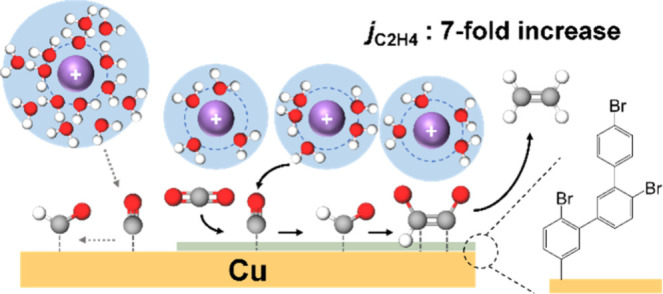

## Introduction

Electrochemical CO_2_ reduction reaction (CO_2_RR) has received significant attention for its potential in CO_2_ recycling.^1^ Copper (Cu)-based materials are particularly attractive as catalysts due to their largely unique ability to efficiently yield multicarbon (C_2+_) products with a notable Faradaic efficiency (FE).^2^ However, Cu-catalyzed CO_2_RR results in a mixture of products due to the presence of many competitive multielectron and multiproton transfer pathways. Despite recent advances in developing electrocatalysts for C_2+_ products, achieving a high current density for these products remains a challenge.

The complexity of CO_2_RR is compounded by the standard equilibrium potential for CO_2_RR being close to that of the hydrogen evolution reaction (HER), making H_2_ a common parasitic byproduct.^2,3^ Consequently, numerous studies have focused on designing catalysts that decrease H_2_O accessibility to the electrode, thereby reducing HER activity.^4–7^ More recently, researchers began to consider the role of H_2_O as a proton source for CO_2_RR.^8–13^ Given that H_2_O dissociation (often referred to as the Volmer step) or interfacial proton coupled electron transfer (PCET) with water as the proton donor is slow in alkaline media,^14,15^ optimizing the interfacial water configuration to facilitate proton delivery has been used to promote CO_2_RR. For instance, Ma et al. modified a Cu electrode with fluorine to enhance H_2_O activation and hydrogenation of *CO to ethylene,^12^ while Liu et al. optimized water dissociation by incorporating toluene assembly onto the Cu surface.^13^ These works demonstrate the potential to significantly improve the C_2+_ product activity by accelerating proton delivery.

Despite the aforementioned attempts to tune the interfacial water structure for the CO_2_RR, the role of alkali cations in regulating the interfacial water structure for the CO_2_RR has been overlooked. Although alkali cations are now well recognized as essential to CO_2_RR,^16–18^ their roles have so far been thought to be limited to generating a local electric field to stabilize CO_2_RR intermediates via dipole–field interactions^19,20^ or through the Coulombic interaction with negatively charged intermediates.^21–23^ Although alkali cations have been proposed to tune the kinetics of interfacial PCET to influence HER in alkaline medium,^14,24,25^ a potentially similar impact by alkali cations in Cu-catalyzed CO_2_RR has not been recognized prior to this study.

Herein, we report that an ultrathin (about 1 nm) layer of aromatic molecules covalently grown on the Cu surface can increase the current density of C_2_H_4_ by more than 7-fold. According to *operando* infrared (IR), isotope effects, and electrochemical impedance spectroscopy (EIS), the mechanism of the promotional effects is revealed as cation dehydration due to the organic layer at the electrode-solvent interface, leading to water activation and a stronger electric field, both of which enhance the activity and selectivity of CO_2_RR. Previously, Yang’s group reported a cation dehydration mechanism as the origin of enhanced CO production from CO_2_RR on Ag by phosphate ligands.^21^ However, their hypothesis was limited to cation-assisted intermediate stabilization without considering the further influence on the reorientation of the interfacial water structure.

## Results and Discussion

### Characterization of Functionalized Cu Nanoparticles

Commercially available CuO_*x*_ nanoparticles (NPs) were modified with functionalized phenyl groups using three aryl diazonium tetrafluoroborate salts in a nonelectrochemical grafting method (Scheme 1, see Methods, SI) to prepare Cu-PhR (R = Br, OMe, Cl_2_).^26,27^ Both unmodified and modified Cu catalysts exhibited a combination of Cu, Cu_2_O, and CuO phases, as shown by X-ray diffraction measurements, without noticeable differences among them (Figure S1). Scanning electron microscopy (SEM) and transmission electron microscopy (TEM) (Figures S2–S4) images revealed a spherical shape for unmodified Cu NPs and a nanoneedle morphology for modified Cu NPs. This morphological change originated from the treatment of Cu NPs in an alkaline medium during the grafting process,^28^ as shown in the SEM and TEM images of Cu–OH_soaked_, a sample prepared by treating bare Cu nanoparticles in 1 M NaOH, without the addition of diazonium salts (Methods, Figure S5). This morphological change alone should not influence the CO_2_RR activity (see below). X-ray photoelectron spectroscopy (XPS) Cu 2p spectra for both unmodified and modified Cu displayed a peak at 933 eV with no change in peak position (Figure S6), indicating that the grafting does not affect the oxidation state of Cu. The incorporation of functionalized phenyl groups for Cu-PhBr and Cu-PhCl_2_ was confirmed by peaks at ∼70 and ∼200 eV in the XPS spectra for Br 3d and Cl 2p (Figure S6), respectively. The presence of Br and Cl was further verified with electron dispersive X-ray spectroscopy (EDX) mapping using scanning TEM (STEM) (Figure S7). Diffuse reflectance infrared Fourier transform spectroscopy (DRIFTS) also confirmed the presence of molecular moieties on the Cu surface (Figure 1a). Signals at 1460 and 1490–1520 cm^–1^ were attributed to the aromatic C=C vibration.^29^ The C=C–C deformation peak from the aryl skeleton was observed at 1580–1600 cm^–1^,^30^ and aromatic C–H vibrations were observed at 820–850 cm^–1^.^29^

**Scheme 1:**
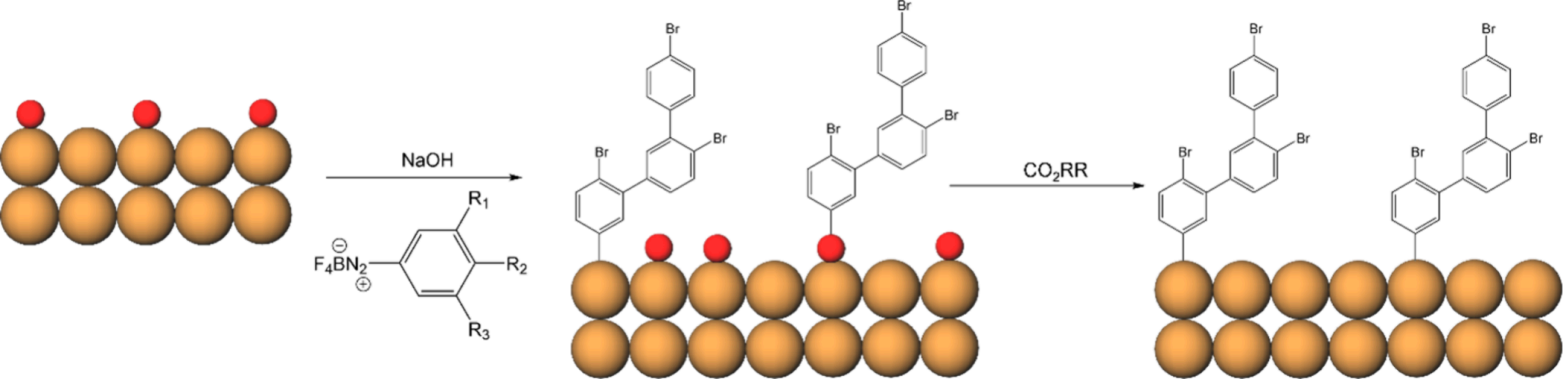
Preparation of Cu-PhR^a^

a Nonelectrochemical reaction to produce Cu-PhR using aryl diazonium salts. Only trimeric aryl groups are shown for simplicity. R_1_, R_3_ = H and R_2_ = Br for 4-bromobenzenediazonium tetrafluoroborate to prepare Cu-PhBr; R_1_, R_3_ = H and R_2_ = OMe for 4-methoxybenzenediazonium tetrafluoroborate to prepare Cu-PhOMe; R_1_, R_3_ = Cl and R_2_ = H for 3,5-dichlorophenyldiazonium tetrafluoroborate to prepare Cu-PhCl_2_. The grafting can take place on both copper and oxygen atoms, but once a negative potential is applied, copper oxide is reduced into copper. Only Cu-PhBr is represented. Brown, copper; red, oxygen.

**Figure 1:**
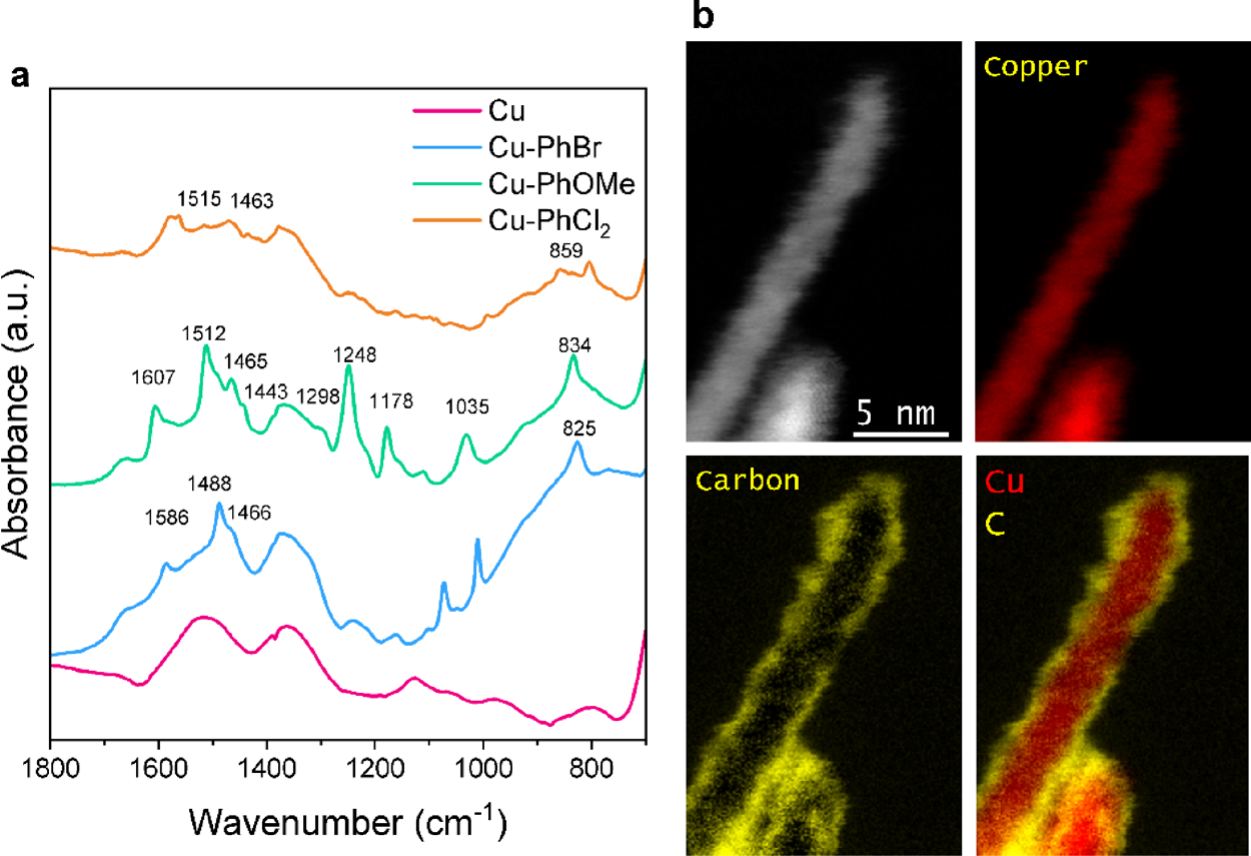
Characterization of the Cu-PhR catalysts. (a) DRIFTS spectra of Cu-PhR and bare Cu dispersed in KBr matrix. Diagnostic peaks are labeled and further discussed in the text. (b) ADF image (top, left) and the corresponding Cu (top, right), C (bottom, left), and merged (bottom, right) EELS elemental maps on Cu-PhBr using aberration corrected HR-STEM. The thickness of the carbon layer was determined to be about 1 nm.

In the DRIFTS spectrum of Cu-PhOMe, additional peaks from PhO–CH_3_ stretch (1035 cm^–1^), O–CH_3_ rocking (1178 cm^–1^), Ph–O (1248 cm^–1^ and 1298 cm^–1^) stretch, and OMe asymmetric stretch (1443 cm^–1^) were observed.^30,31^ The lack of a signal for the N–N triple bond from the diazonium salts at ∼2200 cm^–1^ indicated the absence of the starting reagent (Figure S8).^30^ Electron energy loss spectrometry (EELS) with high-resolution STEM (HR-STEM) confirmed the existence of a continuous carbon shell around the Cu nanoparticle for all Cu-PhR samples (Figure 1b and Figure S9). The thickness of this layer was around 1–2 nm for all samples, corresponding to a few units of phenyl groups.^32^

*In situ* surface-enhanced Raman spectroscopy (SERS) was also performed to examine the presence of Cu–C bond under reaction conditions (Figure S10). A signal at ∼420 cm^–1^ was observed for Cu-PhR, while it was absent for bare Cu between −0.1 and −0.5 V_RHE_. Given that Cu–C bonds appear between 300 and 500 cm^–1^ and the Cu–CO bond at 360 cm^–1^,^33^ we can assign this to the bond between Cu and the aryl group.

### Electrochemical CO_2_RR Performance

The electrochemical CO_2_RR performance of as-prepared Cu-PhR and bare Cu samples was evaluated in a gas-fed flow cell with 1 M KOH as the electrolyte (Figures S11 and S12). Linear swap voltammetry (LSV) under a N_2_ and CO_2_ atmosphere (Figure S13) revealed much higher cathodic current densities under CO_2_, consistent with CO_2_RR. FEs of CO_2_RR products were measured at fixed current densities (Figure S14). The major products included carbon monoxide, ethylene, formate, and ethanol, and the minor products included methane, *n*-propanol, and acetate (grouped together as *others*). At −50 mA cm^–2^, CO was the predominant product, which decreased with increasing negative current density, accompanied by an increase in C_2+_ products, particularly C_2_H_4_. All electrodes exhibited a similar trend in product selectivity.

The total and partial current densities for the CO_2_RR among all catalysts were compared (Figure 2a,b and Figure S15). Cu-PhOMe and Cu-PhCl_2_ exhibited 50–80 mV lower potentials, while Cu-PhBr exhibited 200 mV lower potentials, compared to those of bare Cu, at a given current density. Notably, Cu-PhBr achieved partial current densities of up to −530 mA cm^–2^ for C_2_H_4_ and −760 mA cm^–2^ for C_2+_ products at −0.65 V versus the reversible hydrogen electrode (V_RHE_), representing a 7.4-fold enhancement for C_2_H_4_ and 6.1-fold for C_2+_ over bare Cu. These current densities are among the highest for C_2_H_4_ and C_2+_ products for reported catalysts (Table S1). Cu-PhOMe and Cu-PhCl_2_ also increased *j*_C2H4_ and *j*_C2+_ by 3-fold compared with bare Cu. Notably, the surface phenyl groups also promote the formation of other products such as CO and H_2_, and to a lesser degree EtOH, in a similar trend (Figure S15). This result suggests that Cu-PhR enhances both the HER and the CO_2_RR intermediates. Postcatalytic characterization of Cu-PhBr after CO_2_RR by SEM and STEM suggests that the organic layer remains intact during CO_2_RR (Figure S16). Electrochemical surface area (ECSA) measurements showed that the surface areas of all electrodes are within a small range (Figures S17 and S18 and Table S2). The ECSA-normalized total current densities indicate that the activity enhancement arose mostly from an increase in the intrinsic activity of the catalyst, rather than an increase in ECSA. Cu-PhBr demonstrated stable performance under an industry-relevant current density of −200 mA cm^–2^ for 10 h, confirming the catalyst’s stability (Figure S19).

**Figure 2:**
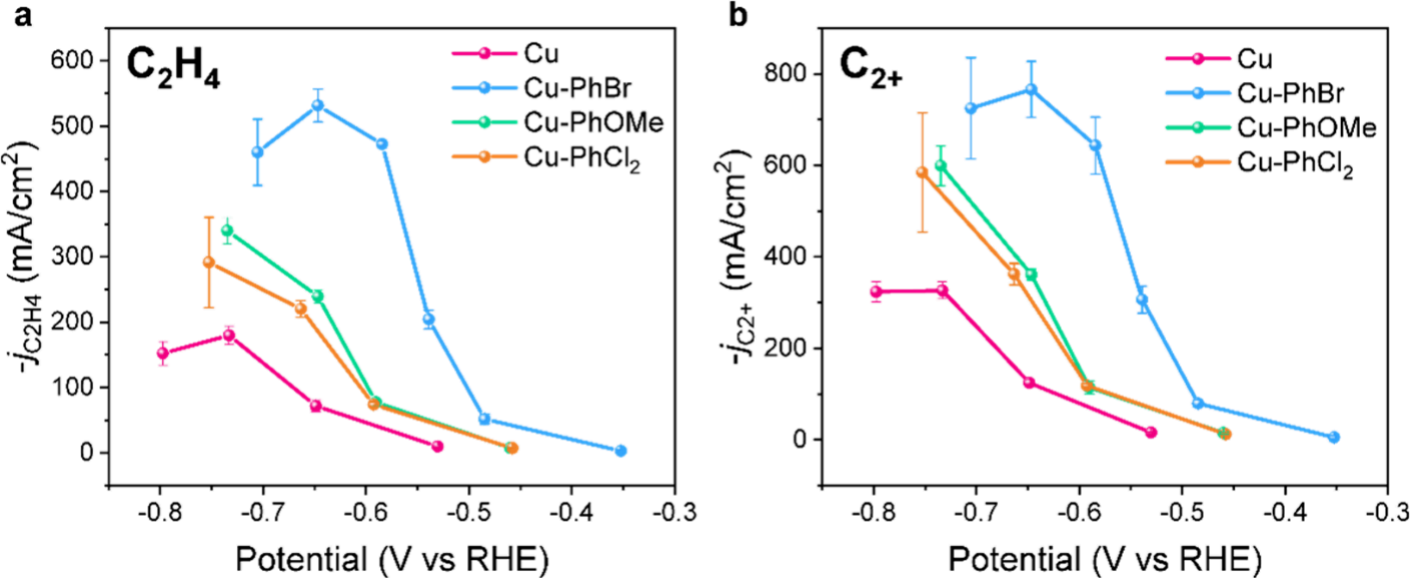
Electrocatalytic CO_2_RR performances. The partial current densities of (a) C_2_H_4_ and (b) C_2+_ products under different applied current densities for Cu-PhR in 1 M KOH using a flow cell setup. All the experiments were carried out three times, and the results show the mean value with an error bar of standard deviation.

To determine the impact of the amount of grafted groups, the loading of the organic layer was adjusted by varying the reagent amount during the synthesis of Cu-PhR (Figures S20–S22). Across all Cu-PhR samples, the low (L) loading exhibited slightly lower *j*_C2H4_ and *j*_C2+_ values than the medium (M) loading, whereas the high (H) loading showed a large deterioration. Nevertheless, the partial current densities for the H loading of Cu-PhBr were still higher compared to the optimal loading of Cu-PhOMe and Cu-PhCl_2_, suggesting that the thickness of the surface layer had at most a minor role in the activity or selectivity.

### Mechanistic Study of Cu-PhR

The in situ SERS showed signal for C–O stretch of adsorbed CO for all Cu and Cu-PhR samples under applied potentials, indicating that *CO adsorption strength is not the determining factor for the improved jC2H4­(Figure S23). The contact angle measurements (Figure S24 and Table S3) revealed hydrophobic surfaces for all electrodes, implying that hydrophobicity does not play a role in CO_2_RR, contrary to previous studies using organic modifiers to improve CO_2_RR on Cu.^4–6,34,35^ Cu–OH_soaked_ showed similar CO_2_RR performance to bare Cu, indicating that the morphology change due to the grafting medium was not the origin of the change in CO_2_RR activity or selectivity (Figure S25). Furthermore, this nanoneedle morphology is lost after reaction (Figure S16), confirming that morphology does not influence the CO_2_RR performance. To test whether local pH or suppressed (bi)­carbonate precipitate formation on the catalytic surface affects the performance, CO_2_RR was tested in a lower-pH electrolyte (0.5 M KHCO_3_, pH = 8.4) (Figure S26). Similar trends in *j*_C2H4_ and *j*_C2+_ to those in 1 M KOH indicate that neither local pH nor inhibition of (bi)­carbonate formation has an impact on the CO_2_RR performance. Furthermore, Cu-PhBr still outperforms bare Cu in the CO reduction reaction (CORR) conditions (Figure S27). Thus, neither CO_2_ activation nor enrichment of the catalytic surface with *CO intermediates can explain the improved CO_2_RR performance of Cu-PhBr.

The observed increase in the partial current densities for both the CO_2_RR products and H_2_ upon grafting suggested that surface hydrides, formed from water, play a role. To investigate the interfacial water structures at the Cu and modified Cu electrodes, O–H stretching modes (ν­(O–H)) were measured by *operando* attenuated total reflectance surface-enhanced infrared absorption spectroscopy (ATR-SEIRAS) during CO_2_RR (Figure 3). The ν­(O–H) peak, observed in the range of 3200–3400 cm^–1^, intensified as the potential was increased from −0.4 to −0.7 V_RHE_ due to the changes in water orientation (Figure S28).^36^

**Figure 3:**
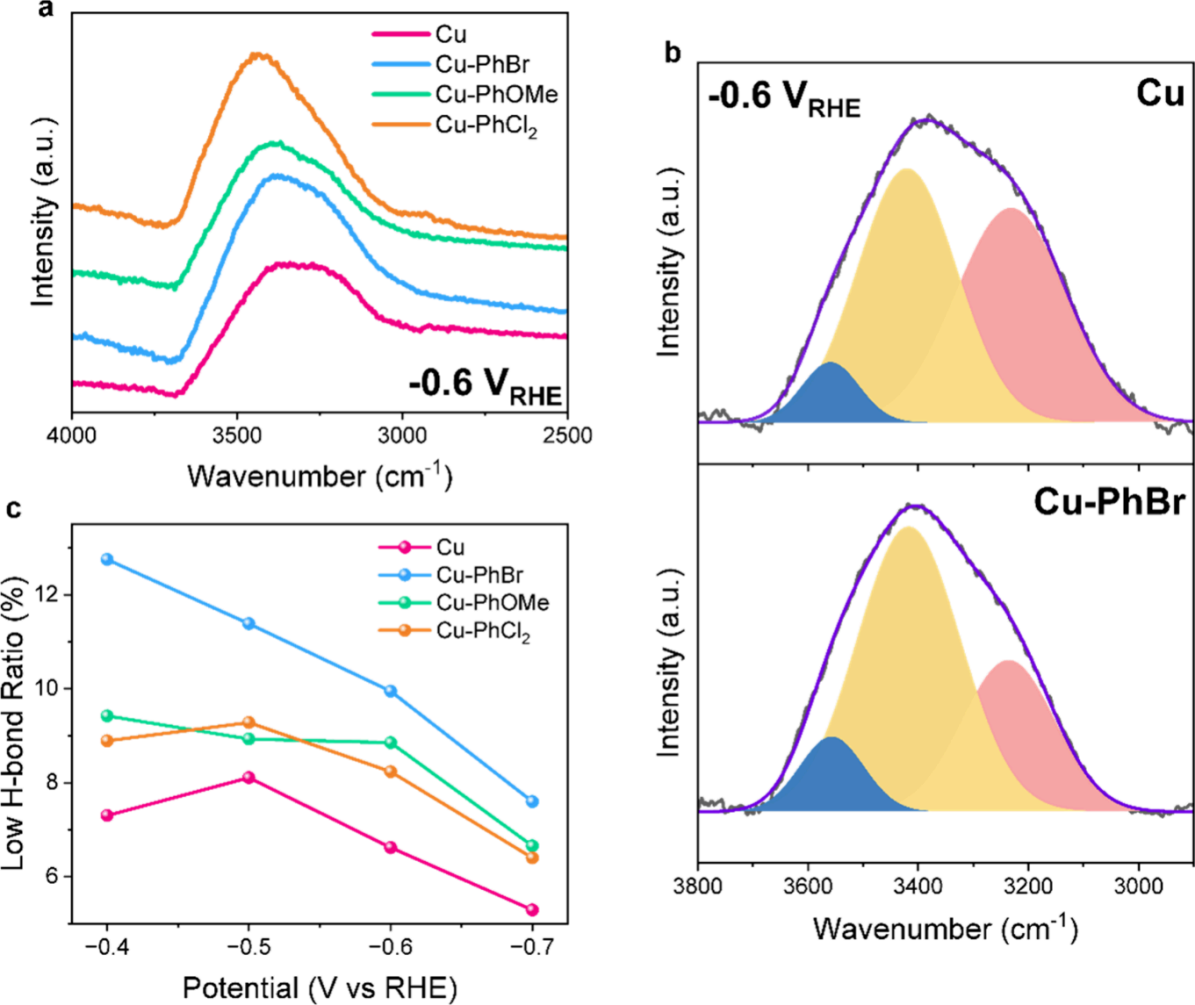
*Operando* ATR-SEIRAS spectra for ν­(O–H) of interfacial H_2_O in CO_2_-saturated 0.1 M KHCO_3_ (pH = 6.8). (a) Comparison of spectra between Cu and Cu-PhR at −0.6 V_RHE_. (b) Gaussian fit deconvolution from ν­(O–H) signal at −0.6 V_RHE_ for Cu (top) and Cu-PhBr (bottom) with free or low H-bonded H_2_O (blue), intermediate H-bonded H_2_O (yellow), and strong H-bonded H_2_O (red). (c) Water structure ratio for low H-bonded water for Cu and Cu-PhR under different applied potentials.

The ν­(O–H) bands vary by the applied potential as well as by the types of Cu catalysts. We compared the ν­(O–H) peak at −0.6 V_RHE_ (Figure 3a,b), a favorable potential for producing C_2_H_4_. The amplitude of ν­(O–H) peaks is similar for each sample (Figure 3a), which is consistent with the results from the contact angle measurements (Figure S24). The modified Cu electrodes at −0.6 V_RHE_ exhibited a relatively higher wavenumber than bare Cu (Figure 3a), which correlated to a weaker O–H strength, indicating that surface modification affected the strength of hydrogen bonding. The interfacial water structures were further analyzed according to the previous reports,^37,38^ which are deconvoluted by the degree of the hydrogen bond of the water structure: strong, intermediate, and low hydrogen-bonded water at 3200, 3400, and 3550 cm^–1^, respectively (Figure S29). Across various applied potentials, the spectra showed that Cu-PhBr exhibited a higher proportion of low hydrogen-bonded structures compared to the other electrodes (Figure 3c and Figure S29). Cu-PhOMe and Cu-PhCl_2_ also displayed more low-hydrogen-bonded water than bare Cu but less than Cu-PhBr. This trend aligns with the observed trend of *j*_C2H4_ and *j*_C2+_, where Cu-PhBr shows the highest activity and bare Cu shows the lowest activity (Figure 2). These results are consistent with previous reports suggesting that a weaker hydrogen bond network is more favorable for protonation in CO_2_RR.^13,39^ Previous studies also pointed out that a lower degree of H-bond facilitates water dissociation in alkaline media for HER.^38,40^ Taken together, the CO_2_RR activity and ATR-SEIRAS results suggest that modifying the Cu surface with phenyl groups can weaken the H-bond network at the interface, potentially contributing to facilitate this PCET step and leading to a high partial current density for the CO_2_RR.

Experiments were conducted at constant potentials to analyze the selectivity of CO_2_RR for further mechanistic study (Figures S30 and S31). The C_2_H_4_ selectivity (Figure 4b) is largely increased upon modification of bare Cu with phenyl groups, in the order of Cu-PhBr > Cu-PhOMe ≈ Cu-PhCl_2_ > Cu. At −0.58 V_RHE_, Cu-PhBr achieved a C_2_H_4_ FE of 42%, compared to 17% for bare Cu. In contrast, the FE of CO production was largely suppressed on modified Cu compared to bare Cu (Figure 4a), in the order of Cu-PhBr < Cu-PhOMe ≈ Cu-PhCl_2_ < Cu. Specifically, Cu-PhBr showed an FE of CO of 16% at −0.58 V_RHE_, compared to 38% for bare Cu. The ratio between *j*_C2H4_ and *j*_CO_ for Cu-PhBr was the highest (∼2.7), whereas the same ratio for bare Cu was the lowest (∼0.4) (Figure S32). These results demonstrate that organic groups on the Cu surface, particularly bromophenyl, stabilize intermediates such as *OCCO or *OCCHO to form C_2+_ products over direct CO desorption.

**Figure 4:**
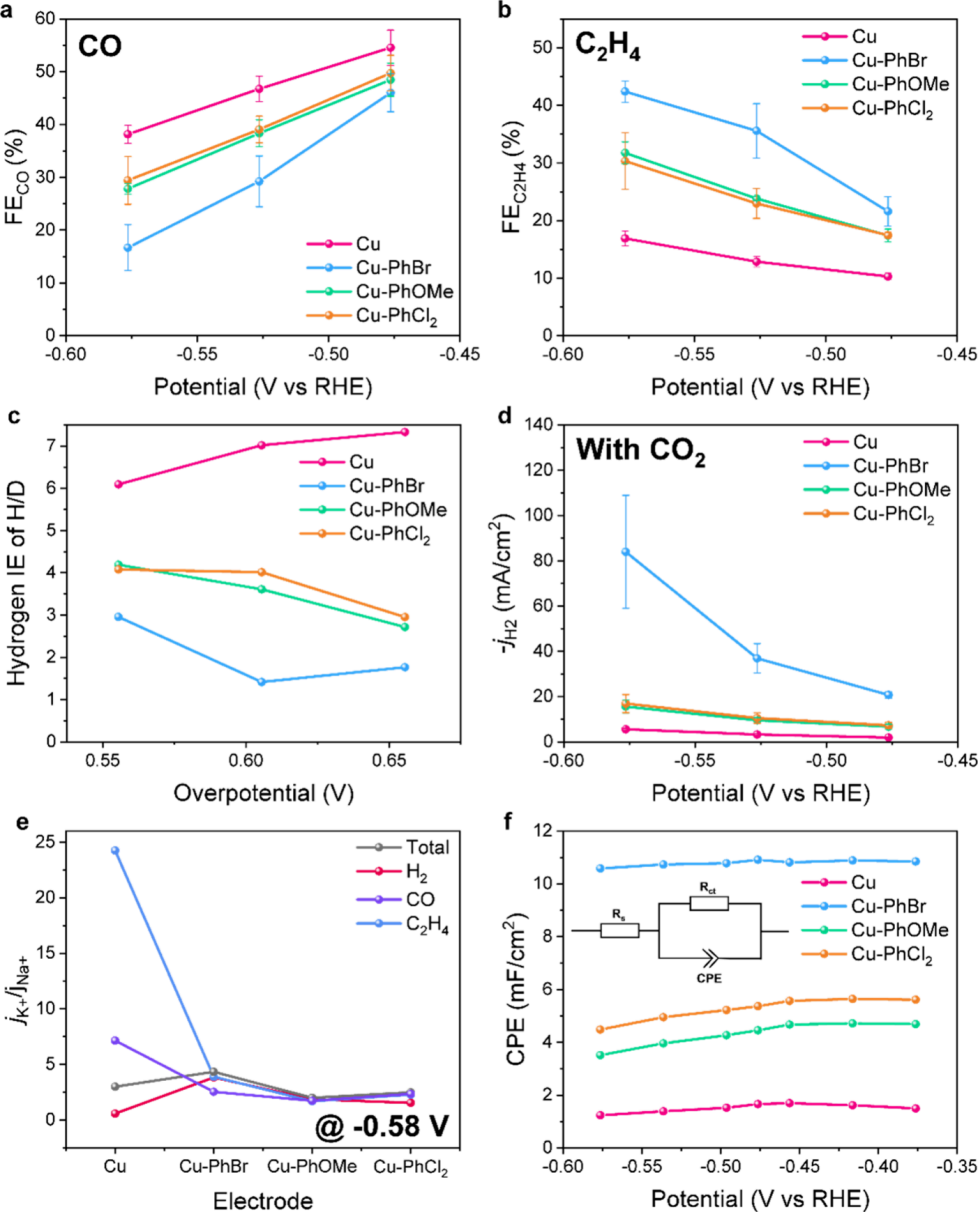
Mechanistic study of Cu and Cu-PhR. The FE values of (a) CO and (b) C_2_H_4_ under different applied potentials for Cu-PhR in 1 M KOH. (c) KIE of H/D for hydrogen with 1 M KOH in H_2_O and D_2_O. (d) HER activity under different applied potentials under CO_2_RR condition. (e) *j*_K+_ divided by *j*_Na+_ for total, H_2_, CO, and C_2_H_4_ current densities using bare Cu and Cu-PhR at −0.58 V_RHE_ by cation replacement from 1 M KOH to 1 M NaOH. (f) Pseudocapacitance measurements of Cu and Cu-PhR extracted from fitting of EIS measurements using the classical Randles circuit model at different DC potentials in 1 M KOH. Inset shows the model circuit for fitting using solution resistance (*R*_s_), charge transfer resistance (*R*_ct_), and constant-phase element (CPE).

To verify if the PCET with water as the proton donor is indeed accelerated, the kinetic isotope effect (KIE) was studied by replacing H_2_O with D_2_O in 1 M KOH electrolyte.^12,41^ KIE_hydrogen_ was higher than 1 for all electrodes, indicating a decelerated PCET process in D_2_O (Figure 4c and Figures S33 and S34). KIE_hydrogen_ was the highest for bare Cu at all potentials. The elevated KIE values on bare Cu suggest that the interfacial PCET with water is more sluggish than that with Cu-PhR. KIE_hydrogen_ has the order of Cu > Cu-PhOMe ≈ Cu-PhCl_2_ > Cu-PhBr. Consistent with this order, Cu-PhBr exhibited the highest HER activity in the absence and presence of CO_2_ (Figure 4d, Figures S15 and S35). These data indicate that the surface phenyl groups facilitate H_2_O dissociation to provide protons, which are needed for both HER and CO_2_RR. Note that the enhanced PCET would promote both HER and CO_2_RR. In agreement with this hypothesis, the selectivity of CO_2_RR, as expressed by *j*_CO2RR_ to *j*_H2_, is within a similar range for bare Cu and Cu modified by different phenyl groups (Table S4). The FE of H_2_ is similar for bare Cu and Cu-PhR, in contrast to the increase in the HER rate (Figure 4d and Figure S30). However, both the selectivity and activity toward ethylene are improved for Cu-PhBr (Figure 4b and Figure S30). Thus, instead of promoting overall CO_2_RR, grafting increased C_2+_, especially C_2_H_4_, production at the expense of CO formation.^42^ The KIE_ethylene_ is no more than 1 (Figures S36 and S37), and has the order of Cu > Cu-PhBr > Cu-PhOMe ≈ Cu-PhCl_2_. The interpretation of KIE_ethylene_ is not straightforward, as ethylene production involves multiple intermediates besides surface hydride, and the surface modification might alter the adsorption energies in different ways.

To inspect the influence of hydrated alkali cations M^+^(H_2_O)_*n*_ (M = Na, K) at the outer Helmholtz plane (OHP) on CO_2_RR, the electrolyte was changed from 1 M KOH to 1 M NaOH (Figures S38 and S39). It is recognized that alkali cations at the OHP induce a local electric field that stabilizes the dipoles of CO_2_RR intermediates.^16,20^ Since Na^+^ ions have a larger hydration shell than K^+^, there is a lower density of Na^+^ at the OHP than K^+^, leading to a decreased local electric field at the OHP.^20,43^ The ratios *R*_CO_ (7 at −0.58 V_RHE_) and *R*_C2H4_ (24 at −0.58 V_RHE_) (*R* = ratio of current densities in KOH and NaOH, *j*_K+_/*j*_Na+_) for bare Cu were large, consistent with the importance of the field for CO_2_RR.^16–18^ On the contrary, these ratios decrease greatly for all Cu-PhR electrodes (*R*_CO_ < 3; *R*_C2H4_ < 4; Figure 4e). These data indicate that surface modification decreased the difference in the fields generated by K^+^ and Na^+^ cations at the interface. The reduced difference in the field generated by K^+^ and Na^+^ cations suggests that the local electric fields generated by these cations became similar after modification. Thus, more choices of alkali electrolytes become available when using the modified Cu catalysts, which might bring benefits in either reducing the cost of the electrolytes or alleviating the problem of KHCO_3_ precipitation in the CO_2_RR.

Electrochemical impedance spectroscopy (EIS) was applied at catalytically relevant potentials (Figure S40) to probe the electrode interface structure. The charge transfer resistance (*R*_ct_) term from the fitted EIS data (Figure 4f inset, Figure S41) had the order of Cu > Cu-PhOMe ≈ Cu-PhCl_2_ > Cu-PhBr (Figure S42), in agreement with the catalytic activity (Figure 2a,b). The capacitive terms were extracted using a constant-phase element (CPE) in EIS (Figure 4f).^44^ The pseudocapacitance values of Cu-PhBr (∼10.7 mF cm^–2^), as well as Cu-PhOMe (∼4.3 mF cm^–2^) and Cu-PhCl_2_ (∼5.2 mF cm^–2^), were 7 and 3 times higher than that of bare Cu (∼1.5 mF cm^–2^). These capacitance values derived from EIS measurements show a different trend from *C*_dl_ obtained from CVs (Figure S18). This discrepancy arises from differences in measurement conditions, where EIS-capacitance is obtained under an applied potential and is influenced by reaction conditions, reactants, products, and intermediates. Even so, the surface area calculated from EIS-capacitance (Figure S43) confirms that the *j*_C2H4_ promotion does not originate from the change in EIS-capacitance. Given that the ECSA values were similar for all the electrodes (Figure S18), the increase in pseudocapacitance for Cu-PhR suggests enhanced adsorption of charges, possibly alkali cations, at the catalytic surface. The potential of zero charge (pzc) was further probed by the potential at which the CPE reaches its minimum (Figure S44).^39^ The more positive pzc of Cu-PhBr (−0.78 V versus standard hydrogen electrode, V_SHE_) than bare Cu (−0.87 V_SHE_) indicates enhanced cation density at the double layer of the former, suggesting that the increase in the pseudocapacitance upon grafting might indeed be due to more absorbed cations. Our hypothesis agrees with the work of the group of Yang, who also observed an increase in pseudocapacitance after modifying Cu with an organic/metal interlayer, and attributed it to the dehydration of alkali cations.^21^ Their K X-ray Absorption Near Edge Structure data and molecular dynamics results supported this assignment.

### Discussion

Based on the above data, we propose that the largely enhanced production of C_2+_ products on phenyl-modified Cu catalysts can be attributed to the reduced hydration around the interfacial cations (Figure 5). Without the grafted groups, interfacial water engages in stronger H-bonds with other water molecules (Figure 3), slowing down the PCET step with water, hence limiting HER and CO_2_RR. With the surface modification, the reduced hydration increases the cation–water interaction, leading to more weakly H-bonded H_2_O, activating the water. This enhanced proton availability accelerates both the HER and the CO_2_RR (Figure 4c,d). We note that Lee et al. had attributed a surface 4-bromothiophenyl group as kosmotropic.^45^ Yet their system exhibited worse HER activity, also in contrast to our system. The difference might be attributed to different electrolyte environments, but both studies indicate that better water dissociation leads to better HER activity.

**Figure 5:**
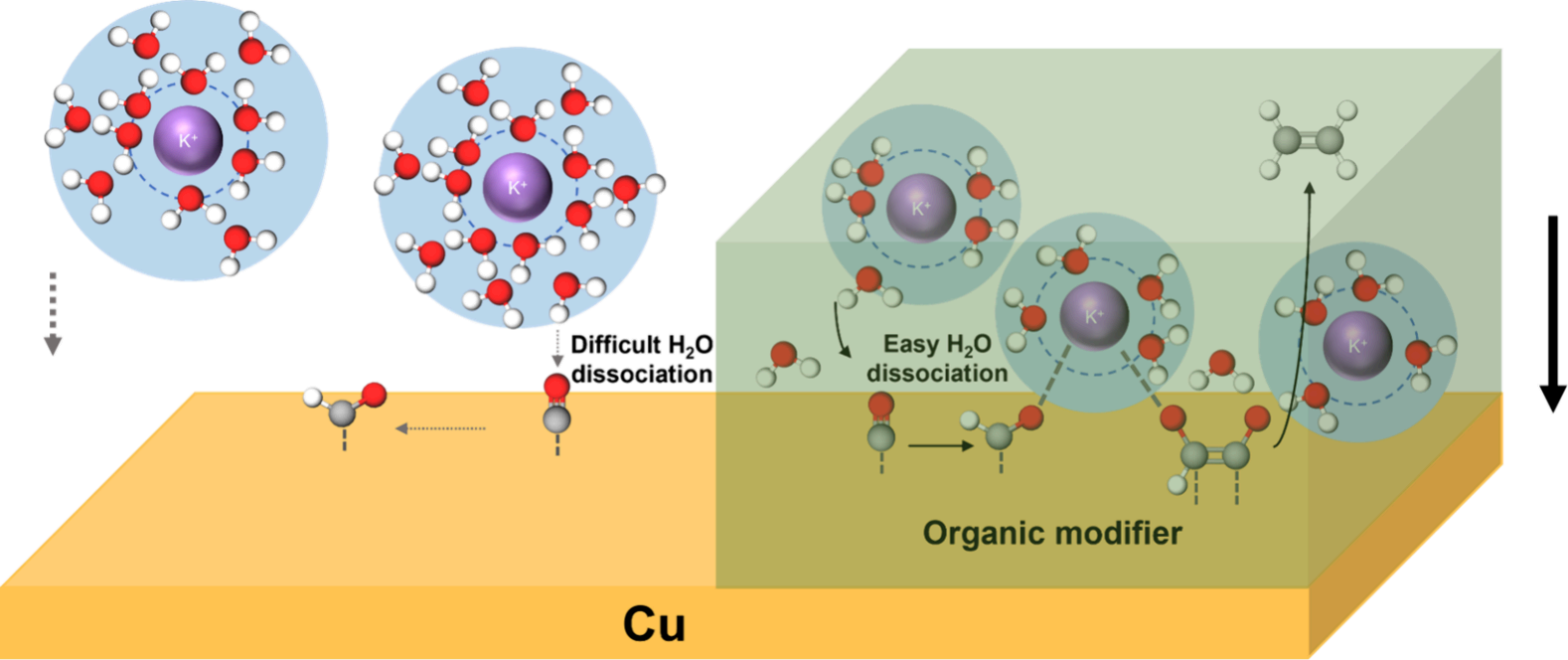
Schematic diagram of the proposed mechanism of CO_2_RR to C_2_H_4_ for unmodified (left) and modified Cu (right). Purple, potassium; gray, carbon; red, oxygen; white, hydrogen.

Our data show that Cu-PhR electrodes are less sensitive to the decrease in the density of hydrated cations at the OHP (Figure 4e), and they have increased pseudocapacitance compared to bare Cu (Figure 4f). These data point to a second factor that contributes to the higher CO_2_RR activity of the modified electrodes. As the cations are less hydrated, they can better approach the Cu electrodes, resulting in higher densities at the OHP and generating a larger local electric field. The enhanced field–dipole interactions stabilize the CO_2_RR intermediates. It has been previously reported that critical intermediates to C_2+_ products like *OCCO or *OCCHO^46,47^ possess a large dipole moment. Thus, these intermediates might be comparatively more stabilized than other intermediates in an enhanced field,^20,43,48,49^ leading to higher C_2+_ production. The formation and high FE of C_2+_ support that, despite the presence of a modified layer, cations can still approach the double layer of Cu-PhR to stabilize intermediates with a large dipole moment, so the aryl layer does not inhibit cation stabilization. Moreover, electrostatic interactions between negatively charged intermediates and less hydrated cations might provide an additional stabilization of the intermediates.^17^

There are several previous studies reporting facilitated water dissociation as the origin of enhanced ethylene formation from the CO_2_RR, but cation dehydration was not considered as a mechanism. For instance, Liang et al. identified enhanced water adsorption and dissociation as the cause of the increased C_2_H_4_ from CORR, but the role of cations in water dissociation was not discussed.^50^ Similarly, Liu et al. focused on the optimal water approach to the catalytic surface for proton donation rather than the impact of interfacial cations.^13^ Cation-bound water was also overlooked in Wang et al.’s work, where only the hydrogen bond structure of interfacial water was considered for promoted C_2+_ production.^51^ Ni et al. showed that adding organic solvents to aqueous electrolyte modifies the hydration sphere of cations to activate interfacial water and accelerate *CO_2_ protonation.^52^ However, their study was restricted to a CO-producing catalyst; therefore, the role of hydrated cations in accelerated water dissociation for ethylene production was not investigated.

Modifying the Cu surface with different phenyl groups gives a different level of improvement in the CO_2_RR. As the order of activity correlates with that of KIE_hydrogen_ and pseudocapacitance, we think the activity difference reflects the different degree of cation dehydration. The thickness of the surface layer does not have a major role (see above); thus, we focused on the chemical nature of the organic modifier. To probe the origins of the different activity of Cu-PhR, we further studied Cu-PhCl and Cu-PhF. Together with PhBr, they constitute the same class of mono- and para-substituted halo benzene molecules (unlike PhCl_2_). In this series, Cu-PhBr exhibited the highest *j*_C2H4_ and *j*_C2+_, while Cu-PhF showed the lowest activity (Figures S45a,b and S46). We notice that this trend aligns with the order of the chaotropic property of halobenzenes: PhBr > PhCl > PhF.^53,54^ Thus, we propose that the observed differences in catalytic performance might arise, at least in part, from variations in the chaotropic activity of the organic modifiers.

We further measured the activity of Cu-PhNEt_2_. Both Cu-PhNEt_2_ and Cu-PhOMe contain a polar functional group capable of forming H-bonds. The Kamlet–Taft β values, which describe a solvent’s ability to serve as a hydrogen bond acceptor, for *N*,*N*-dimethylaniline (0.43) and anisole (0.32) are higher than that for bromobenzene (0.06).^55,56^ Cu-PhNEt_2_ and Cu-PhOMe have similar performance, which is inferior to Cu-PhBr but superior to bare Cu (Figures S45c,d and S46). We hypothesize that because NEt_2_ and OMe groups can form hydrogen bonds with water, these groups are less effective than PhBr in weakening the hydrogen bonding network, which explains the performance trend.

Modification of the Cu catalyst with aromatic groups for the CO_2_RR was previously reported, but the influence of the molecular layers on solvated cations and the corresponding impact on the interfacial water structure had not been studied until now. For instance, Watkins et al. showed improved CO_2_RR performance with an electrodeposited polyaromatic layer on Cu film, but the authors involved only increased hydrophobicity and decreased water accessibility as the origin of the improvement.^29^ Wu et al. reported enhanced CO_2_RR by a functionalized phenyl group, but they invoked more optimal *CO absorption energy as the origin without a molecular interpretation.^57^ The Yang group had proposed cation dehydration as the origin of enhanced CO production from CO_2_RR on Ag by phosphate ligands.^21^ However, their hypothesis considered only the cation-intermediate stabilization but not the influence of dehydration in the interfacial water structure as we describe here. The group of Wang used ab initio molecular dynamics to study the mechanism of the CO–CO coupling at Cu. They found that a high coverage of CO leads to dehydration of cations due to the hydrophobicity of CO.^58^ Their results supported our hypothesis that organic modifiers led to cation dehydration at the interface. Nevertheless, Wang et al.’s work focused on the stabilization of the dimerized intermediate by cation dehydration but did not cover the modification of the interfacial water structure due to cation dehydration.^58^

## Conclusions

In summary, we demonstrate a greatly enhanced CO_2_RR performance, especially for ethylene production, by modifying Cu with an ultrathin layer of functionalized phenyl groups. The modifications increase the activity toward C_2+_ products, achieving up to −530 mA cm^–2^ for C_2_H_4_ (7-fold increase) and up to −760 mA cm^–2^ for C_2+_ products (6-fold increase). We reveal that the improvement is due to dehydration of interfacial cations. This dehydration facilitates the interfacial PCET step and proton availability, which is essential for both HER and CO_2_RR. At the same time, dehydration increases the cation density at the electrode, strengthening the local electric field. The increased field–dipole interactions stabilize CO_2_RR intermediates and promote, particularly, ethylene formation. Our work demonstrates a simple approach to modulate the cation–water interaction for CO_2_RR. The insights might be applied to develop further systems for CO_2_RR and other electrochemical reactions.

## Supplementary Material



## Data Availability

The raw data that support the findings of this publication are openly available on Zenodo at https://doi.org/10.5281/zenodo.17061511.

## Notes

Materials, detailed synthetic methods, characterization methods, FE calculation, XRD, SEM, TEM, STEM, elemental mapping, EELS mapping, XPS, FTIR, LSV, FE, and partial current densities for each CO_2_RR product, H_2_, total current densities, SEIRA spectra, KIE measurement, Niquist plot from EIS, and PZC (PDF)
