# Supplementary material for: Cation Dehydration by Surface-Grafted Phenyl Groups for Enhanced C2+ Production in Cu-Catalyzed Electrochemical CO2 Reduction
Source: J Am Chem Soc. 2025 Sep 8;147(37):34001–10. doi: 10.1021/jacs.5c11313 (PMC12447509; doi:10.1021/jacs.5c11313)
Supplement: Supplementary file 1 [file ja5c11313_si_001.pdf]

Supporting Information

Cation Dehydration by Surface-Grafted Phenyl Groups for Enhanced C_{2+} Production in Cu-catalyzed Electrochemical CO_2 Reduction

Miyeon Chang,[a] *Suhwan Yoo,*[b] *Wenchao Ma,*[a] *Hubert Girault,*[c] *Yun Jeong Hwang,*[b] *Xile Hu* [a,*]

[a] Laboratory of Inorganic Synthesis and Catalysis, Institute of Chemical Sciences and Engineering, Ecole Polytechnique Fédérale Lausanne (EPFL), 1015 Lausanne, Switzerland.

[b] Department of Chemistry, College of Natural Science, Seoul National University (SNU), Seoul, 08826 Republic of Korea.

[c] Institute of Chemical Sciences and Engineering, Ecole Polytechnique Fédérale Lausanne (EPFL), 1015 Lausanne, Switzerland.

METHODS

Pretreatment of Cu nanoparticles. The unmodified Cu nanoparticles were prepared by the pyrolysis of commercial 35 nm Cu nanoparticles (NANOGRAFI Co. Ltd.) in the air at 250 °C for 4 h with a heating rate of 10 °C min^{-1}.

Functionalization of Cu nanoparticles. The as-prepared Cu nanoparticles were dispersed in 5 mL of 2 M NaOH solution with sonication for 30 min. The 5 mL of 0.08 M solution of either 4-bromobenzenediazonium tetrafluoroborate (Thermo Scientific Alfa Aesar), 4-methoxybenzenediazonium tetrafluoroborate (Combi-Blocks) or 3,5-dichlorobenzenediazonium tetrafluoroborate (Toronto) was added drop by drop to the Cu nanoparticles solution. 4-chlorobenzenediazonium tetrafluoroborate and 4-fluorobenzendiazonium tetrafluoroborate were synthesized according to previous literature.[1] The mixture was left under agitation for 20 h, followed by washing with water for 5 times, acetonitrile for 2 times and EtOH for 3 times. The solids were then dried in air at 60 °C for overnight. The concentration of diazonium tetrafluoroborate salt was changed from 0.08 M (M for medium) to 0.04 M (L for low) and 0.16 M (H for high) to examine the loading effect. $Cu\text{-}OH_{soaked}$ sample was prepared following the same protocol as Cu-PhR, but without the addition of diazonium salts. It was subsequently washed using the identical procedure as Cu-PhR.

Material characterization. X-ray diffraction (XRD) patterns were obtained from Aeris diffractometer (Malvern Panalytical). A monochromatic Cu Kα radiation (1.54 Å) with Cu Kβ subtraction by Ni filter and PIXcel1D-Medipix3 detector were used. Catalysts in powder form were directly measured. X-ray photoelectron spectroscopy (XPS) analysis was executed with an AXIS Supr XPS system (Kratos Analytical). Al Kα source of 1486.7 eV was used to generate monochromatic X-Rays. Scanning electron microscopy (SEM) images were acquired on a Zeiss Merlin microscope at 3 kV. Transmission electron microscopy (TEM) were obtained on a Talos F200S microscope at 200 kV. High resolution scanning TEM (HR-STEM) images and electron energy loss spectra (EELS) were acquired on an aberration-corrected FEI Titan Themis TEM at 300 kV high tension.

Electrode preparation. The cathode catalyst ink was prepared by adding 12 mg of catalyst into a solution of 3 mL of EtOH and 25 µL of Nafion perfluorinated resin solution 1100 W (Sigma-Aldrich). 1100 W refers to 1100 equivalent weight (EW), grams of dry Nafion per mole of sulfonic acid groups.[2] The mixture then underwent 1 h of ultrasonication to obtain a homogeneously dispersed solution. The as-prepared ink was air-brushed onto vertically placed 3 cm x 3 cm AvCarb GDS3250 carbon paper on a heating plate at 70 °C. Finally, the electrode was cut into four pieces to give a 1.5 cm x 1.5 cm GDE.

Electrochemical measurements. A Gamry Reference 3000 electrochemical instrument was used to measure electrochemical activity at ambient temperature and pressure. Potential was first measured with

Ag/AgCl reference electrode (saturated KCl), which was then converted into potential versus reversible hydrogen electrode (RHE) with an automatic iR compensation of 85% using Equation 1. All potentials were measured against the reversible hydrogen electrode (RHE) unless indicated otherwise.

$$\mathrm{E_{RHE}} = \mathrm{E_{Ag/AgCl}} + 0.1976\ \mathrm{V} + 0.592 \times\ \mathrm{pH} + i\mathrm{R} \quad (1)$$

The measurement was carried out in a three-electrode custom-made gas diffusion electrode (GDE) flow cell with 1 cm x 1 cm windows. For sealing between each GDE and electrolyte chamber, silicon gasket with 1.5 cm x 1.5 cm window was used. Its cathodic and anodic chambers were filled with each 20 mL of 1 M potassium hydroxide (KOH). The counter electrode was NiFe/Ni foam synthesized according to a previous report.[3] FAA-3-PK-75 (Fumasep) anion exchange membrane was used to separate the two chambers. Chronopotentiometry measurements were completed at −50, −200, −500 and −1000 mA cm^{-2} for 600 s. For Cu-PhBr, additional measurements at –1250 and −1500 mA cm^{-2} were performed. Chronoamperometry measurements were performed at -0.48 V_{RHE}, –0.53 V_{RHE} and –0.58 V_{RHE} for 600 s. Three independent electrochemical measurements were performed for catalyst to give an average and a standard deviation for an error bar. The gas inlet on the cathode side was connected to CO_2 flow. To quantify gas products, the outlet was connected to a gas chromatography (GC) (SRI 8610C) equipped with a thermal conductivity detector (TCD) and a flame ionization detector (FID). The product gas was injected into GC after 450 s of CO_2RR. Then, using *Equation 2* where A is the area in the chromatogram, f the flow rate, N_{Av} Avogadro-s constant, n_{e-} the number of electrons, e the elementary charge, k the calibration constant for each product, I_{tot} the total current and V_{mol} the molar volume, we obtained FE of gas products.

$$FE = k \times A_{\mathrm{product}} \frac{f\ N_{Av}\ n_{e^-}\ e}{I_{tot}\ V_{mol}} \quad (2)$$

The liquid products were measured by 1H NMR with 5 mM dimethyl sulfoxide (DMSO) solution as the standard. After the end of each measurement, 400 μL of electrolyte was mixed with 50 μL deuterium oxide and 40 μL standard solution. Then, FE was calculated using Equation 3 with k the number of mol corresponding to a unit area, C the total charge, F the Faraday constant, n_H the number of hydrogens corresponding to a given NMR peak, V_{tot} the total volume of electrolyte during electrolysis and V_{elec} the volume of electrolyte used for NMR.

$$FE = k \times A_{\mathrm{product}} \frac{V_{tot}\ n_{e^-}}{n_H\ V_{elec}\ F\ C} \quad (3)$$

Solvent kinetic isotope effect (KIE) was measured by performing CO_2RR in 1 M KOH electrolyte. Overpotential (η) was calculated by taking into account the different pK_w values between H_2O (14.00) and D_2O (14.87). KIE of a given product was calculated by taking the ratio between the partial current density in H_2O and D_2O at the same overpotential (Equation 4). η_{H2O} and η_{D2O} can be calculated according to

Equation 5 and *Equation 6*. Note that the thermodynamic potential of CO_2RR to C_2H_4 is 0.07909 V_{RHE} and that of CO_2RR to C_2D_4 is 0.109 V_{RHE}.[4,5]

$$KIE_{\text{product}} = \frac{j_{\text{H2O}}}{j_{\text{D2O}}} \tag{3}$$

$$\eta_{\text{H2O}} = E_{\text{Ag/AgCl}} + 0.1976\ \text{V} + 0.0592\ \text{V} \times \ \text{pH} - 0.07909\ \text{V} \tag{4}$$

$$\eta_{\text{D2O}} = E_{\text{Ag/AgCl}} + 0.1976\ \text{V} + 0.0592\ \text{V} \times (\text{pH} + 0.87) - 0.109\ \text{V} \tag{5}$$

***Operando* ATR-SEIRAS.** A thin Au film was deposited on the Si crystal (20 mm in diameter, VeeMAX) using the previously reported method.[6] Catalysts on Au-deposited Si crystal (Catalyst/Au–Si) were prepared by spray-coating. Catalyst ink was prepared by sonicating for 15 min with a composition of 2 mg of the catalyst, 20 µL of Nafion solution, and 1 mL of ethanol. Catalyst/Au–Si was assembled into a home-made cell, and was transferred to the Fourier transform infrared spectroscopy (FT-IR, VERTEX 80v, Bruker) equipped with a mercury cadmium telluride (MCT) detector and ATR accessory (VeeMAX III, PIKE Technologies). A Pt wire and Ag/AgCl reference (saturated 3 M NaCl) were used for the counter and reference electrodes, respectively. CO_2-saturated 0.1 M $KHCO_3$ (pH = 6.8) was used as electrolyte. Due to carbonation of electrolyte and excessive bubble formation, hindering acquisition of spectra, 0.1 M $KHCO_3$ was used instead of 1 M KOH. All measurements were performed with a 4 cm^{-1} spectral resolution and 16 scans. The reference spectrum was obtained at OCP, and all spectra were represented in absorbance, −log(R/R0). The surface oxide was removed by conducting 4 times a linear sweep voltammetry (LSV) at 100 mV/s between 0 V_{RHE} and -0.6 V_{RHE}.

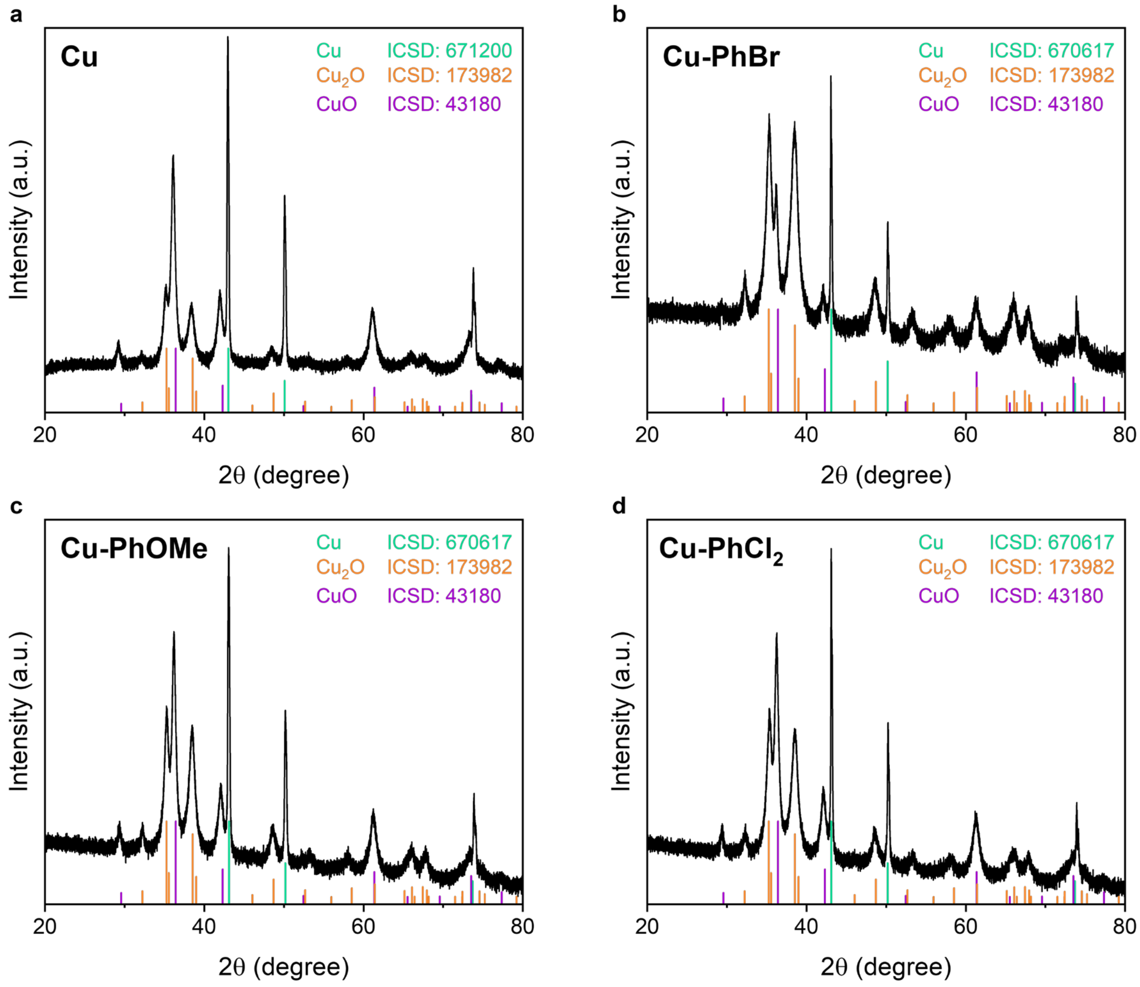

Figure S1. XRD patterns for Cu and Cu-PhR. a) Cu, b) Cu-PhBr, c) Cu-PhOMe and d) Cu-$PhCl_2$.

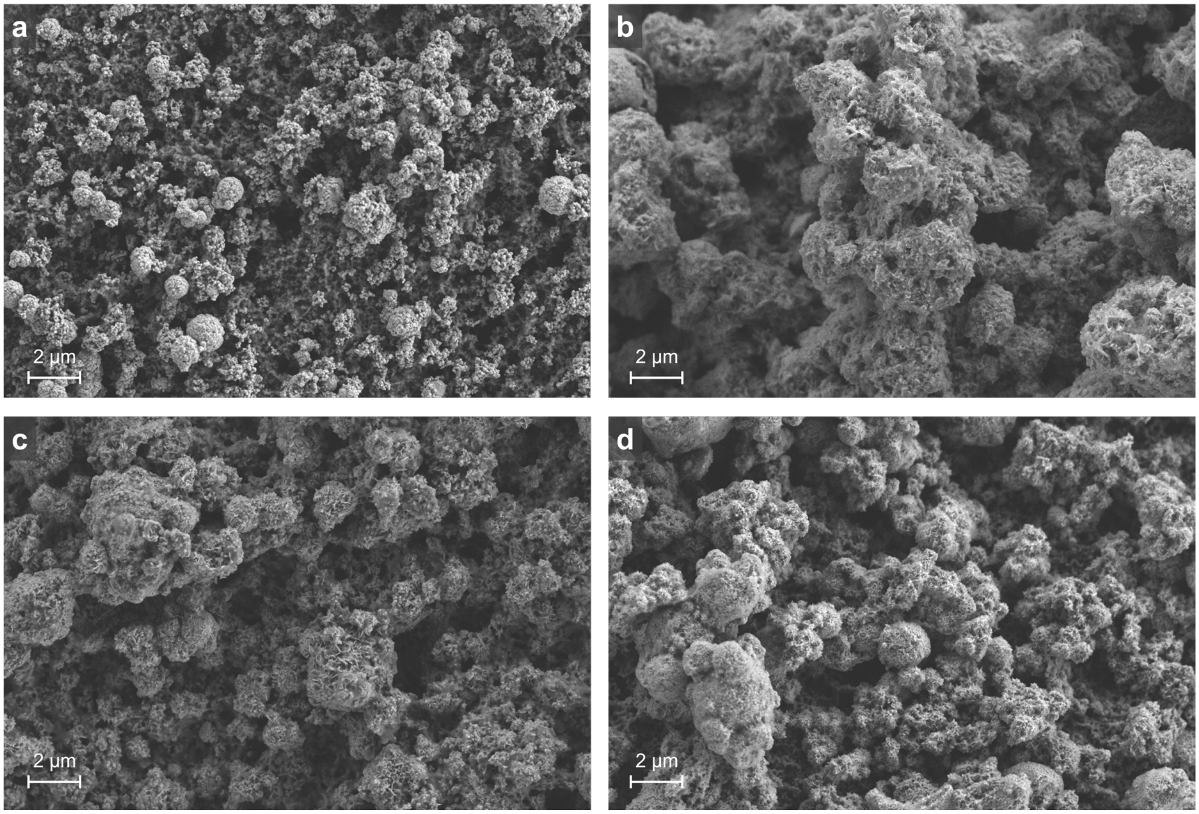

Figure S2. SEM images for Cu and Cu-PhR at 5.00 Kx. a) Cu, b) Cu-PhBr, c) Cu-PhOMe and d) Cu-$PhCl_2$.

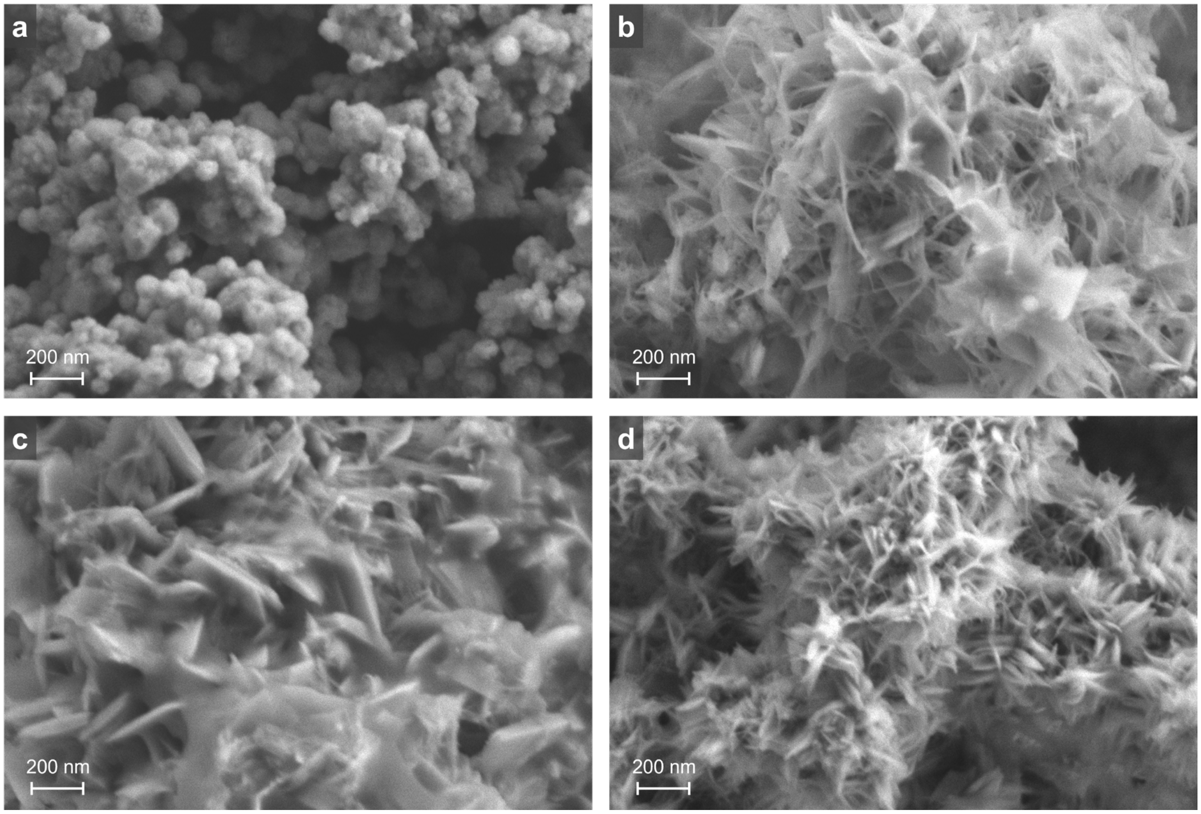

Figure S3. SEM images for Cu and Cu-PhR at 30.0 Kx. a) Cu, b) Cu-PhBr, c) Cu-PhOMe and d) Cu-$PhCl_2$.

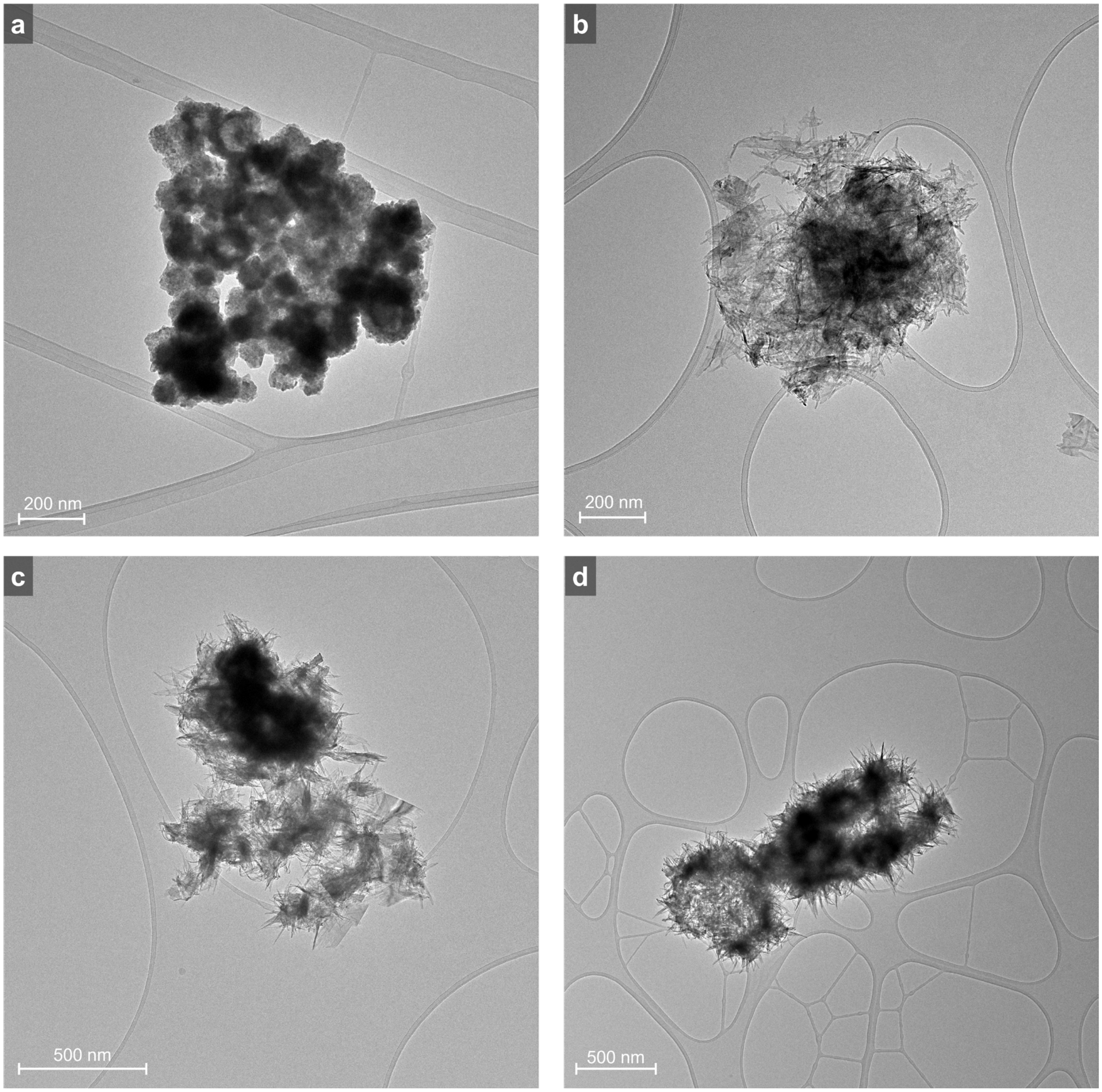

Figure S4. TEM images for Cu and Cu-PhR. a) Cu, b) Cu-PhBr, c) Cu-PhOMe and d) Cu-$PhCl_2$.

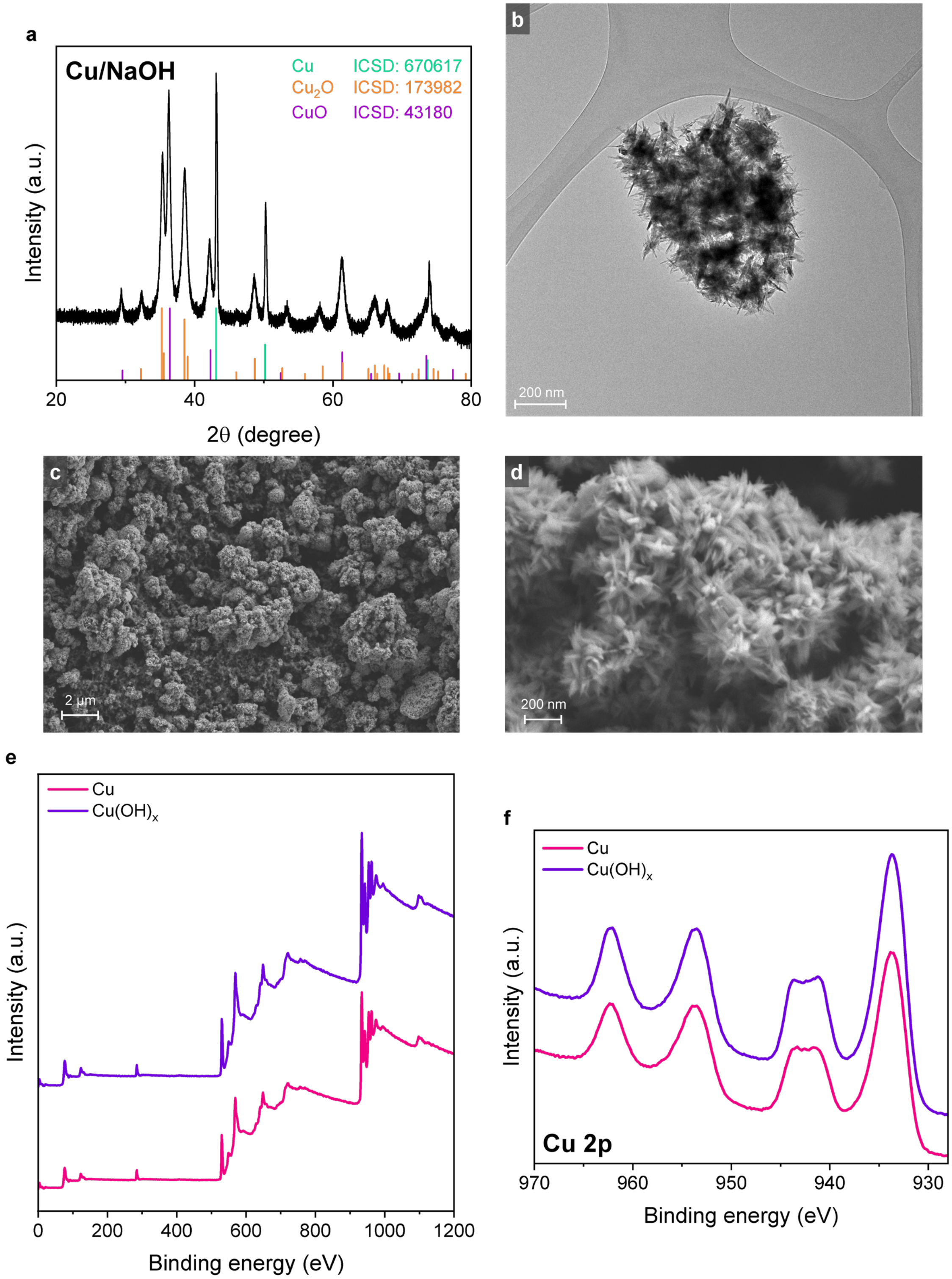

Figure S5. Material characterization of Cu-OH_{soaked}. a) XRD pattern. b) TEM image. SEM images at c) 5.00 Kx and d) 30.0 Kx. e) XPS spectra of Cu and Cu-OH_{soaked} at a range of binding energy. f) Cu 2p XPS spectra of Cu and Cu-OH_{soaked}.

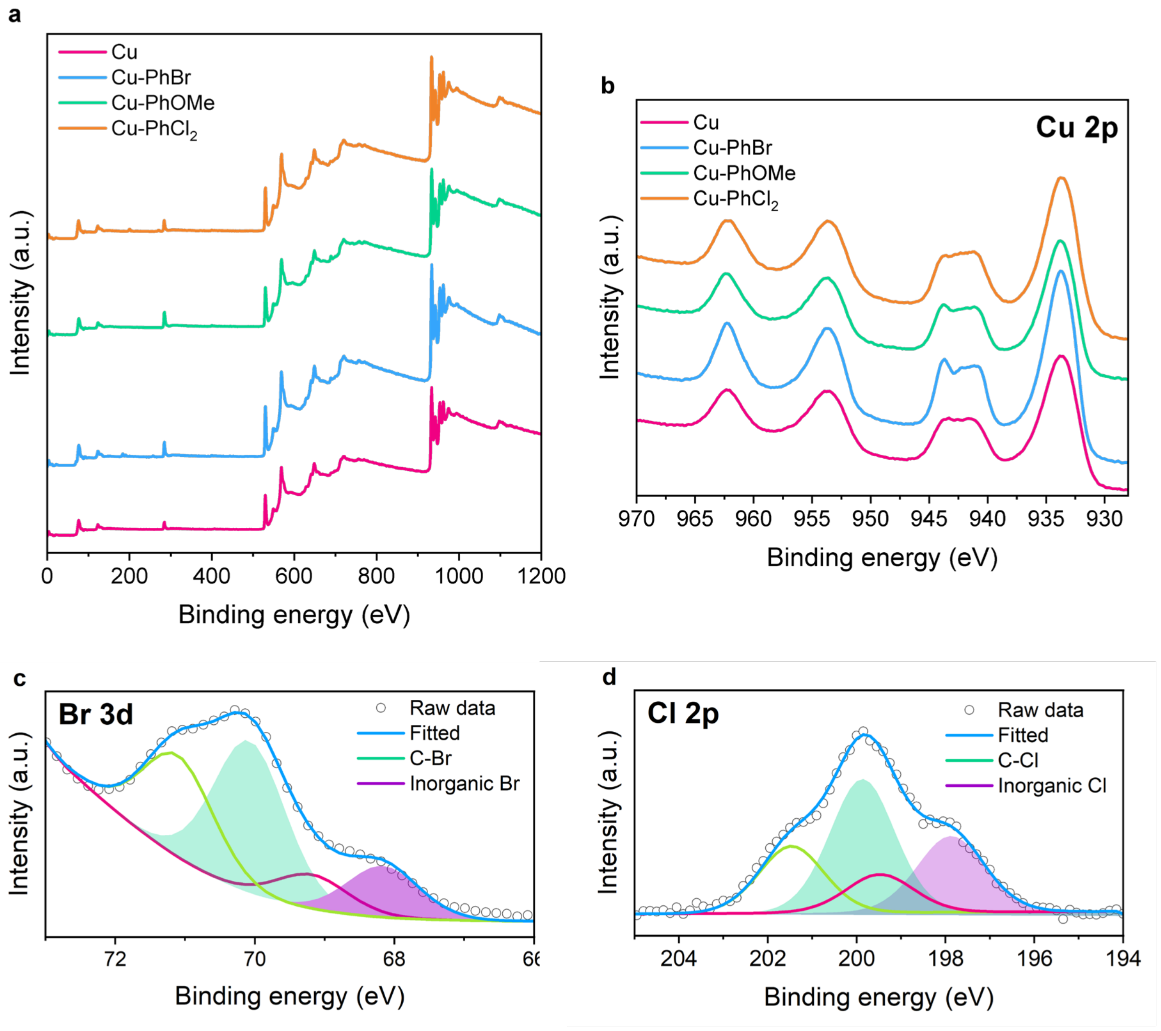

Figure S6. XPS for Cu and Cu-PhR. a) Wide XPS spectra and b) high resolution Cu 2p XPS spectra for Cu and Cu-PhR. c) Br 3d and d) Cl 2p XPS spectra of Cu-PhBr and Cu-$PhCl_2$ respectively. In addition to organic halogen (green), minor contribution from inorganic halogens is observed (purple). Due to the atmospheric contamination, O 1s of Cu-PhOMe could not be analyzed.

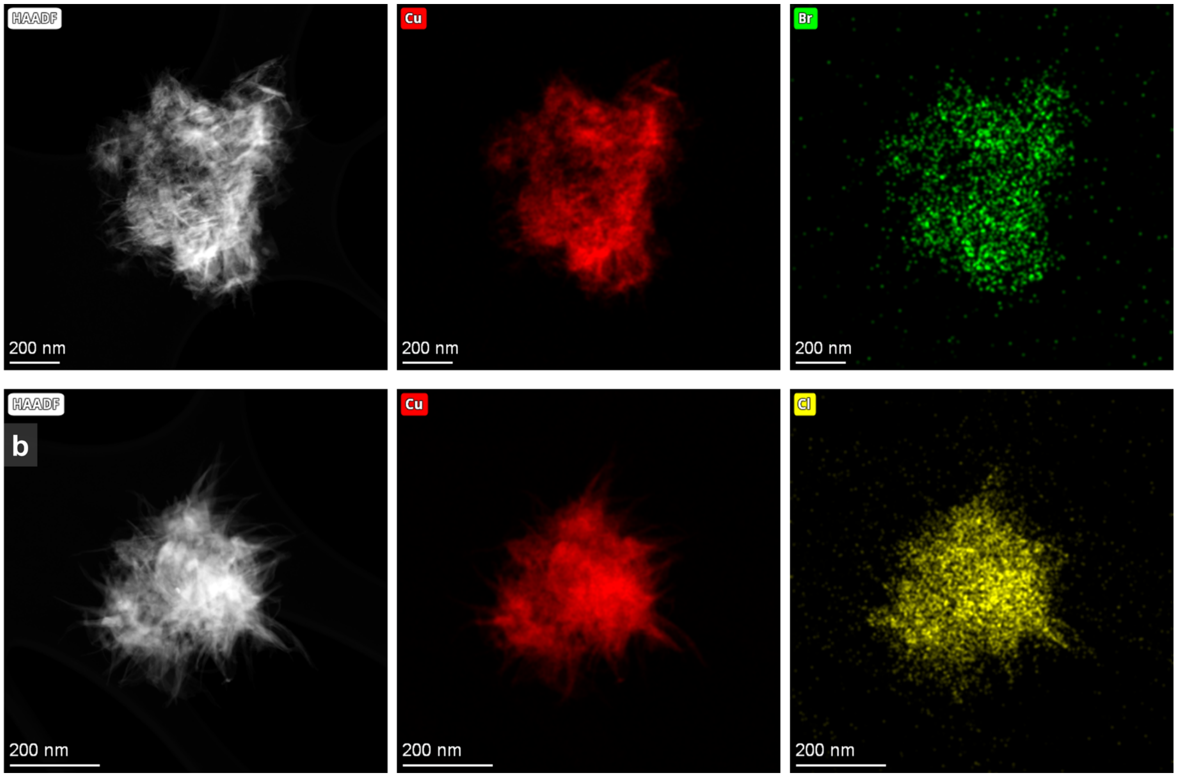

Figure S7. STEM images of Cu and Cu-PhR. EDX mapping for HAADF (left), Cu (middle) and heteroatom (right) for a) Cu-PhBr and b) Cu-$PhCl_2$.

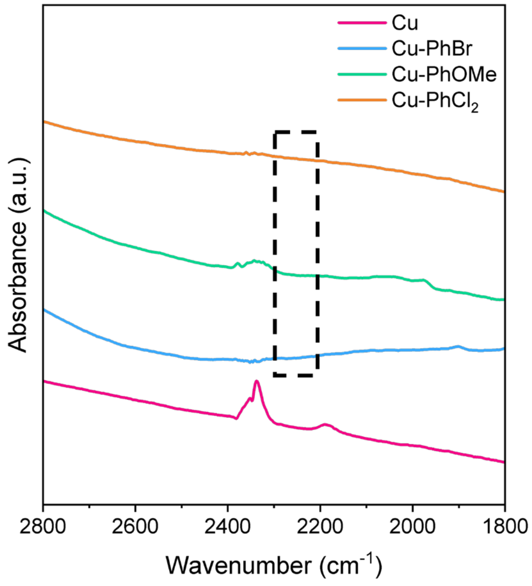

Figure S8. DRIFT spectra. DRIFTS for Cu and Cu-PhR between 1800 cm^{-1} and 2800 cm^{-1}. Signal for diazo group typically appears in the region marked as black box (between 2200 cm^{-1} and 2300 cm^{-1}).[7] The spectra of Cu-PhBr, Cu-PhOMe and Cu-$PhCl_2$ show the absence of this signal, signifying the absence of a diazonium salt. The signal at 2200 cm^{-1} on the Cu spectrum is characteristic of Cu oxide nanoparticles,[8,9] which may be masked by the organic moities from the modification reaction.

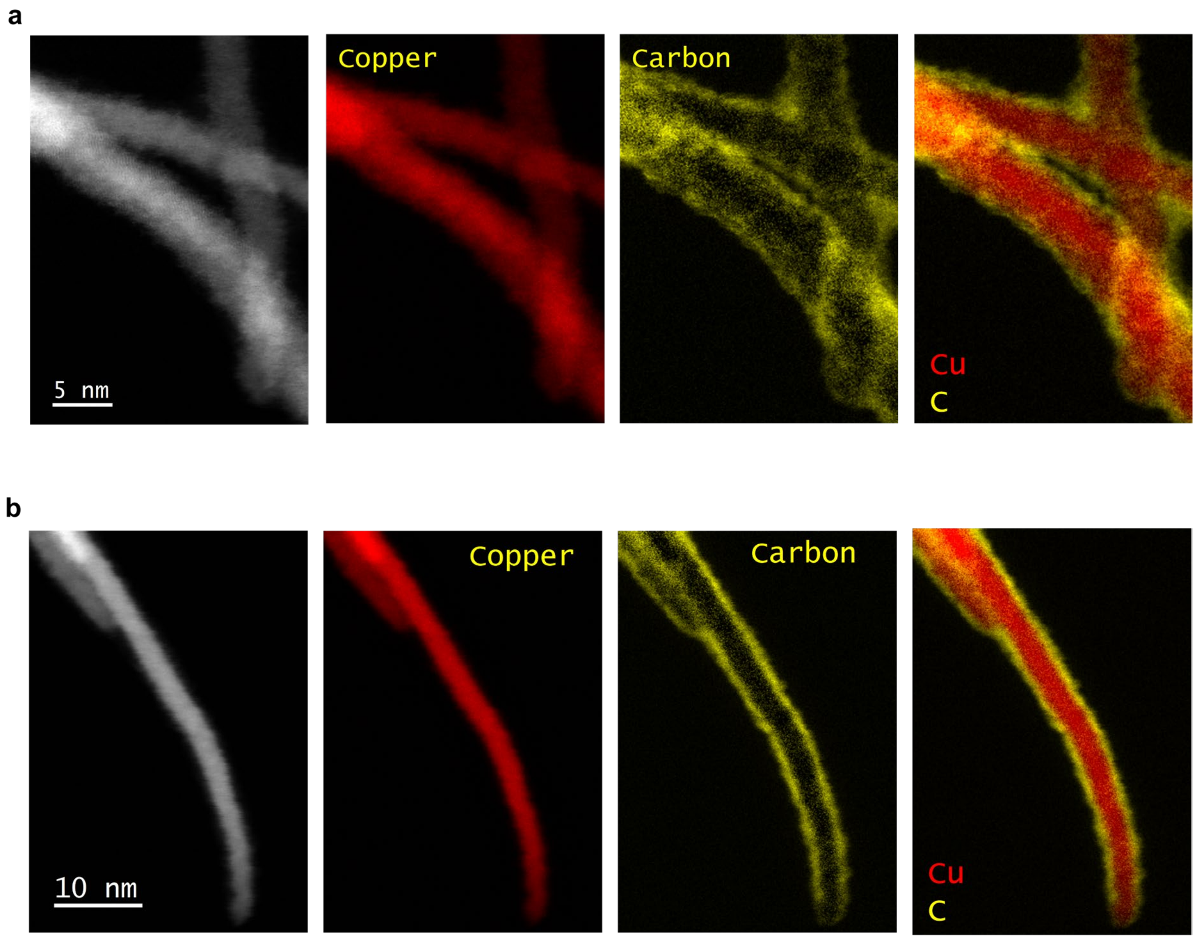

Figure S9. EELS mapping images. ADF image (left) and the corresponding Cu (second to left), C (third to left) and merged (right) EELS elemental maps on a) Cu-PhOMe and b) Cu-$PhCl_2$ using aberration corrected HR-STEM. The thickness of the carbon layer was determined to be for 1 - 1.2 nm Cu-PhOMe and 1 nm - 1.4 nm for Cu-$PhCl_2$.

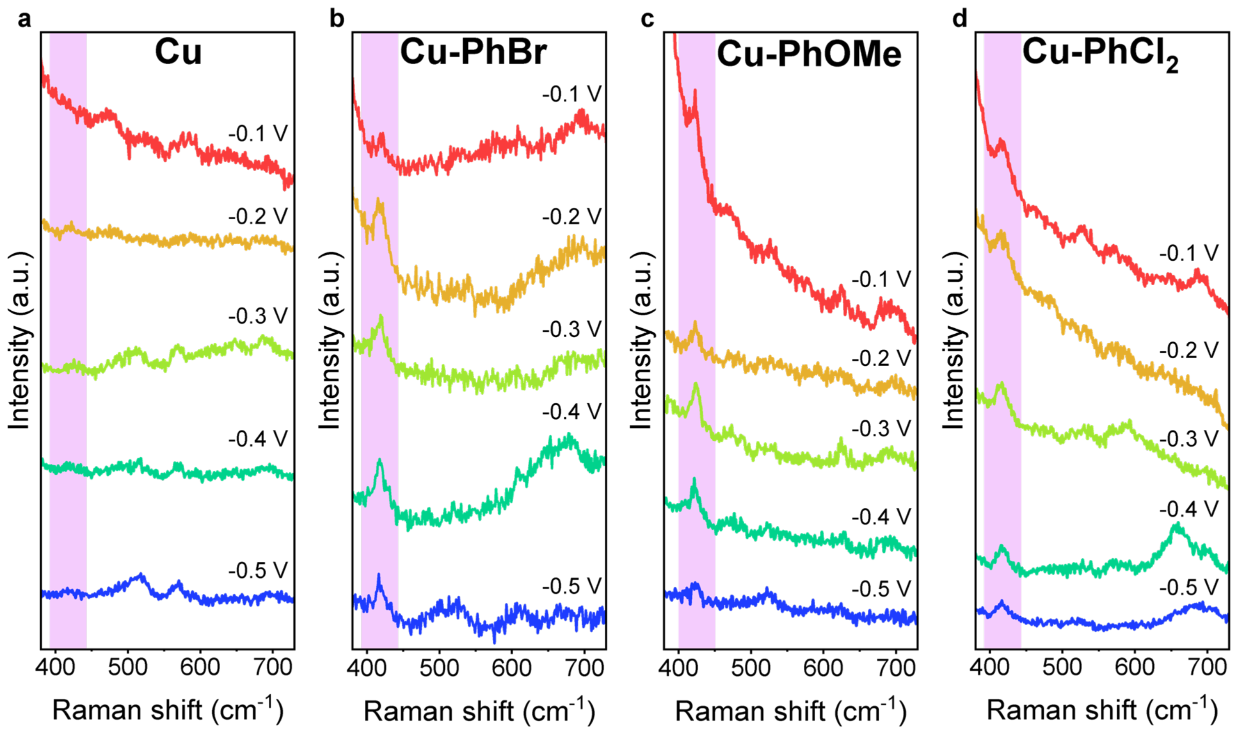

Figure S10. ***In-situ*** **SERS spectra for ν(Cu−C) signal in CO_2-saturated 0.1 M $KHCO_3$ (pH = 6.8)** for a) Cu, b) Cu-PhBr, c) Cu-PhOMe and Cu-$PhCl_2$. Spectra at more negative potentials were not acquired due to bubble evolution.

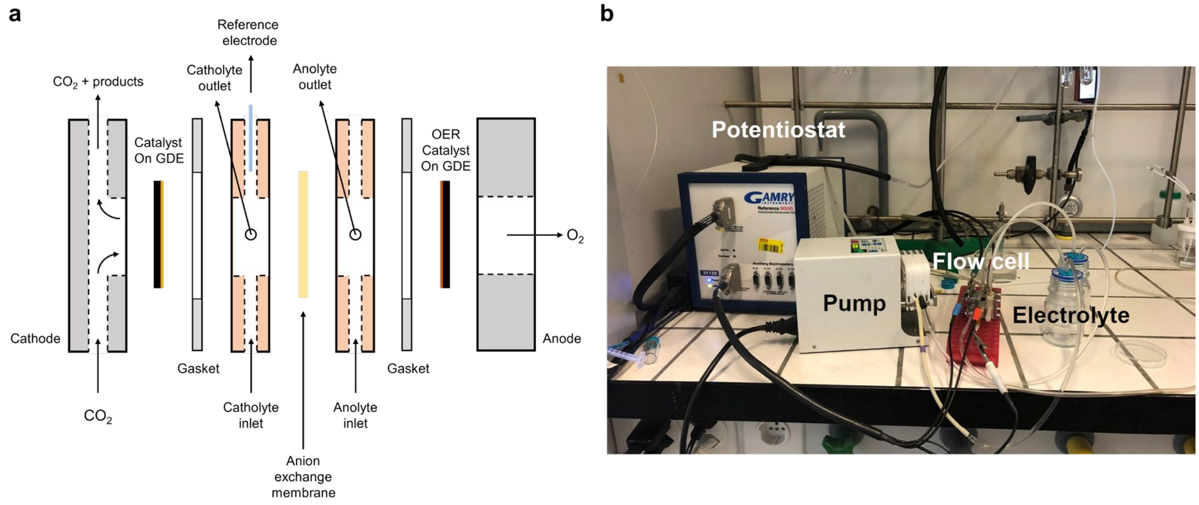

Figure S11. Experimental CO_2RR setup. a) Schematic illustration of a flow cell equipped with GDE. b) A typical experimental setup image for electrochemical measurement with a flow cell purged with anolyte and catholyte (1 M KOH) in liquid chamber and connected with a potentiostat via metallic connections. The gas inlet is connected to CO_2 gas line and outlet to a GC.

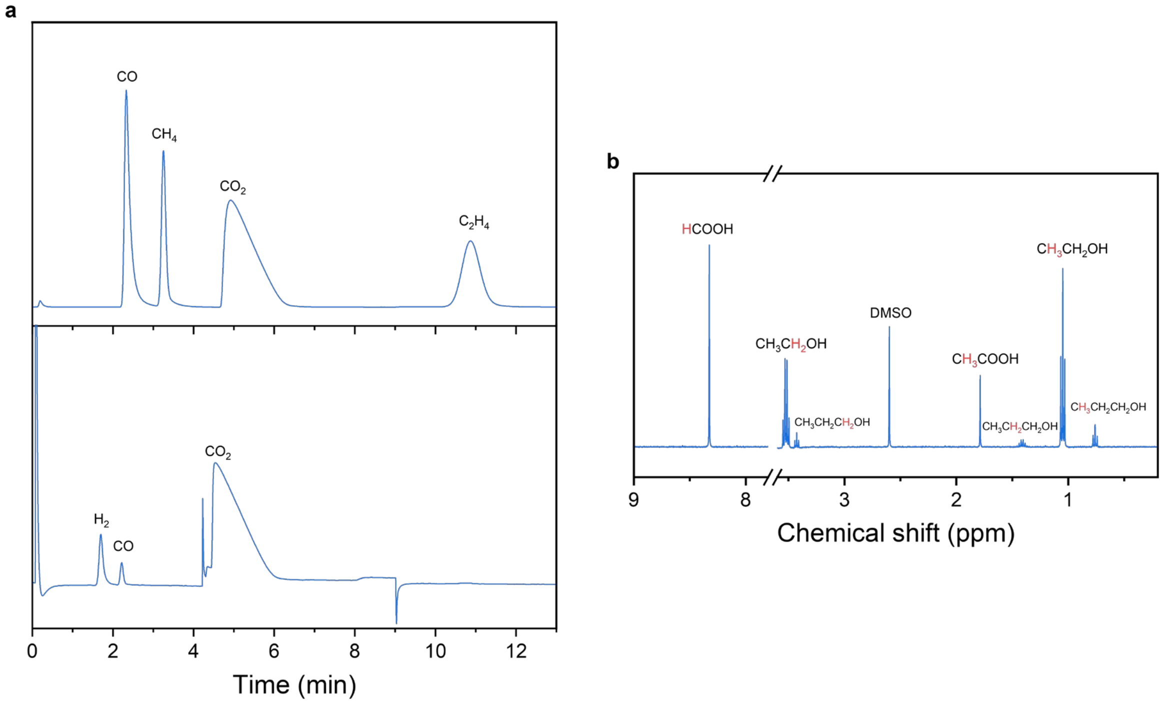

Figure S12. CO_2RR product quantification. A typical GC chromatogram using a) FID (top) and TCD (bottom) as well as b) NMR spectrum. The peak identification for GC is done by calibrating using a gas mixture with a known composition.

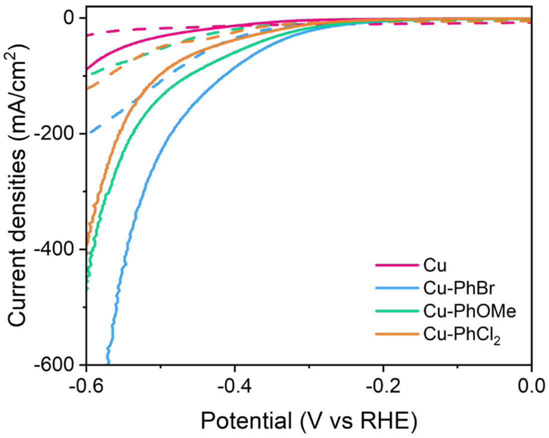

Figure S13. LSV of Cu and Cu-PhR. LSV curves obtained under CO_2 (solid) or N_2 (dashed) purged condition at a scan rate of 10 mV s^{-1} in 1 M KOH.

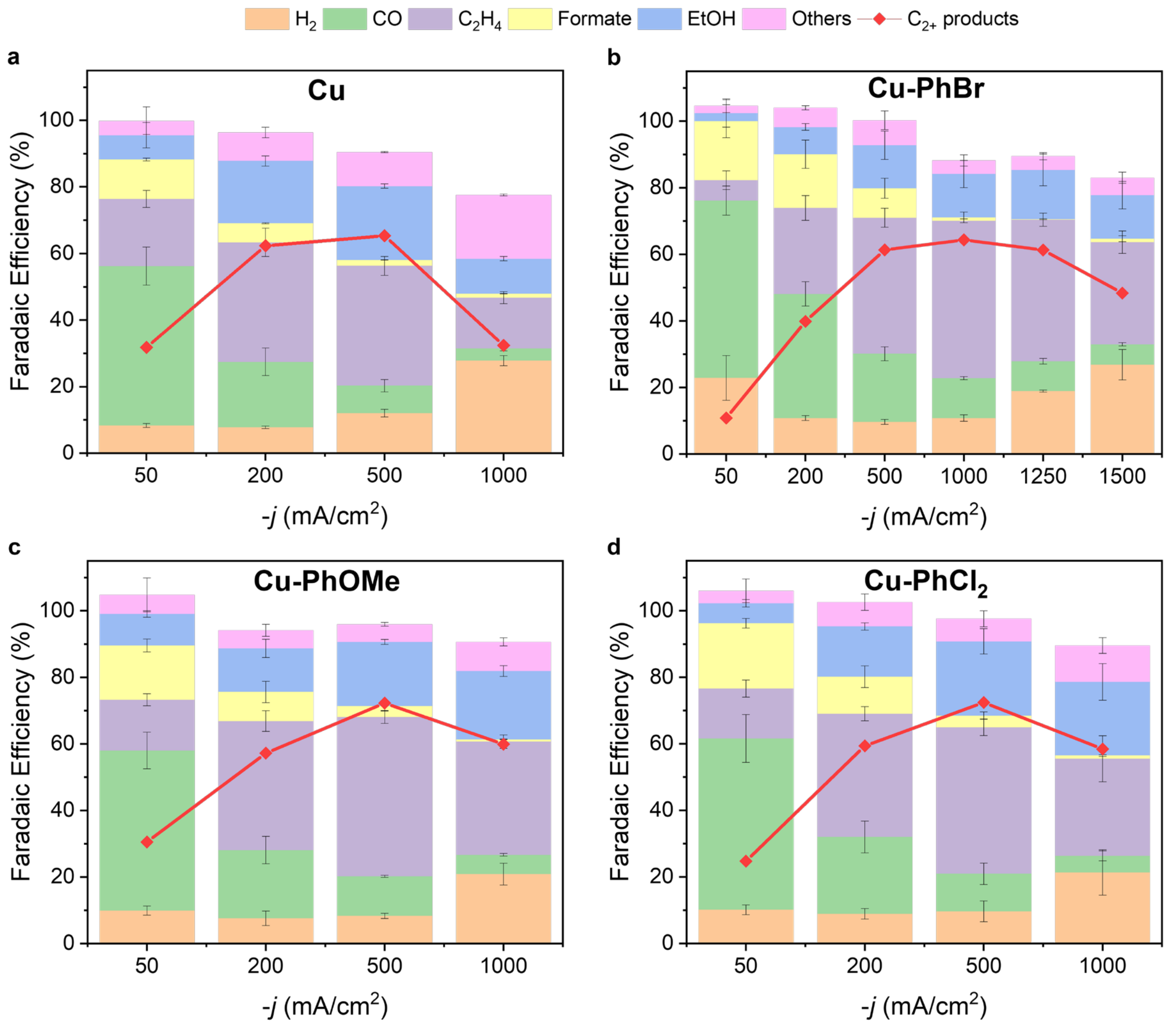

Figure S14. **Selectivity of CO_2RR at different applied current densities.** Faradaic efficiency of a) Cu, b) Cu-PhBr, c) Cu-PhOMe and d) Cu-$PhCl_2$ under different current densities in 1 M KOH. The experiment for each electrode was performed three times to obtain the mean and the standard deviation as the error bar.

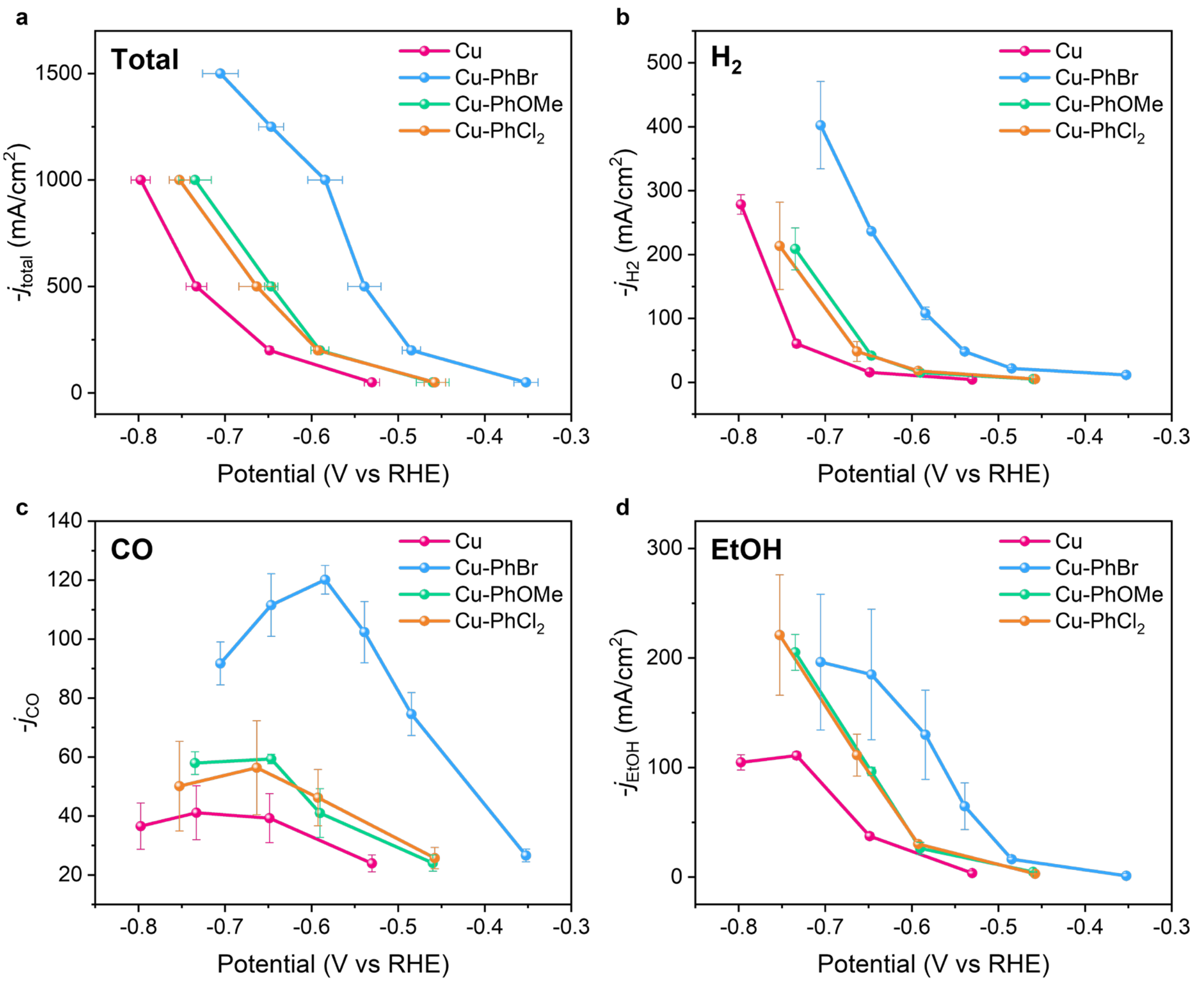

Figure S15. Activity of CO_2RR at different applied current densities. a) Total current density of Cu and Cu-PhR in 1 M KOH. Partial current densities of Cu and Cu-PhR for b) H_2, c) CO and d) EtOH under different current densities in 1 M KOH. The experiment for each electrode was performed three times to obtain the mean and the standard deviation as the error bar.

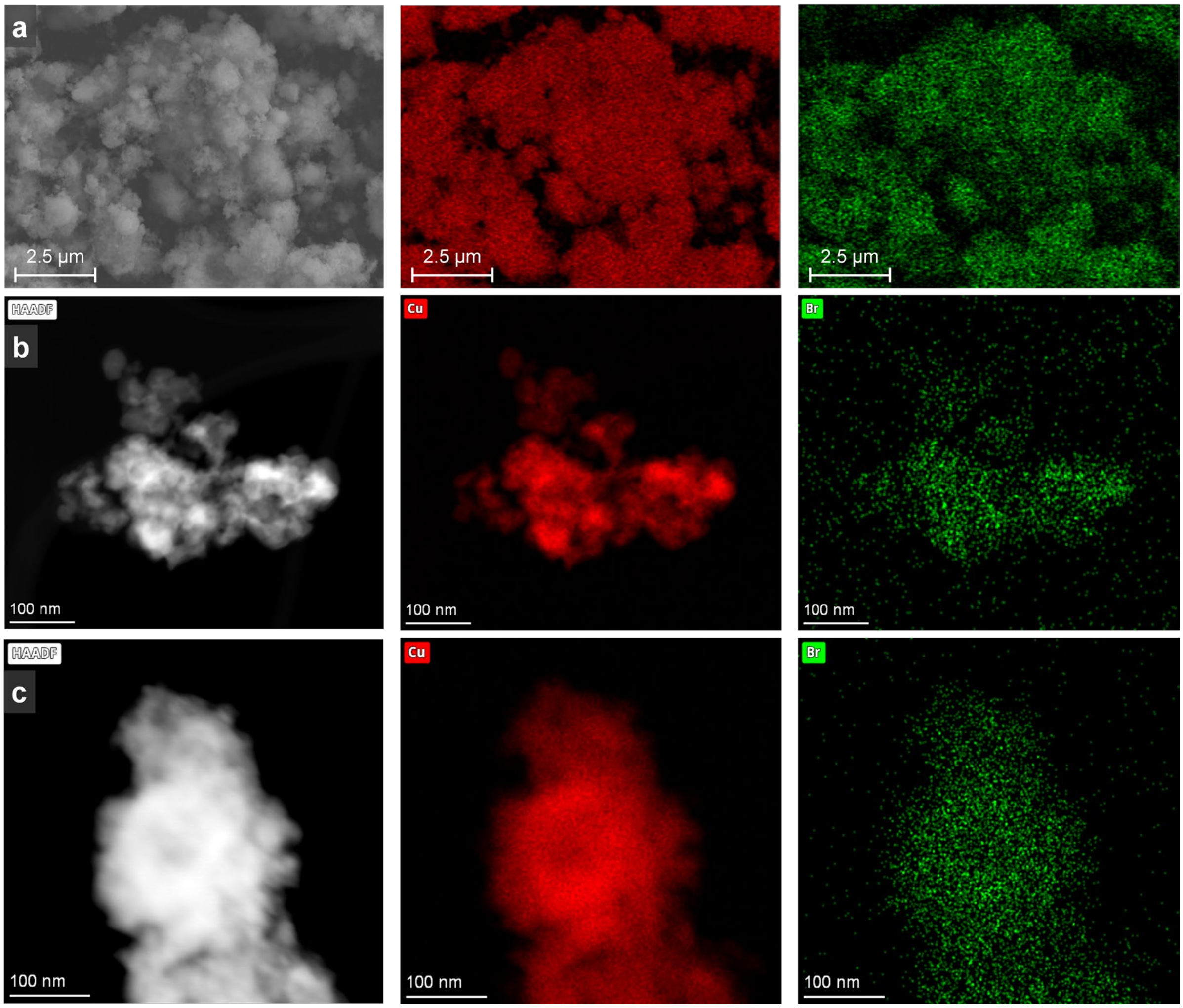

Figure S16. Post-catalytic characterization of Cu-PhBr. a) SEM image (left) and EDX mapping of Cu (middle, red) and Br (right, green) for Cu-PhBr after CO_2RR. b) and c) STEM images and EDX mapping for HAADF (left), Cu (middle, red) and Br (right, green) for Cu-PhBr after CO_2RR.

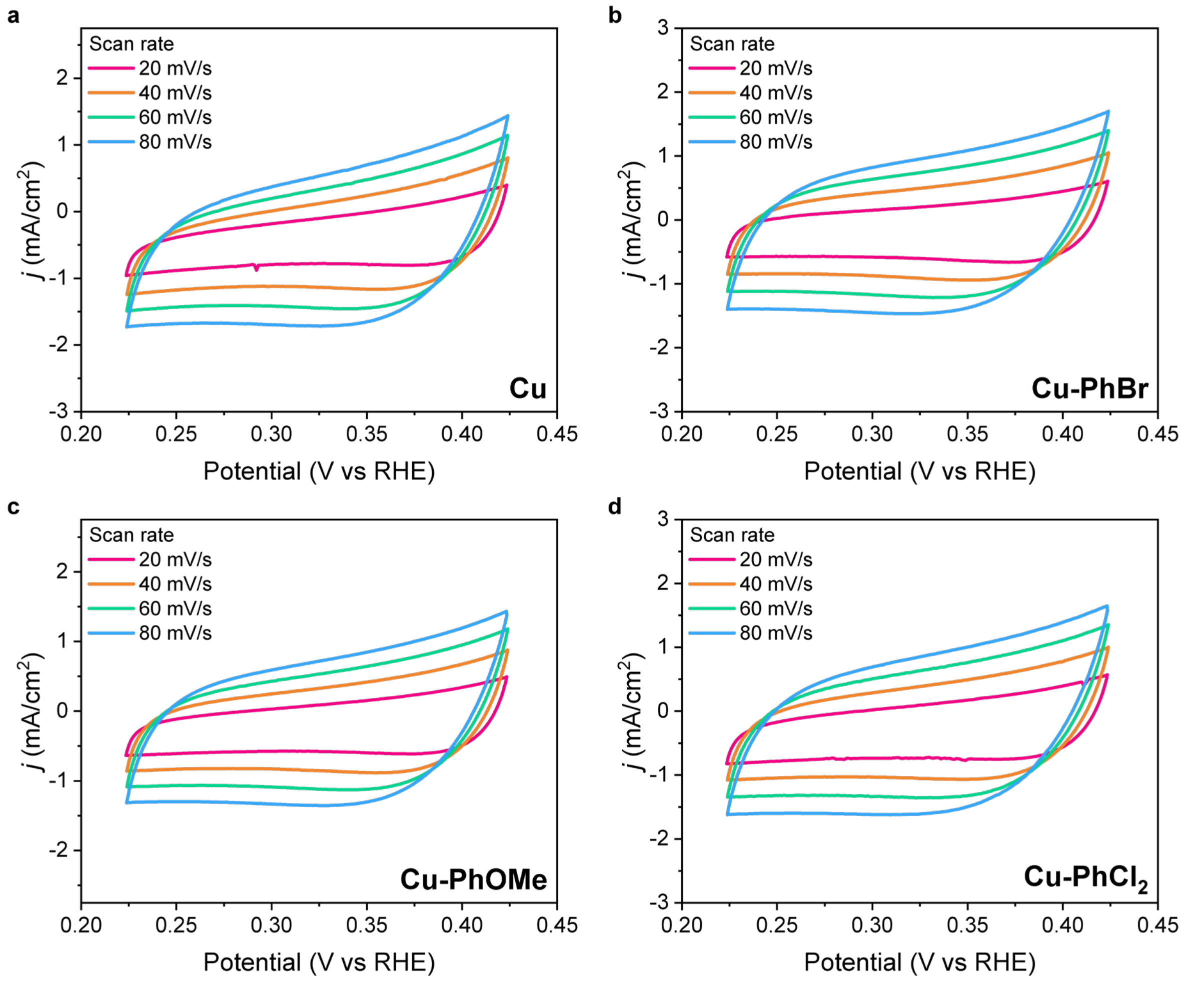

Figure 17. Double layer capacitance measurements. Typical CV curves at different scanning rates in 1 M KOH for a) Cu, b) Cu-PhBr, c) Cu-PhOMe and d) Cu-$PhCl_2$.

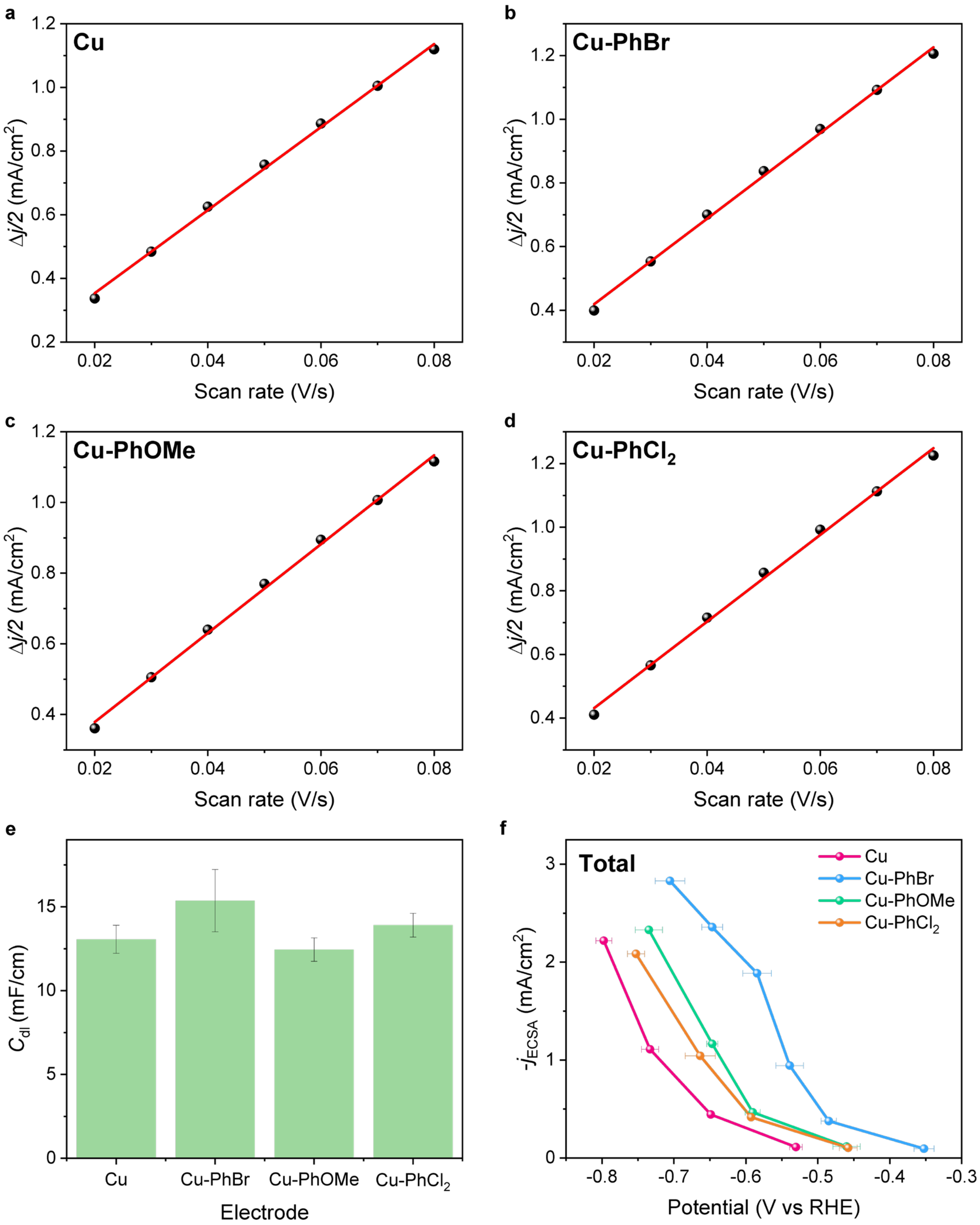

Figure S18. Double layer capacitance measurements. Linear fitting of the difference between anodic and cathodic scan as a function of scanning rate for a) Cu, b) Cu-PhBr, c) Cu-PhOMe and d) Cu-$PhCl_2$. e) Double layer capacitance (C_{dl}) of Cu and Cu-PhR. The experiment for each electrode was performed three times to obtain the mean and the standard deviation as the error bar. The values of C_{dl} and electrochemically active surface area (ECSA) are tabulated in Table S2. f) j_{total} normalized by ECSA from Table S2.

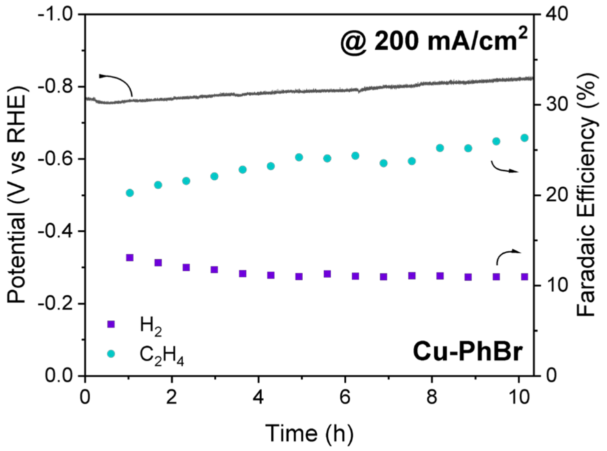

Figure S19. Stability of Cu-PhBr. Stability test was measured in 0.5 M $KHCO_3$ at 200 mA cm^{-2} for 10 h. The electrolyte was not refreshed during 10 h operation.

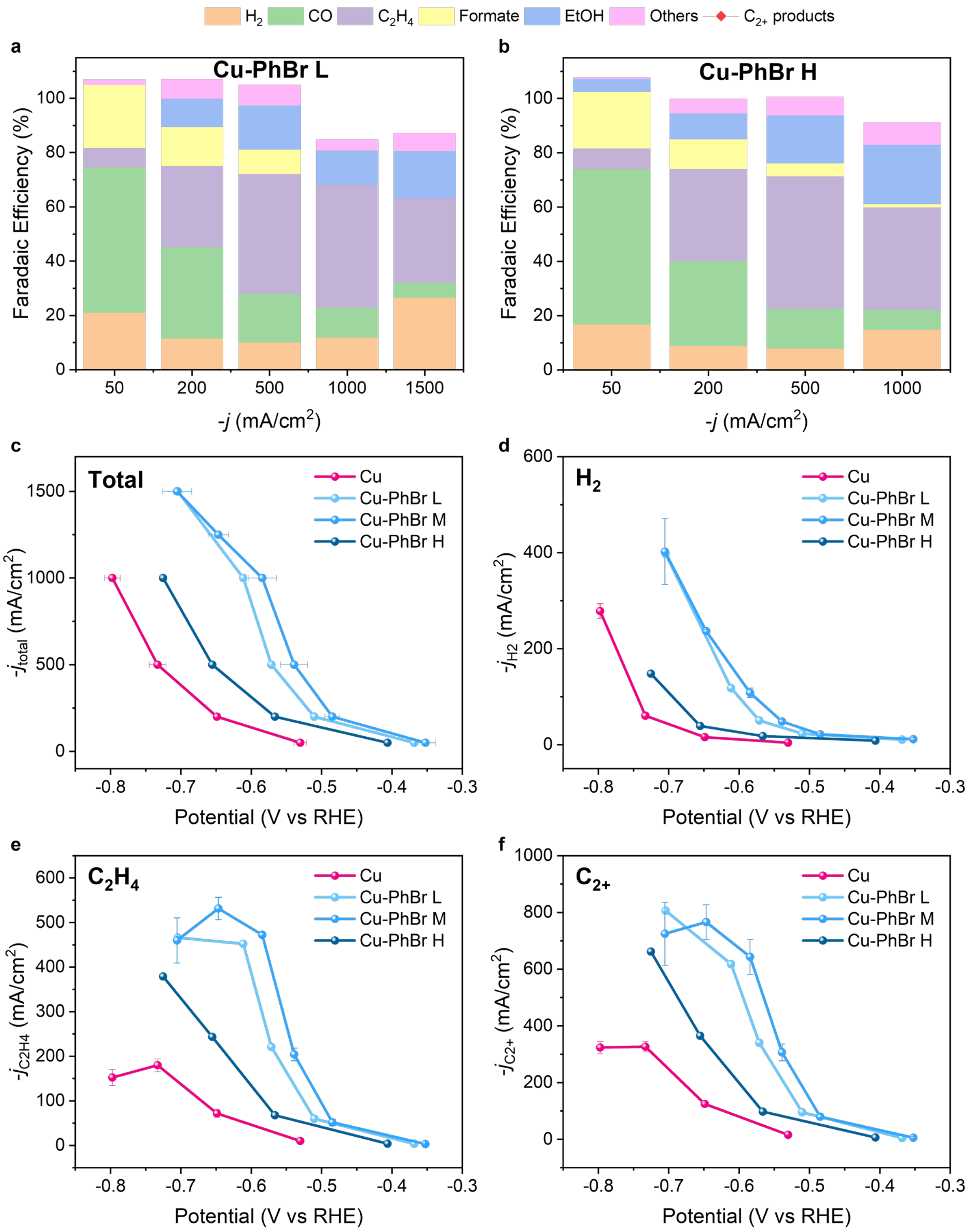

Figure S20. CO_2RR performance of Cu-PhBr at different reagent loading. Faradaic efficiency of a) Cu-PhBr low loading (L) and b) Cu-PhBr high loading (H) at different applied current densities. b) Total current density of Cu-PhBr at different loading in 1 M KOH. Partial current densities of Cu-PhBr at different loading for d) H_2, e) C_2H_4 and f) C_{2+} under different current densities in 1 M KOH.

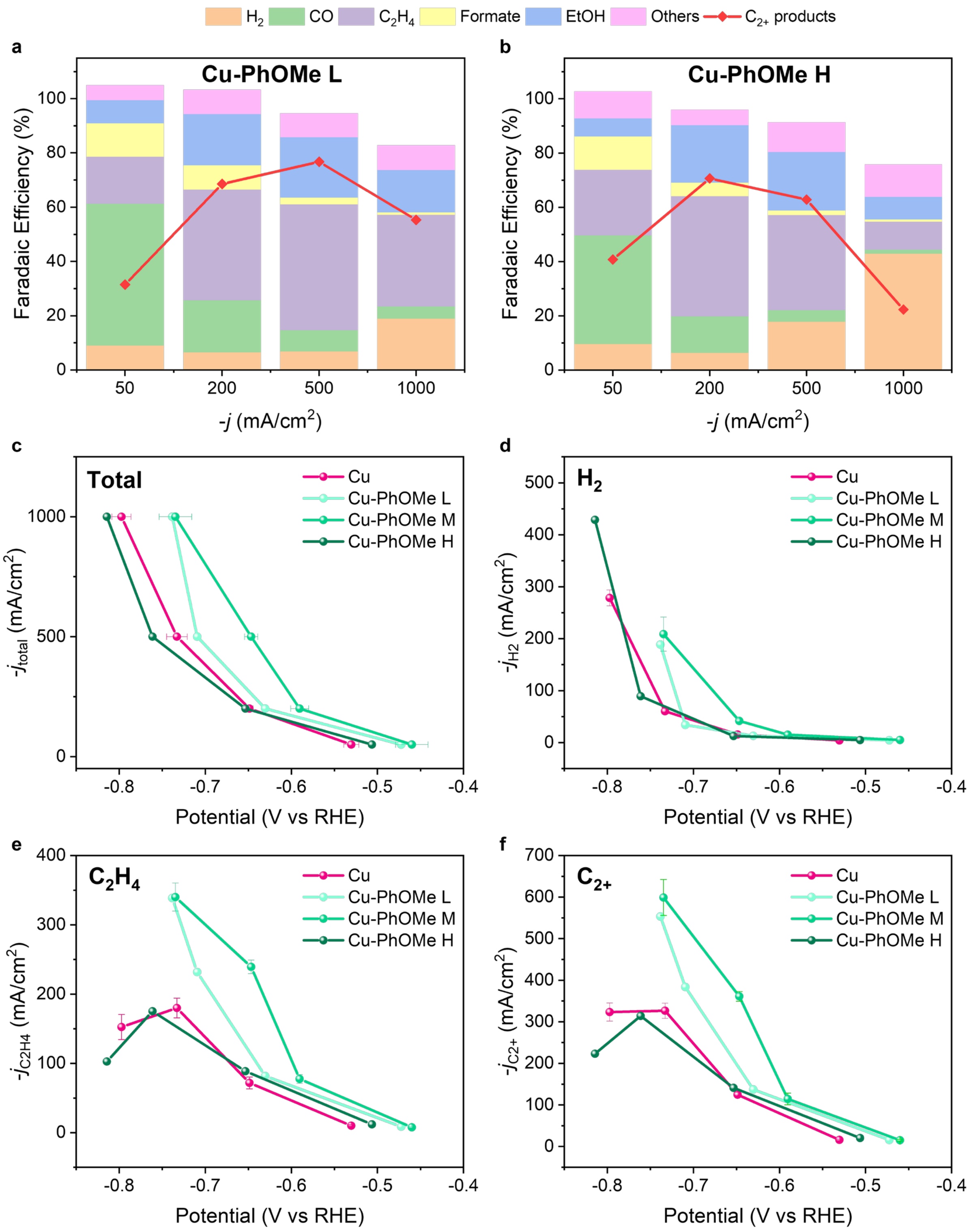

Figure S21. CO_2RR performance of Cu-PhOMe at different reagent loading. Faradaic efficiency of a) Cu-PhOMe low loading (L) and b) Cu-PhOMe high loading (H) at different applied current densities. b) Total current density of Cu-PhOMe at different loading in 1 M KOH. Partial current densities of Cu-PhOMe at different loading for d) H_2, e) C_2H_4 and f) C_{2+} under different current densities in 1 M KOH.

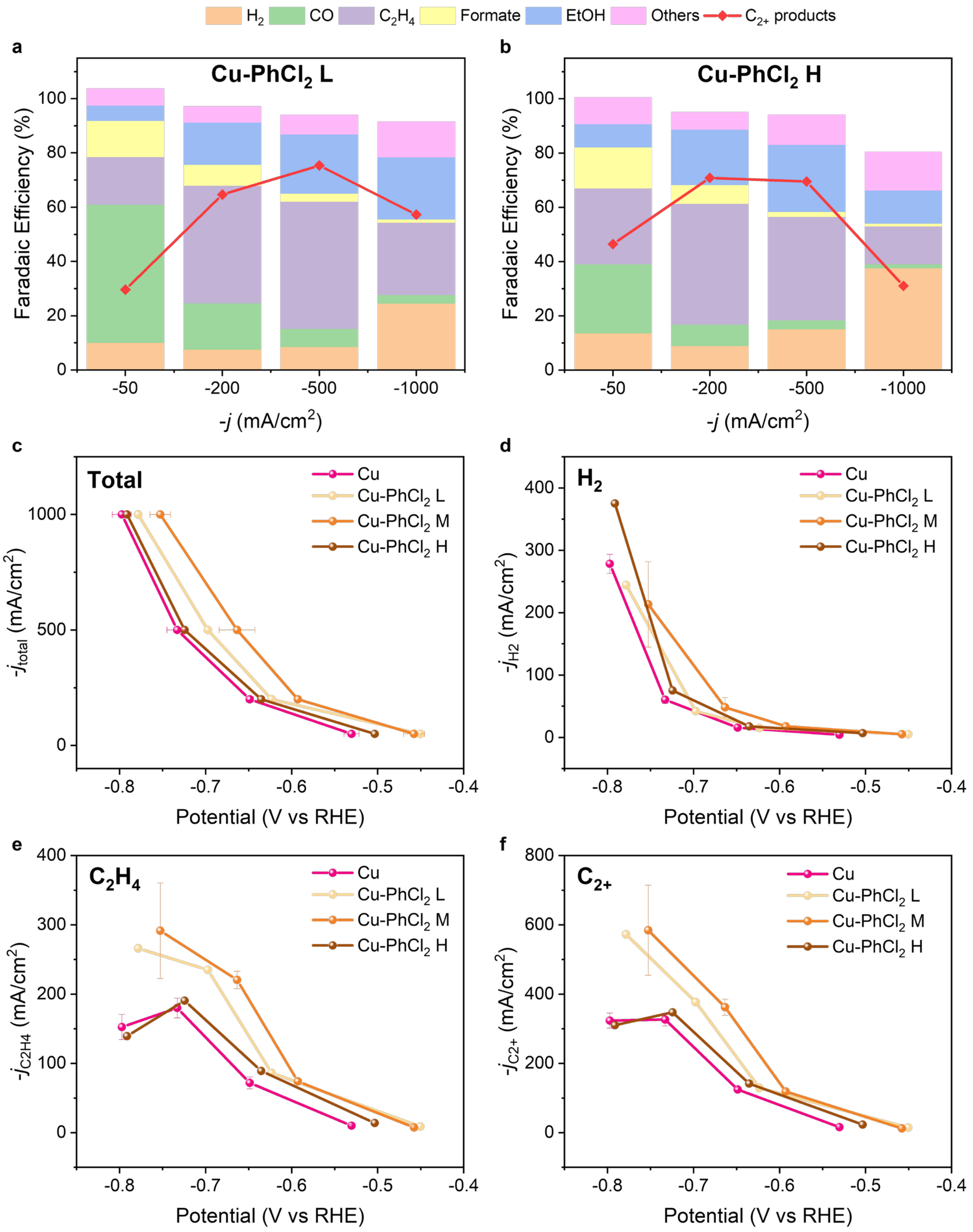

Figure S22. CO_2RR performance of Cu-PhCl_2 at different reagent loading. Faradaic efficiency of a) Cu-PhCl_2 low loading (L) and b) Cu-PhCl_2 high loading (H) at different applied current densities. b) Total current density of Cu-PhCl_2 at different loading in 1 M KOH. Partial current densities of Cu-PhCl_2 at different loading for d) H_2, e) C_2H_4 and f) C_{2+} under different current densities in 1 M KOH

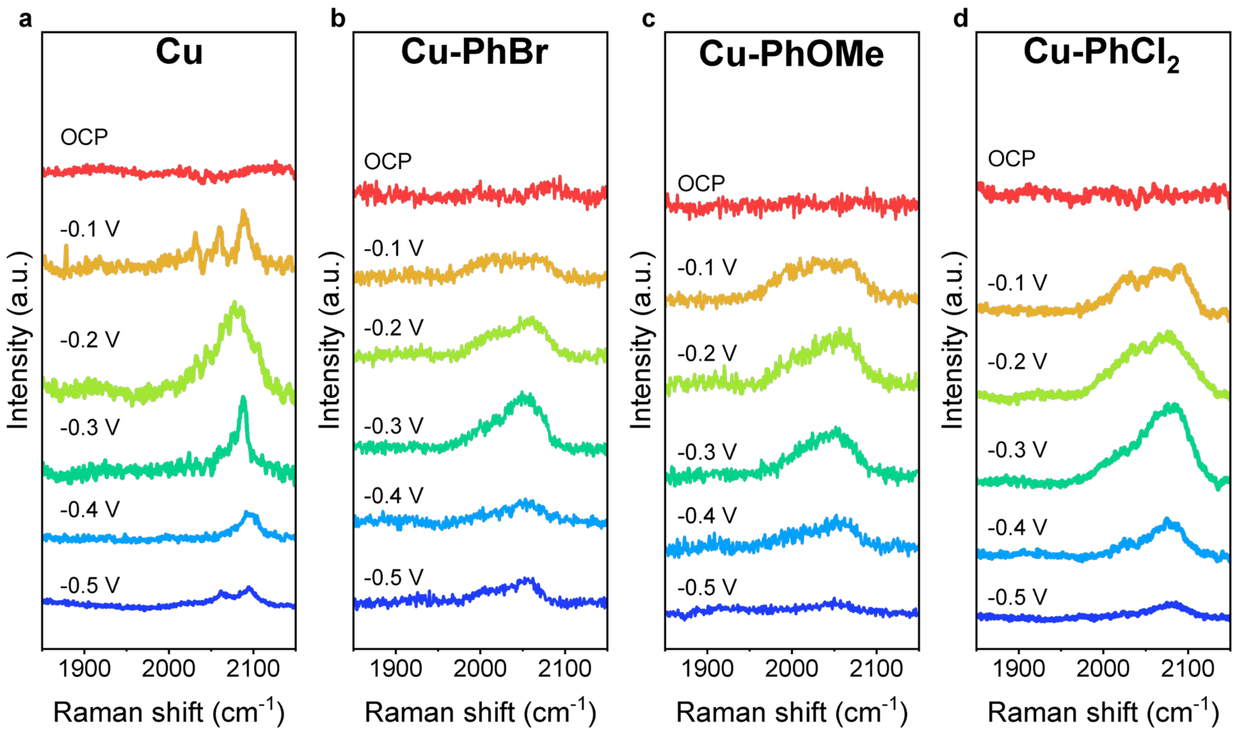

Figure S23. **In-situ SERS spectra for ν(C−O) signal in CO_2-saturated 0.1 M $KHCO_3$ (pH = 6.8)** for a) Cu, b) Cu-PhBr, c) Cu-PhOMe and Cu-PhCl2. Spectra at more negative potentials were not acquired due to bubble evolution.

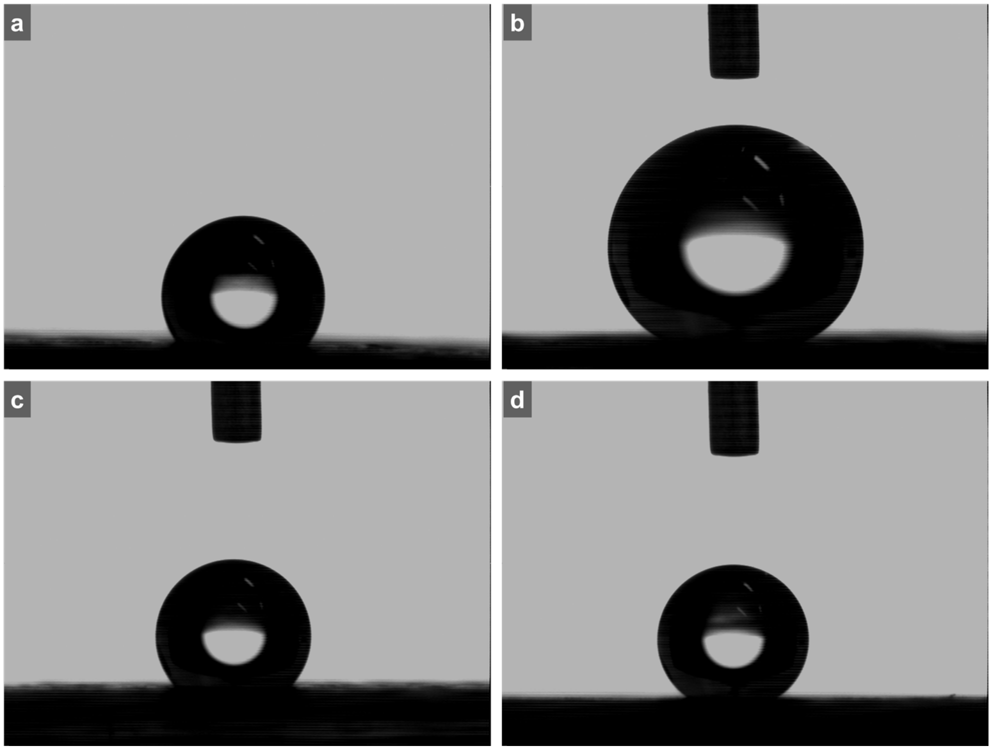

Figure S24. Contact angle measurement. Contact angle measurement between electrode surface and water droplet for a) Cu, b) Cu-PhBr, c) Cu-PhOMe and d) Cu-$PhCl_2$.

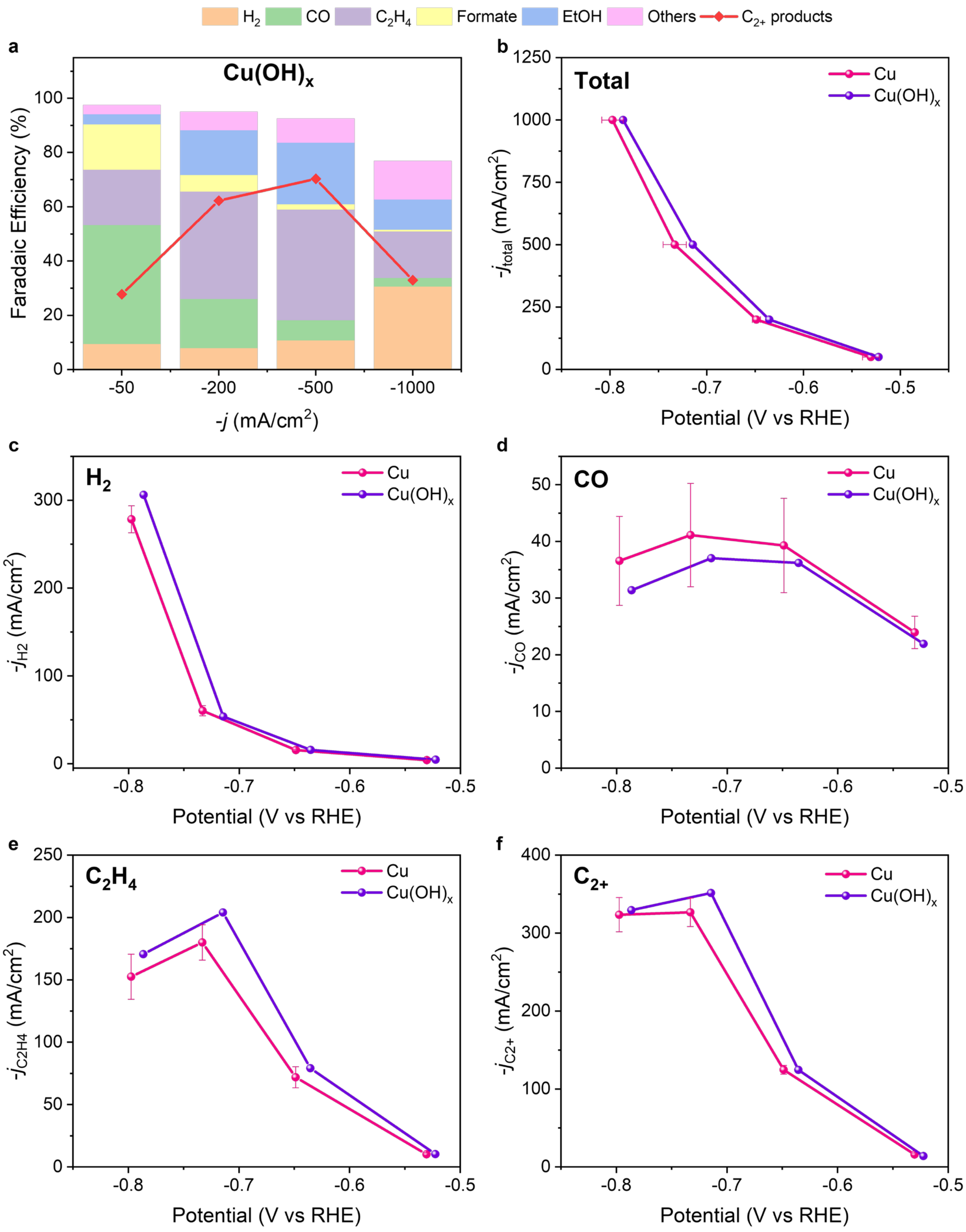

Figure 25. CO_2RR performance of Cu-OH$_{soaked}$. a) Faradaic efficiency of Cu-OH$_{soaked}$ in 1 M KOH. b) Total current density and partial current densities of Cu and Cu-OH$_{soaked}$ for c) H_2, d) CO, e) C_2H_4 and f) C_{2+} under different current densities in 1 M KOH.

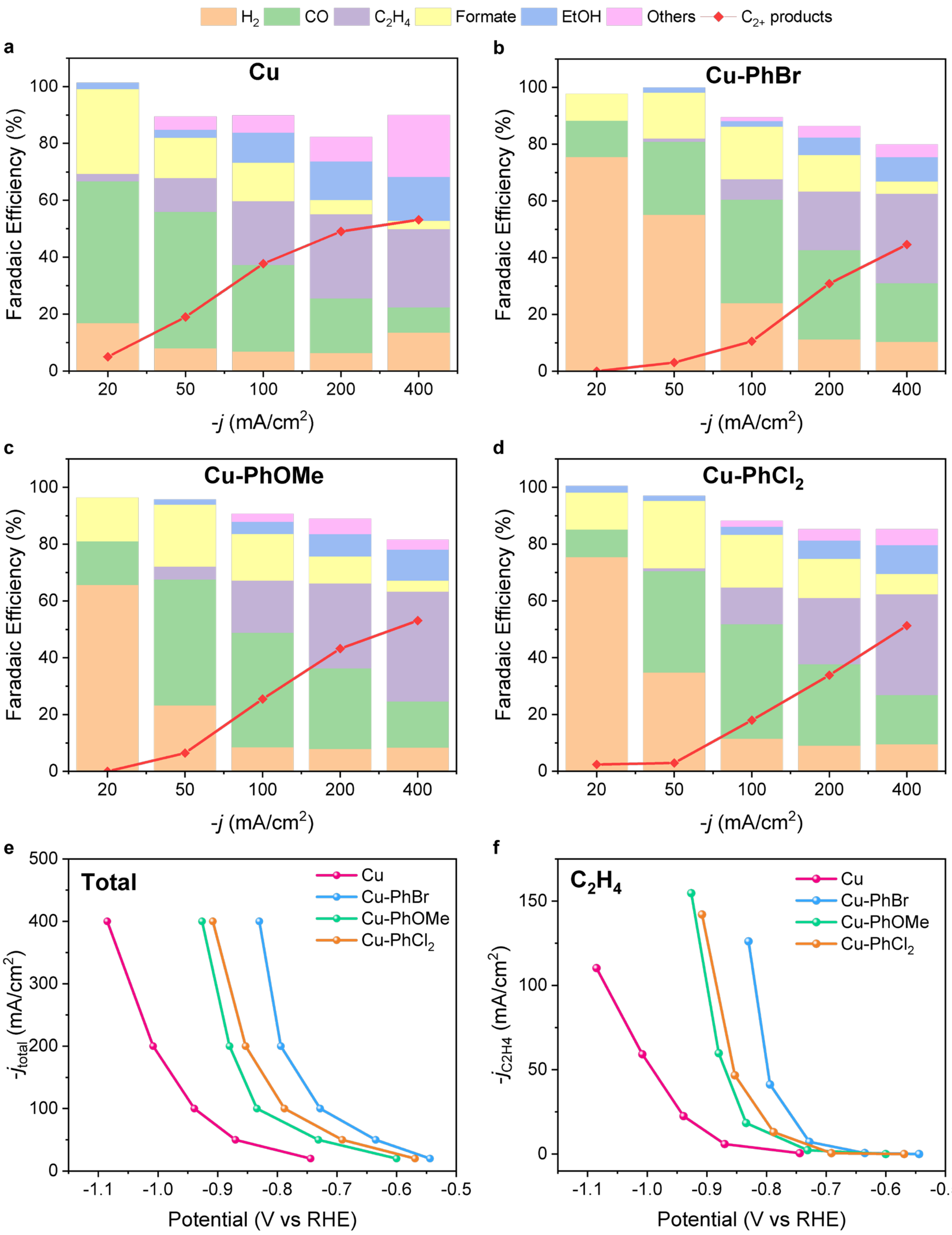

Figure S26. CO_2RR performance of bare Cu and Cu-PhR at different applied current densities in 0.5 M $KHCO_3$. Faradaic efficiency of a) Cu, b) Cu-PhBr, c) Cu-PhOMe and d) Cu-$PhCl_2$ in in 0.5 M $KHCO_3$. e) Total current density and f) partial current densities for C2H4 under different current densities in 0.5 M $KHCO_3$.

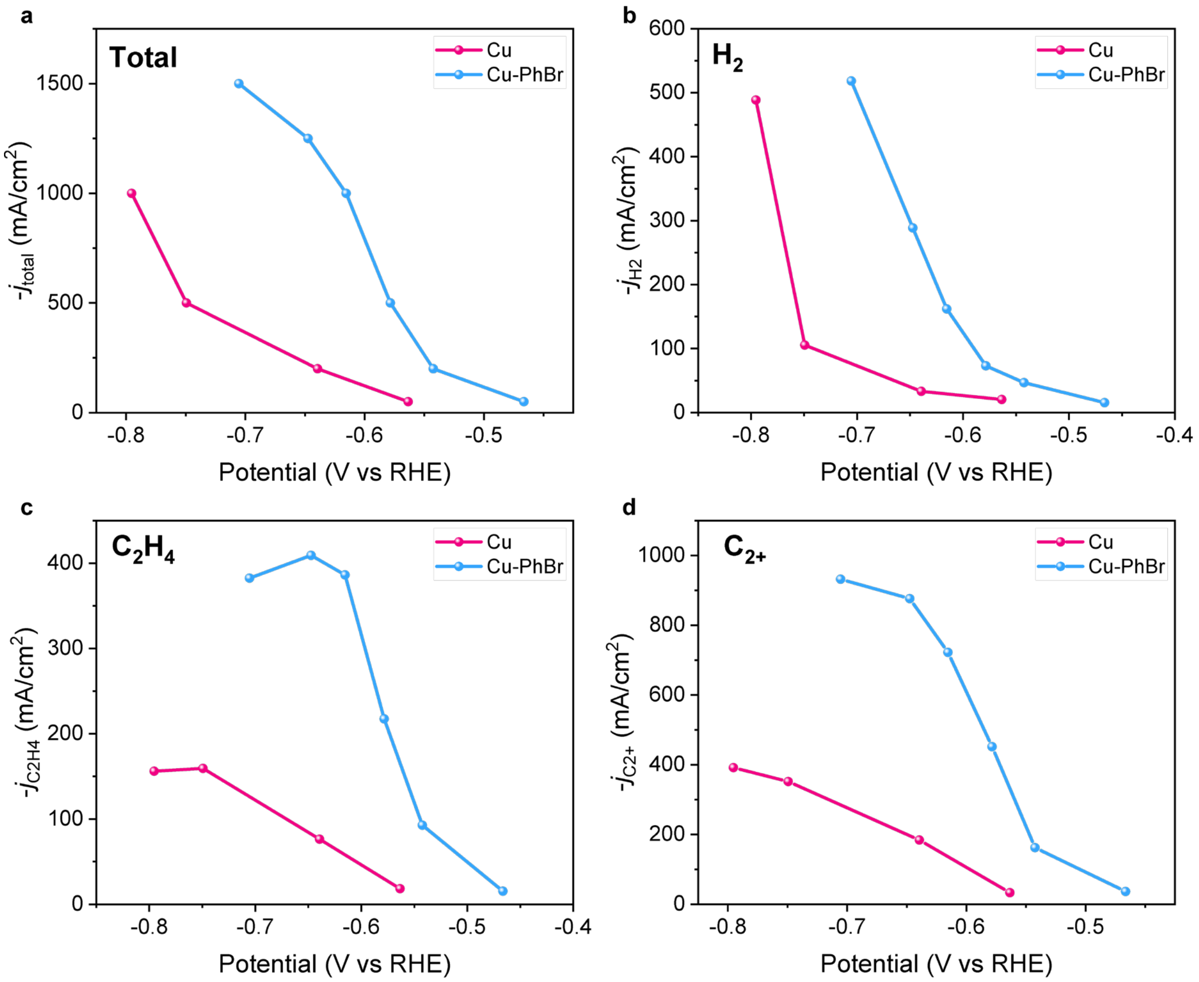

Figure S27. CORR performance of bare Cu and Cu-PhB at different applied current densities in 1 M KOH. a) Total current density and partial current densities for b) H_2, c) C_2H_4 and d) C_{2+} under different current densities 1 M KOH.

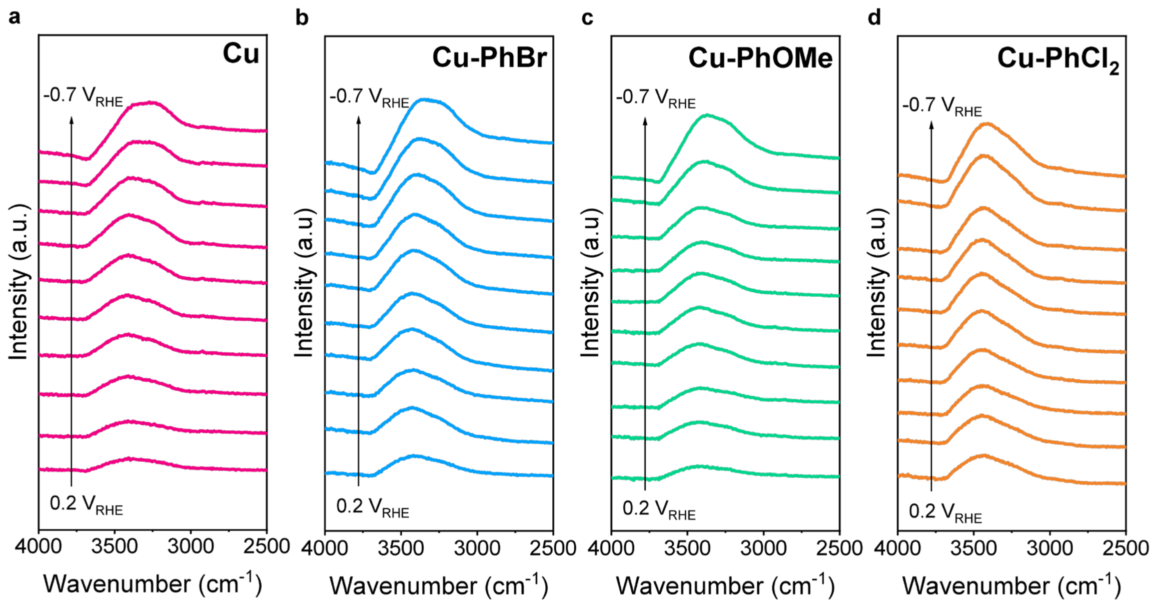

Figure S28. Raw *operando* ATR-SEIRAS spectra for ν(O–H) of interfacial H_2O in CO_2-saturated 0.1 M $KHCO_3$ (pH = 6.8). Raw spectra from 0.2 V_{RHE} to -0.7 V_{RHE} with a step of 0.1 V for a) Cu, b) Cu-PhBr, c) Cu-PhOMe and d) Cu-$PhCl_2$.

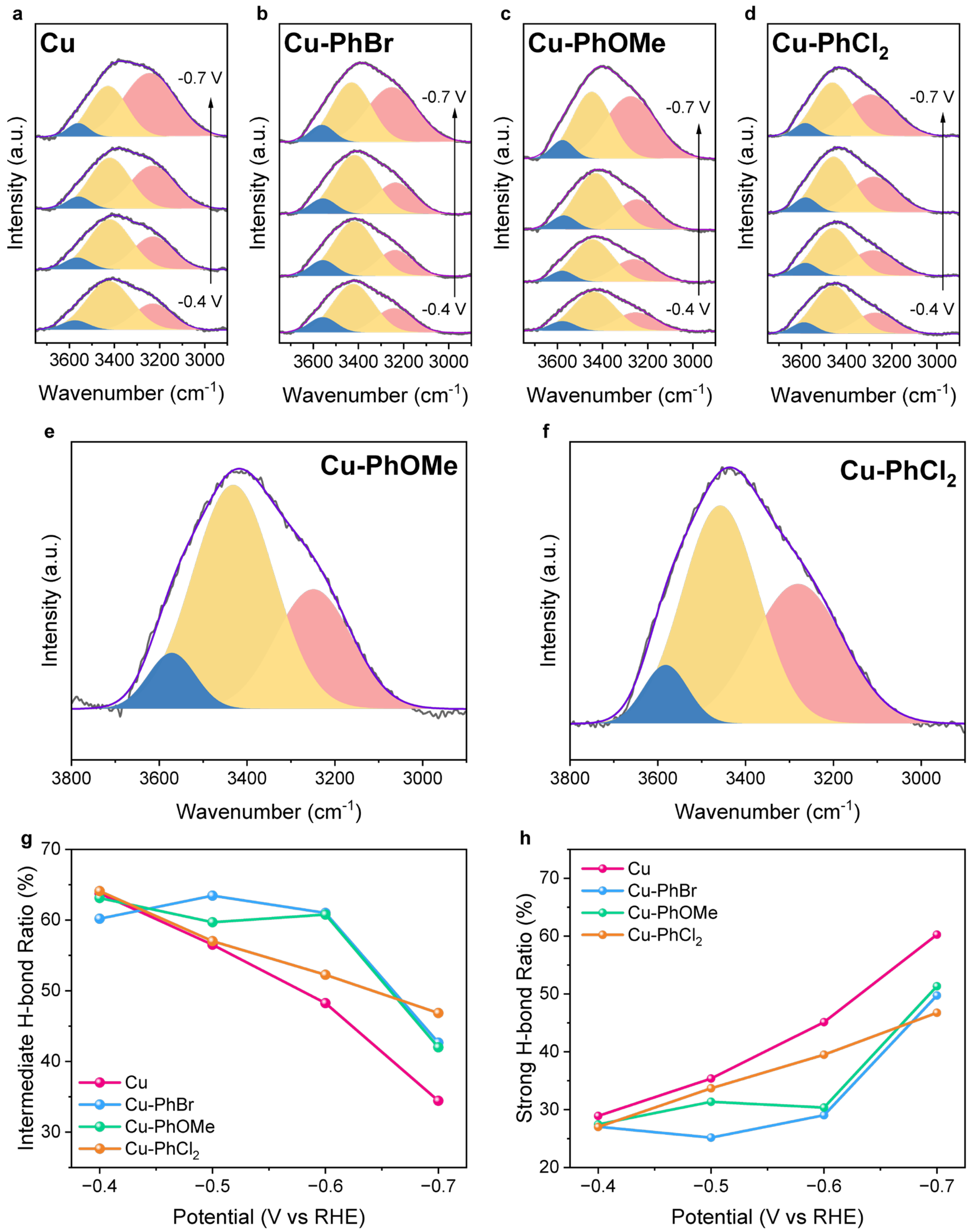

Figure S29. *Operando* ATR-SEIRAS spectra for v(O–H) of interfacial H_2O in CO_2-saturated 0.1 M $KHCO_3$ (pH = 6.8). Gaussian fit deconvoluted spectra for v(O–H) signal from -0.4 V_{RHE} to -0.7 V_{RHE} with a step of 0.1 V for a) Cu, b) Cu-PhBr, c) Cu-PhOMe and d) Cu- $PhCl_2$ with free or low H-bonded H_2O (blue), intermediate H-bonded H_2O (yellow), and strong H-bonded H_2O (red). Gaussian fit deconvoluted spectra from v_{OH} signal at -0.6 V_{RHE} for e) Cu-PhOMe and f) Cu- $PhCl_2$. Water structure ratio for g) intermediate and h) strong H-bonded water.

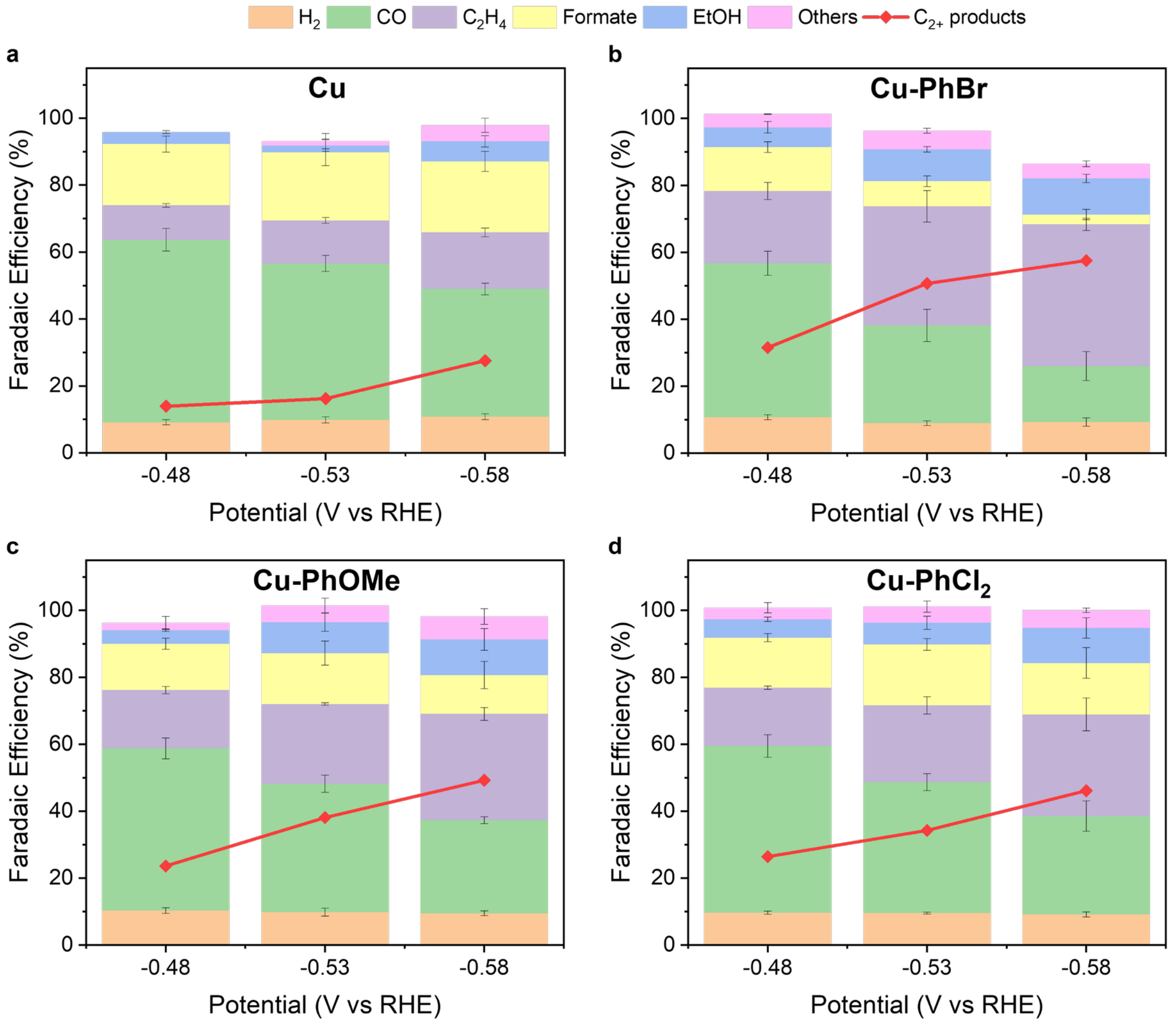

Figure S30. Selectivity of CO_2RR at different applied potentials. Faradaic efficiency of a) Cu, b) Cu-PhBr, c) Cu-PhOMe and d) Cu-$PhCl_2$ under different applied potentials in 1 M KOH. The experiment for each electrode was performed three times to obtain the mean and the standard deviation as the error bar.

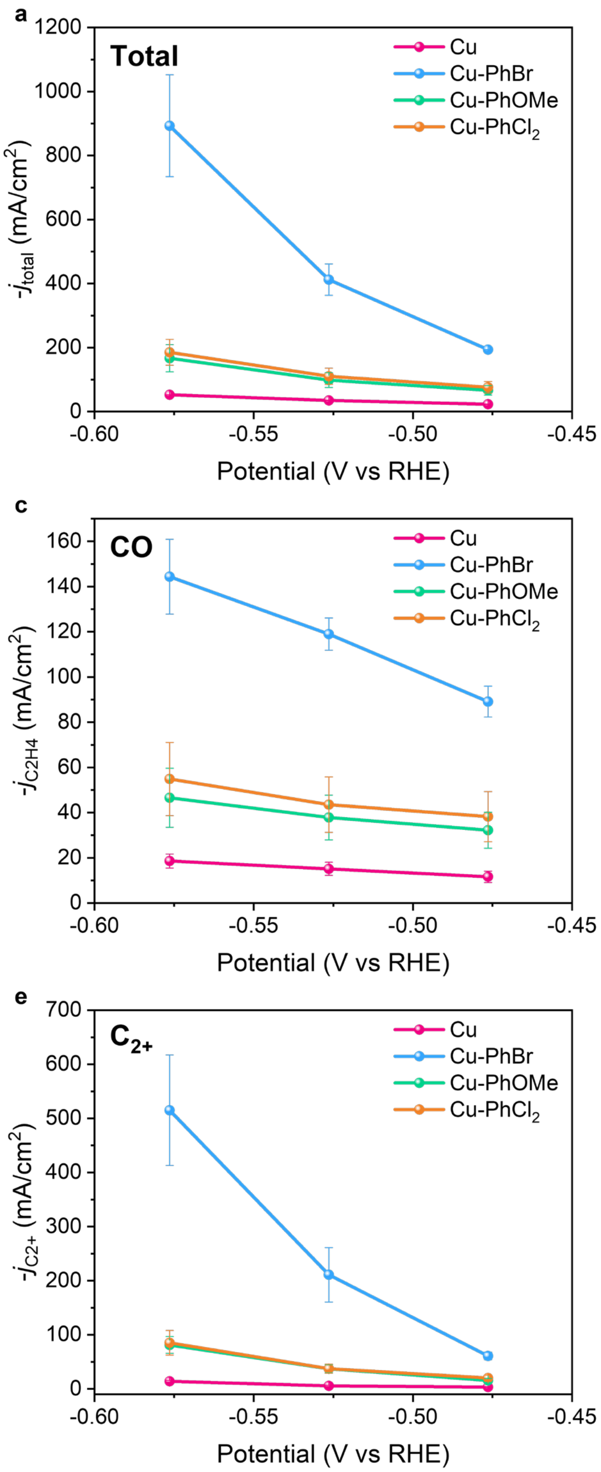

Figure S31. Activity of CO_2RR at different applied potentials. a) Total current density of Cu and Cu-PhR in 1 M KOH. Partial current densities of Cu and Cu-PhR for b) H_2, c) CO, d) C_2H_4 and e) C_{2+} under different potentials in 1 M KOH. The experiment for each electrode was performed three times to obtain the mean and the standard deviation as the error bar.

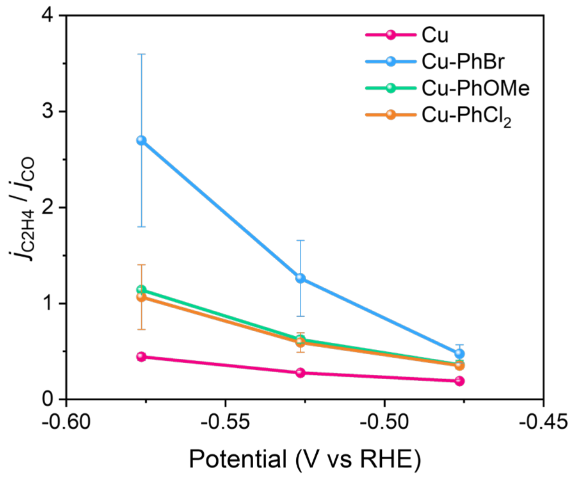

Figure S32. Ratio between j_{C2H4} and j_{CO} under different applied potential in 1 M KOH for unmodified and modified Cu. The experiment for each electrode was performed three times to obtain the mean and the standard deviation as the error bar.

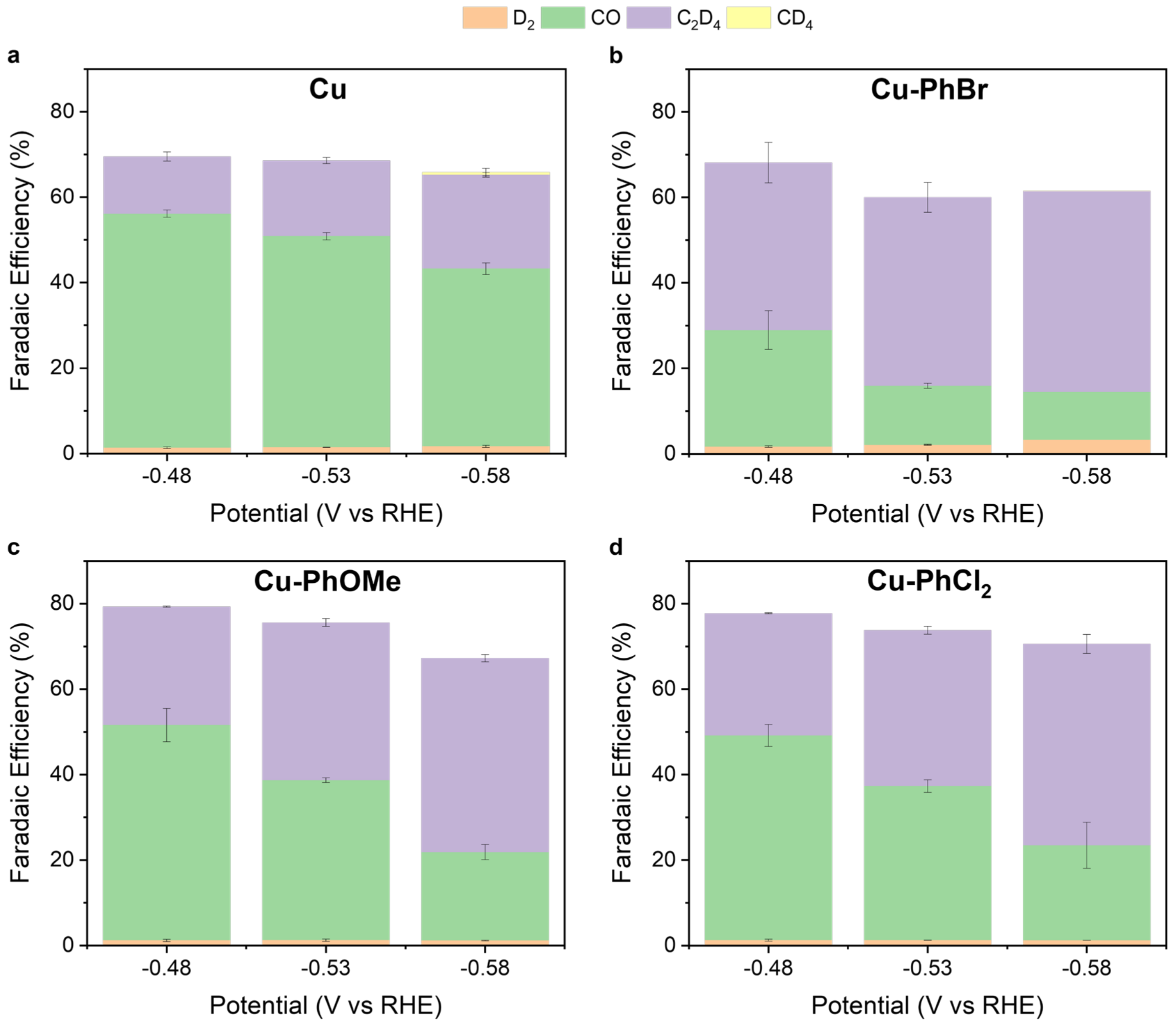

Figure S33. Selectivity of CO_2RR in 1 M KOH in D_2O at different applied potentials for KIE of hydrogen. Faradaic efficiency of a) Cu, b) Cu-PhBr, c) Cu-PhOMe and d) Cu-PhCl_2 under different applied potentials in 1 M KOH in D_2O. The experiment for each electrode was performed two times except for Cu-PhBr at -0.58 V_{RHE} to obtain the mean and the standard deviation as the error bar. These data were used to calculate the KIE of deuterium evolution reaction (DER) with CO_2 purged through the flow cell.

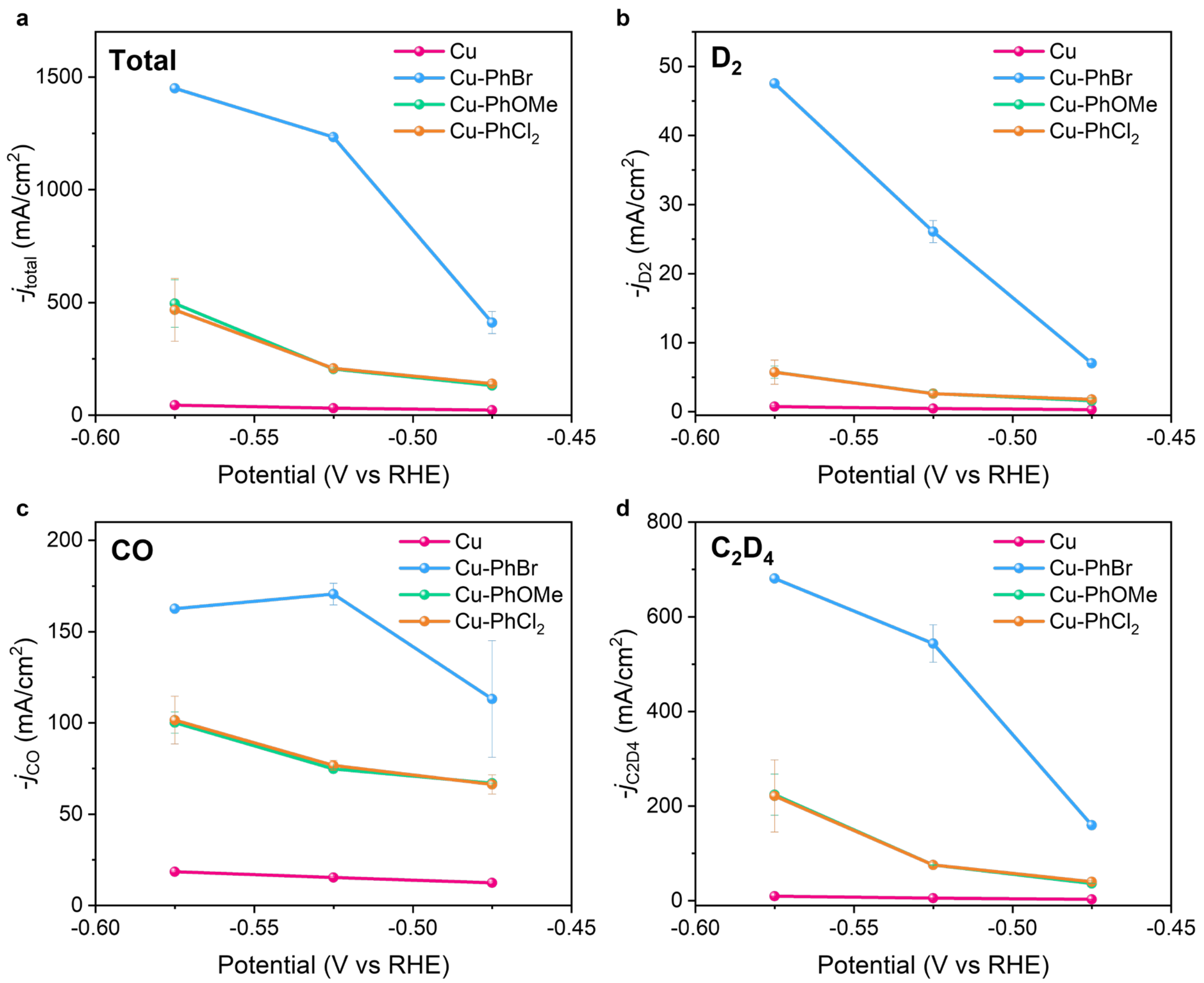

Figure S34. Activity of CO_2RR in 1 M KOH in D_2O at different applied potentials for KIE of hydrogen. a) Total current density of Cu and Cu-PhR in 1 M KOH D_2O. Partial current densities of Cu and Cu-PhR for b) D_2, c) CO and d) C_2D_4 under different potentials in 1 M KOH in D_2O. The experiment for each electrode was performed two times except for Cu-PhBr at -0.58 V_{RHE} to obtain the mean and the standard deviation as the error bar. These data were used to calculate the KIE of DER with CO_2 purged through the flow cell.

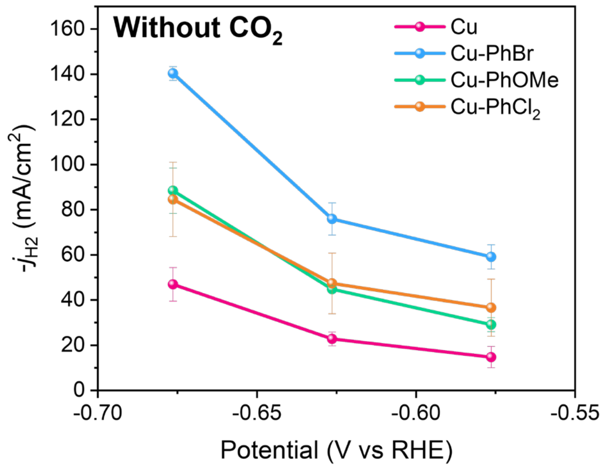

Figure S35. Activity of HER in 1 M KOH at different applied potentials. HER activity measured in N_2 (dashed) purged condition.

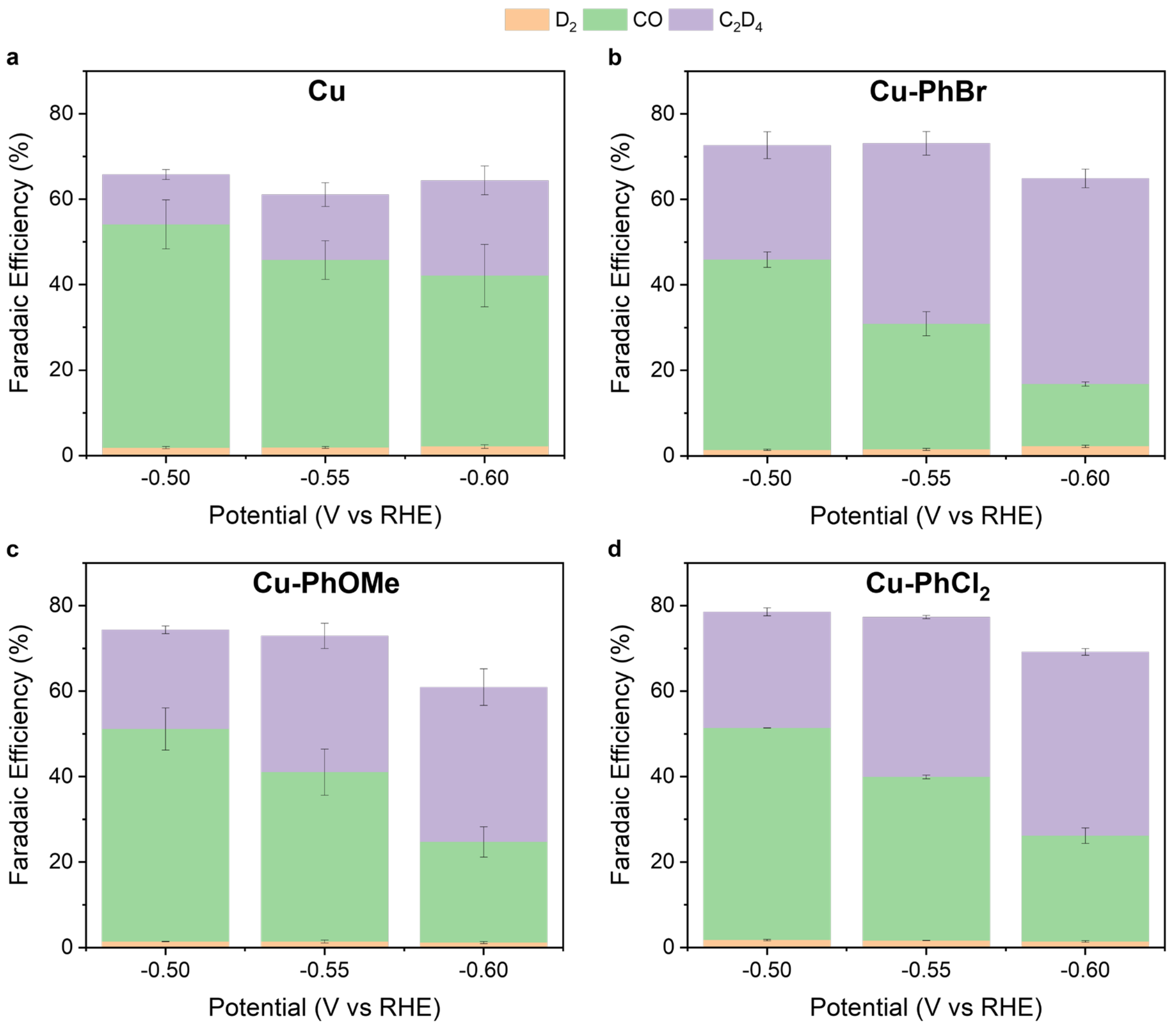

Figure S36. Selectivity of CO_2RR in 1 M KOH in D_2O at different applied potentials for KIE of ethylene. Faradaic efficiency of a) Cu, b) Cu-PhBr, c) Cu-PhOMe and d) Cu-$PhCl_2$ under different applied potentials in 1 M KOH in D_2O. The experiment for each electrode was performed two times to obtain the mean and the standard deviation as the error bar. These data were used to calculate the KIE of CO_2RR to ethylene.

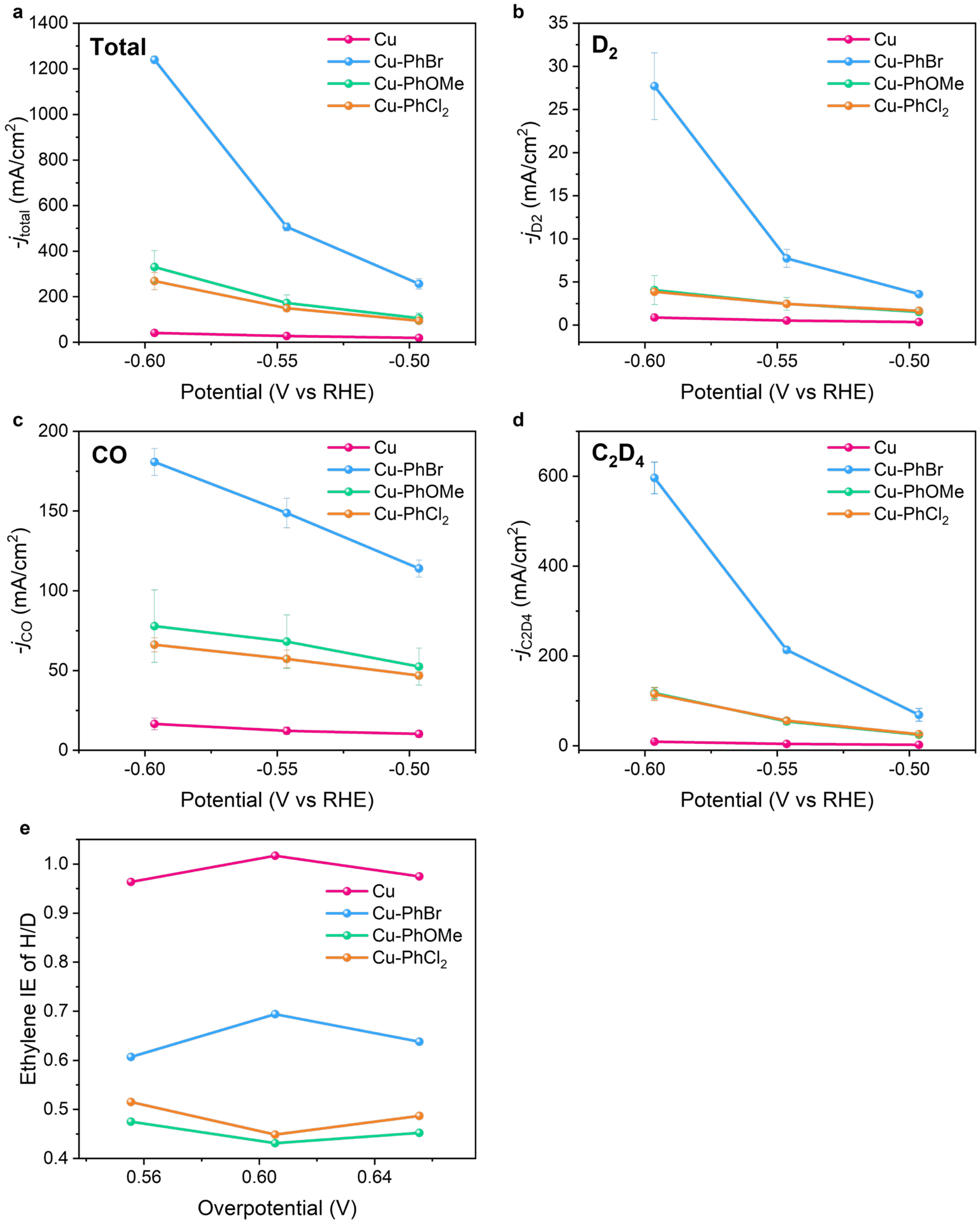

Figure S37. Activity of CO_2RR in 1 M KOH in D_2O at different applied potentials for KIE of ethylene. a) Total current density of Cu and Cu-PhR in 1 M KOH. Partial current densities of Cu and Cu-PhR for b) D_2, c) CO and d) C_2D_4 under different potentials in 1 M KOH in D_2O. e) Calculated ethylene kinetic isotope effect (KIE) of H/D at different applied overpotential. The experiment for each electrode was performed two times to obtain the mean and the standard deviation as the error bar. These data were used to calculate the KIE of CO_2RR to ethylene.

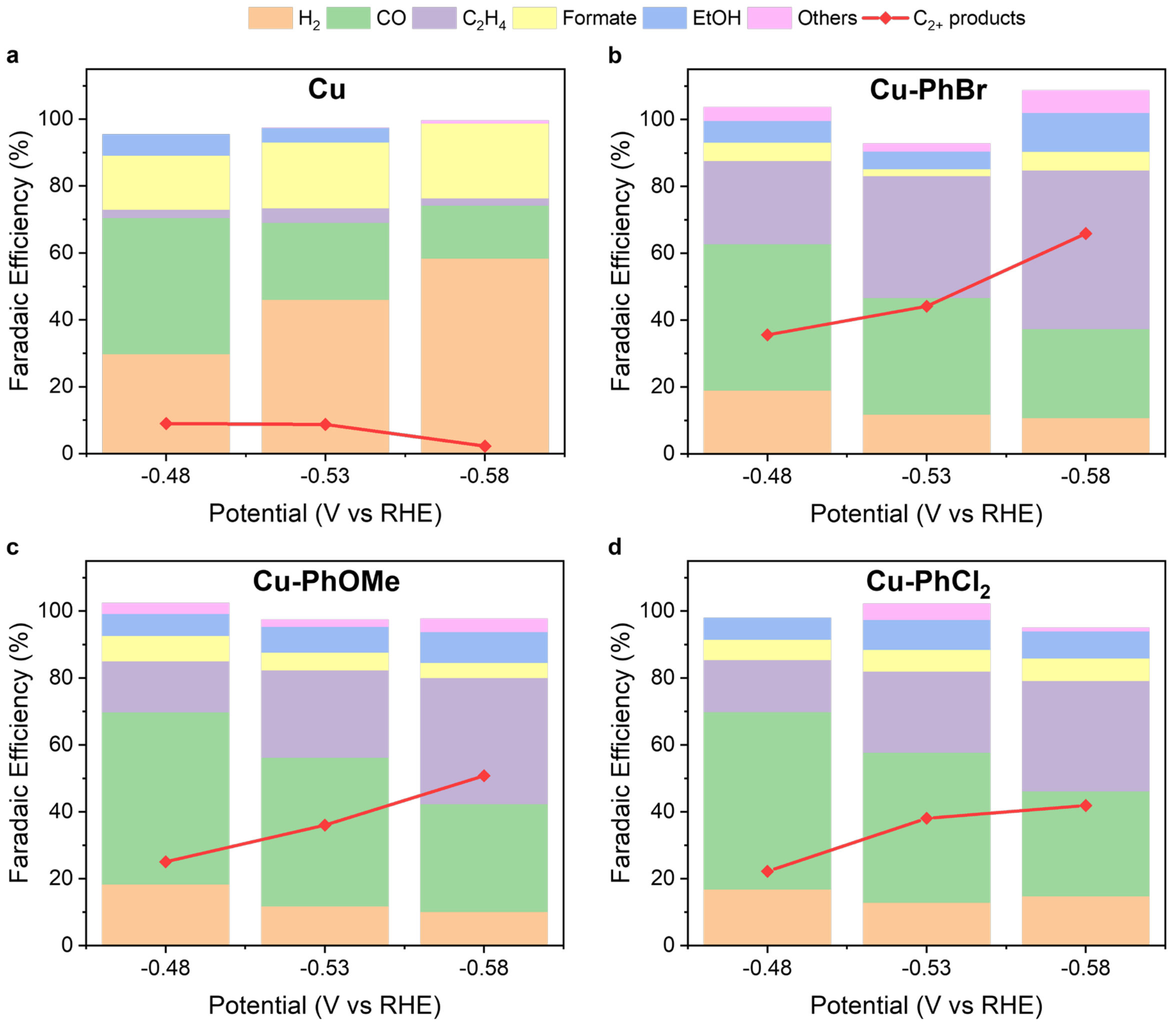

Figure S38. Selectivity of CO_2RR in 1 M NaOH at different applied potentials. Faradaic efficiency of a) Cu, b) Cu-PhBr, c) Cu-PhOMe and d) Cu-$PhCl_2$ under different applied potentials in 1 M NaOH.

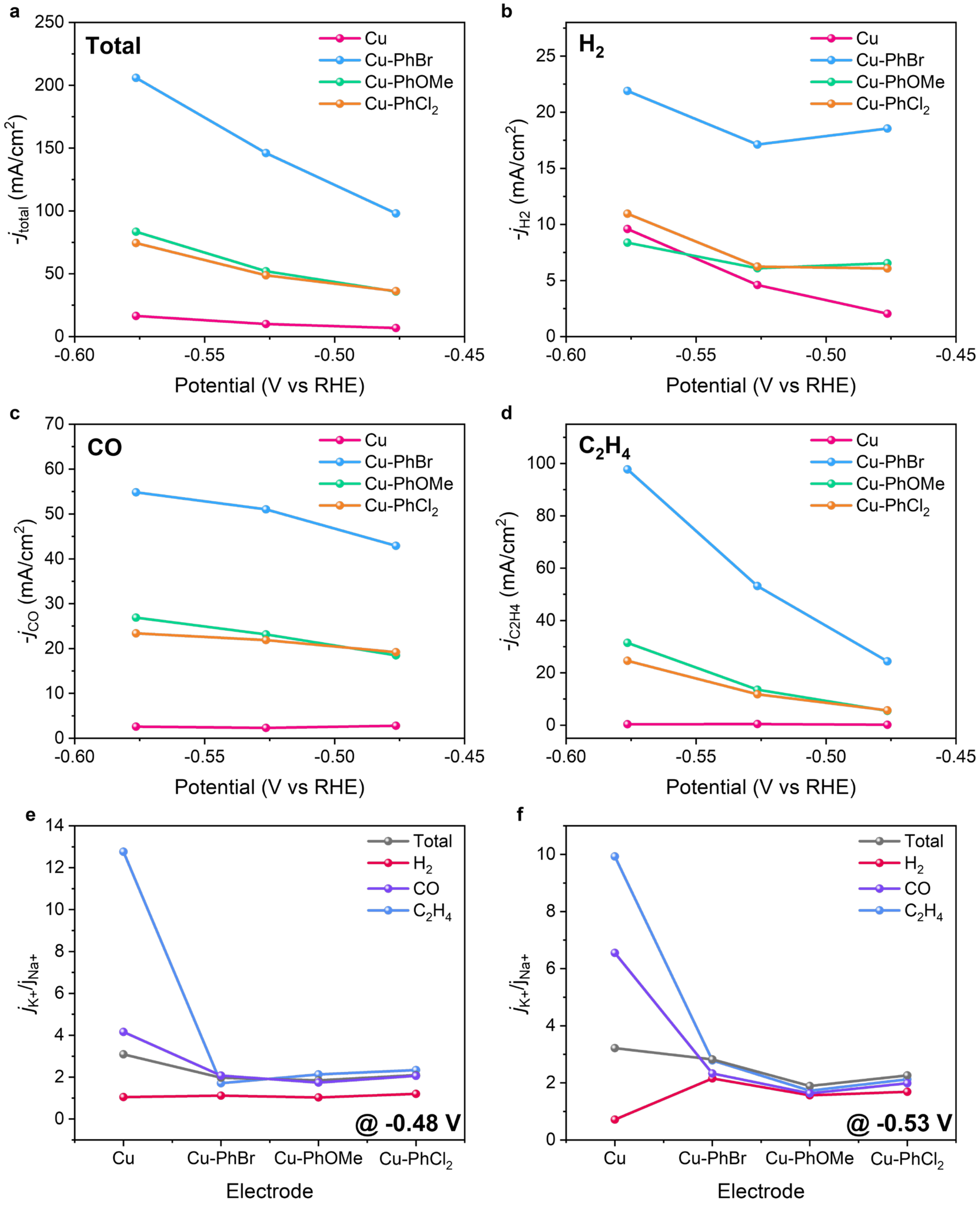

Figure S39. **Activity of CO_2RR in 1 M NaOH at different applied potentials.** Total current density of Cu and Cu-PhR in 1 M NaOH. Partial current densities of Cu and Cu-PhR for b) H_2, c) CO and d) C_2H_4 under different potentials in 1 M NaOH. Partial current density in 1 M KOH divided by that in 1 M NaOH for Cu and Cu-PhR at e) -0.48 V_{RHE} and f) -0.53 V_{RHE}.

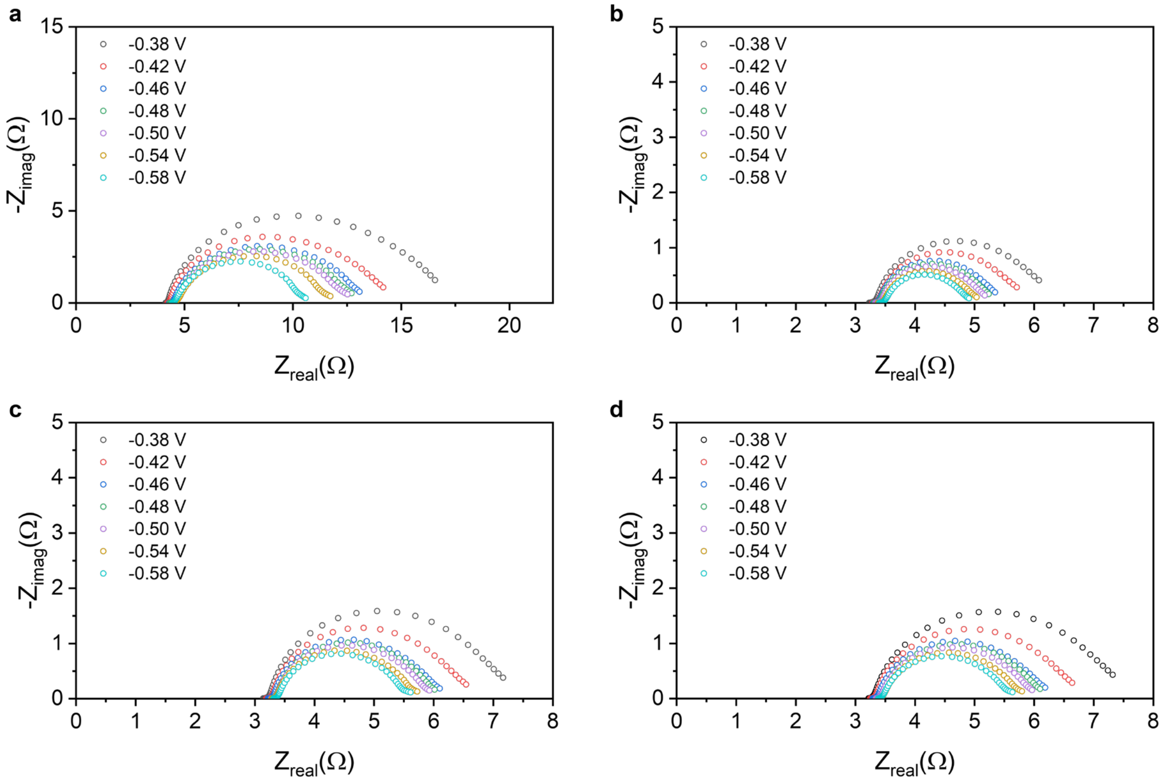

Figure S40. Raw Nyquist plots for EIS measurement. Raw data for Nyquist plots for EIS under different applied potentials for a) Cu, b) Cu-PhBr, c) Cu-PhOMe and d) Cu-$PhCl_2$.

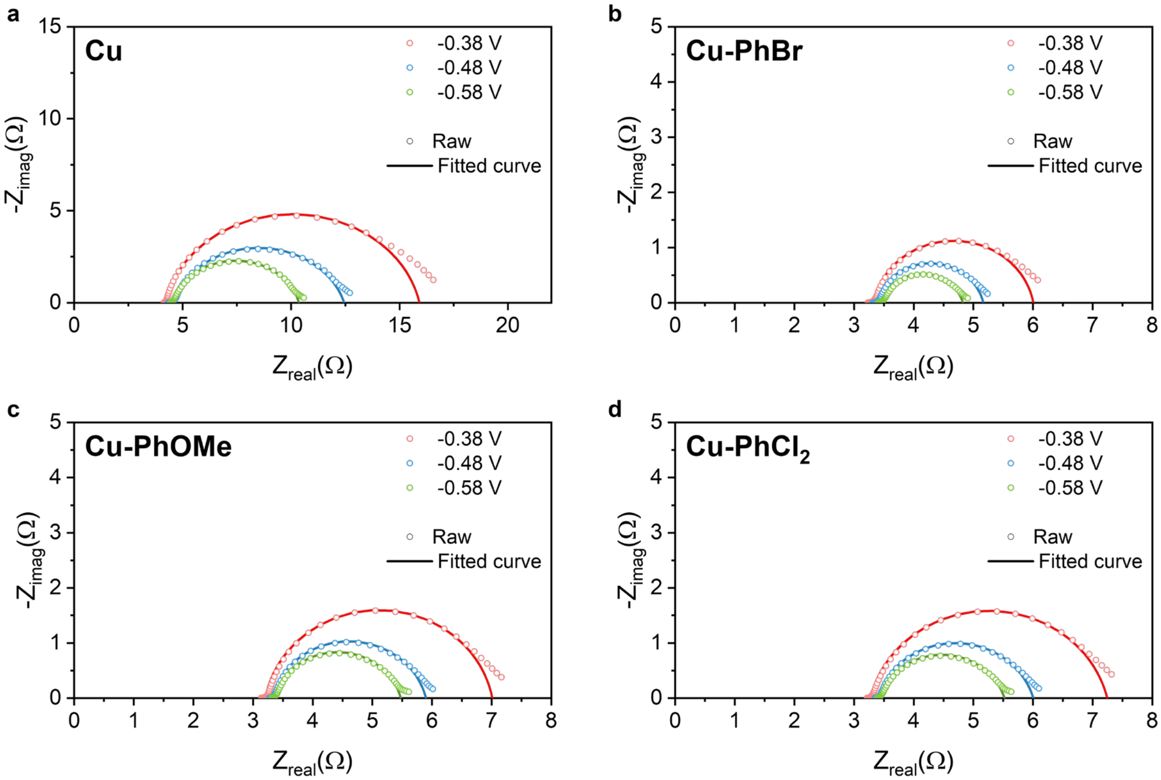

Figure S41. Fitted Nyquist plots for EIS measurement. Fitted data (dashed line) and raw data (circles) for Nyquist plots for EIS under different applied potentials for a) Cu, b) Cu-PhBr, c) Cu-PhOMe and d) Cu-$PhCl_2$. The model in the inset of Figure 4d was used to fit the EIS data.

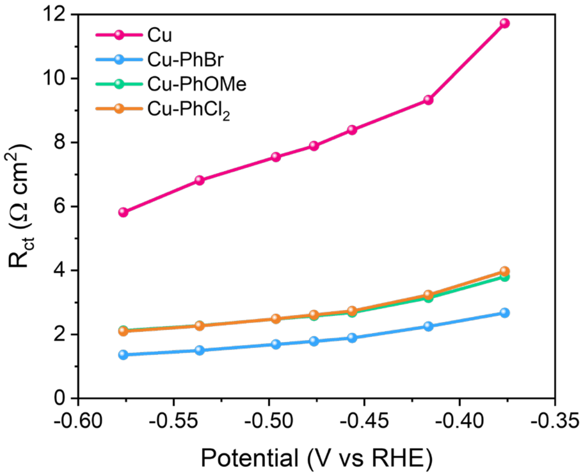

Figure S42. Charge transfer resistance (R_{ct}) of Cu and Cu-PhR. R_{ct} under different applied potentials, extracted from fitting of Nyquist plot in Supplementary Figure 38. The model in Figure 5d was used to fit the EIS data.

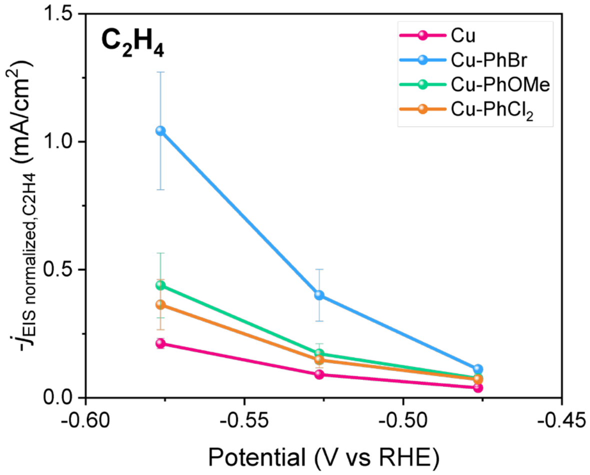

Figure S43. $\boldsymbol{j_{C2H4}}$ **normalized by surface area obtained from the pseudocapacitance.** The values of pseudocapacitance were obtained from Fig. 4d.

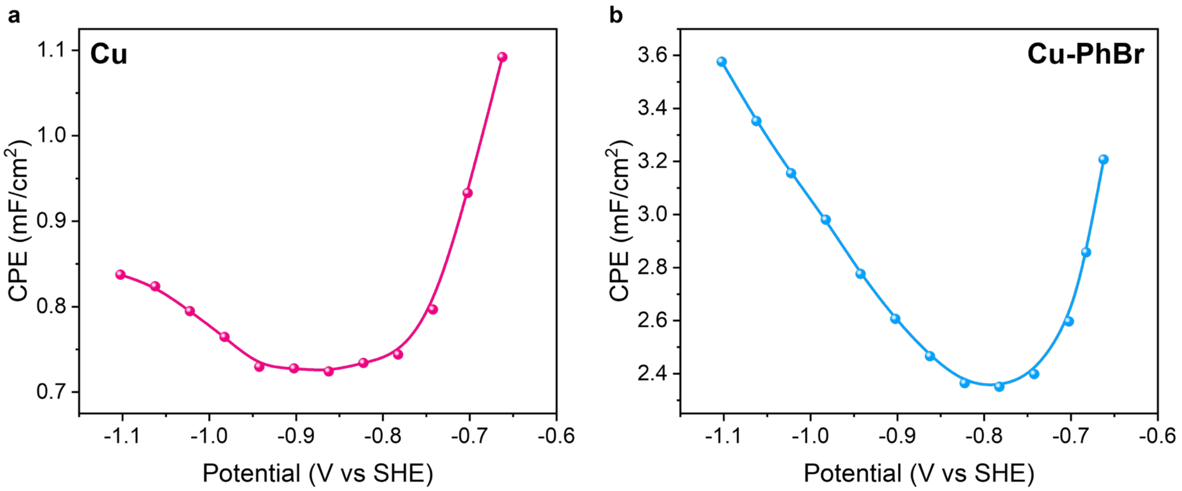

Figure S44. Potential of zero charge (PZC). PZC measured with EIS at different applied potentials in 0.1 M K_2SO_4 for a) bare Cu and b) Cu-PhBr.

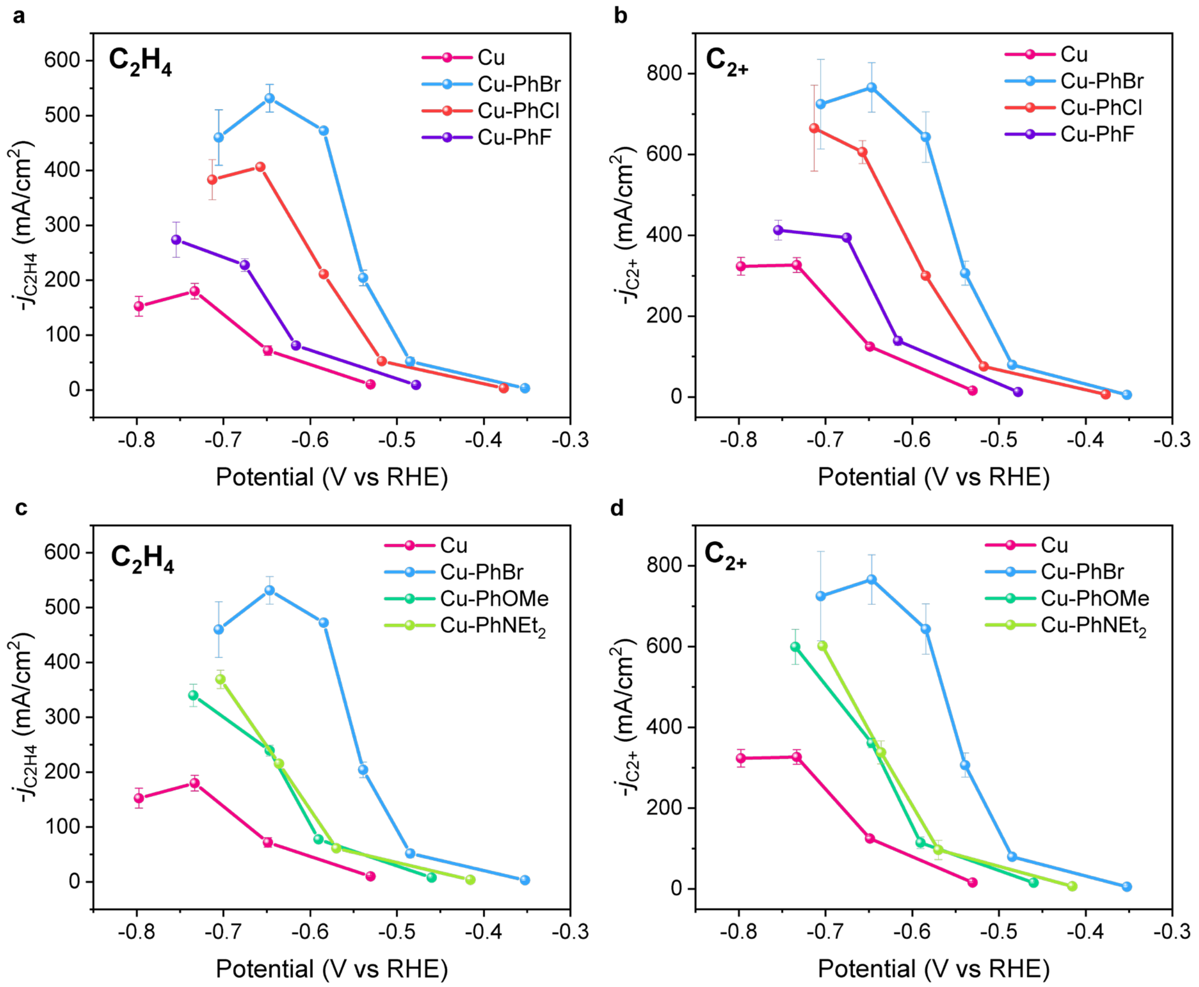

Figure S45. Comparison of electrochemical performance among Cu-PhR in 1 M KOH using a flow cell setup. Partial current densities of a) C_2H_4 and b) C_{2+} products of electrodes with halogenated aryl groups compared to Cu and Cu-PhBr. Partial current densities of c) C_2H_4 and d) C_{2+} products of H-bond acceptor compared to Cu and Cu-PhBr. Except Cu and Cu-PhBr, all the experiments were carried out two times and the results show the mean value with an error bar of standard deviation.

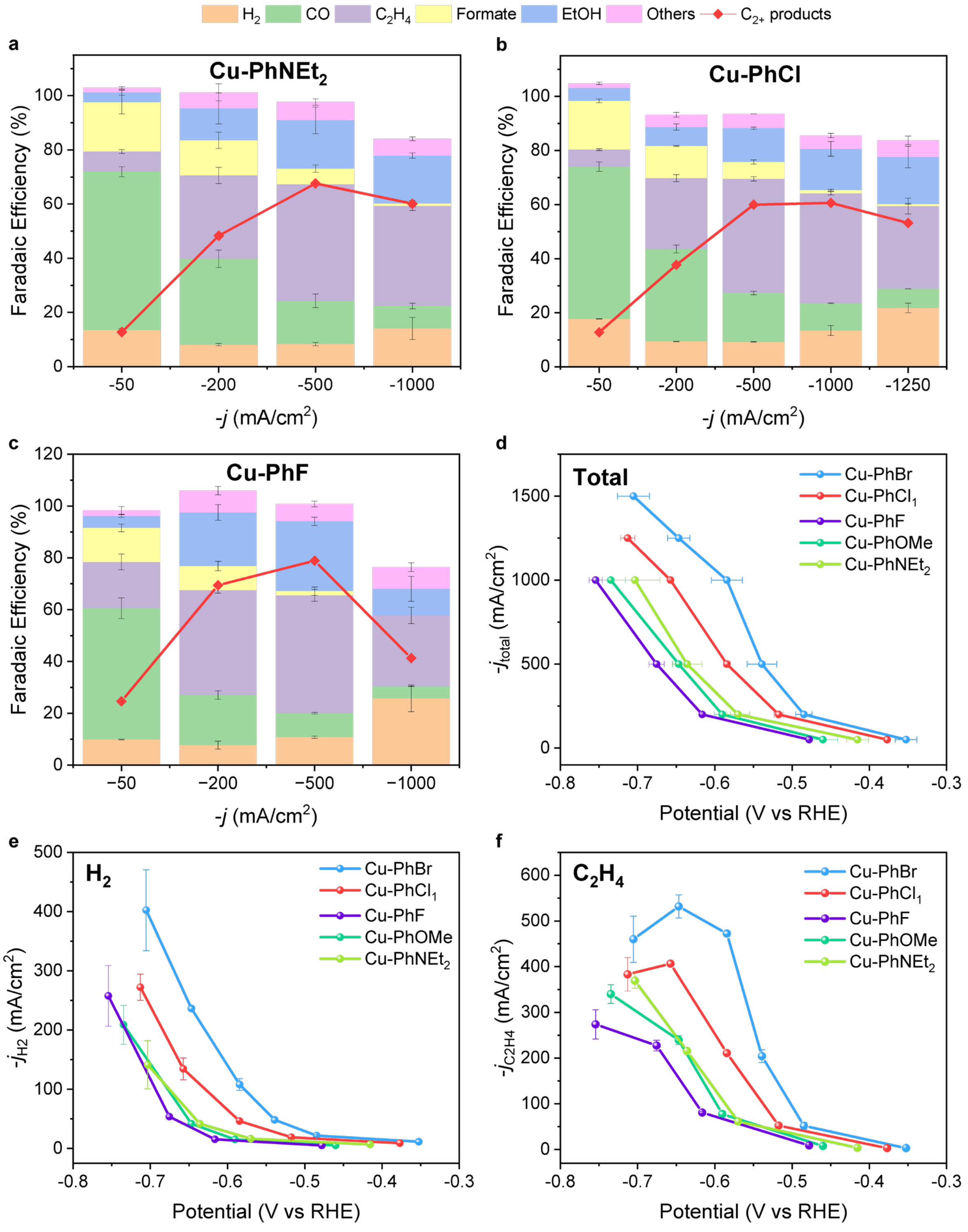

Figure S46. CO_2RR performance of electrodes with monosubstituted aryl layer. Faradaic efficiency of a) Cu-PhNEt$_2$ b) Cu-PhCl and c) Cu-PhF in 1 M KOH. d) Total current density and partial current densities of electrodes with monosubstituted aryl layer for e) H_2 and f) C_2H_4 and in 1 M KOH. The experiments were carried out three times for Cu-PhBr and Cu-PhOMe; and two times for Cu-PhNEt$_2$, Cu-PhCl and Cu-PhF. The results show the mean value with an error bar of standard deviation.

Table S1. Summary of recently reported catalytic performance of Cu-based electrochemical CO_2 reduction to C_{2+} products.

Electrode	Electrocatalytic cell	FE (%)		*j* (mA cm^{-2})		Electrolyte	Ref
		C_2H_4	C_{2+}	C_2H_4	C_{2+}		
Cu-PhBr (present work)	Flow cell	43	61	-532	-766	1 M KOH	-
Cu NN/ionomer	MEA	80	82	-545	-560	0.5 M $KHCO_3$	[12]
F-Cu	Flow cell	55	70	-960	-1120	1 M KOH	[13]
Cu-Molecule 12	Flow cell	72	86	-232	-276	1 M $KHCO_3$	[14]
T-CuO	Flow cell	68	78	-540	-620	1 M KOH	[15]
TA-Cu	Flow cell	64	-	-316	-	1 M KOH	[16]
Cu-P1	Flow cell	72	87	-312	-377	1 M KOH	[17]
MgAl-LDH/Cu	Flow cell	55	69	-165	-207	1 M $KHCO_3$	[18]
Cu@AIL	Flow cell	35	81	-313	-732	1 M KOH	[19]
N-Cu	Flow cell	-	73.7	-	-909	1 M KOH	[20]

Table S2. Double layer capacitance (C_{dl}), surface roughness factor (R_f) and electrochemically active surface areas (ECSAs) for Cu and Cu-PhR obtained from Figure S17 and S18. The experiment for each electrode was performed three times to obtain the mean and the standard deviation as the error bar.

Entry	C_{dl} (mF cm^{-2})	R_f	ECSA (cm^2)
Cu	13.07 ± 0.84	451	451
Cu-PhBr	15.37 ± 1.86	530	530
Cu-PhOMe	12.45 ± 0.69	429	429
Cu-$PhCl_2$	13.91 ± 0.71	480	480

R_f was calculated from the ratio between the C_{dl} of the corresponding catalyst and that of smooth polycrystalline Cu electrode, namely 29 µF cm^{-2}. ECSA is then obtained by multiplying R_f with the geometric electrode surface area (1 cm^2).

Table S3. Contact angle between the electrode and water droplet for Cu and Cu-PhR. The experiment for each electrode was performed three times to obtain the mean and the standard deviation as the error bar.

Entry	1 (°)	2 (°)	3 (°)	Average (°)
Cu	133	130	128	130 ± 2.5
Cu-PhBr	142	141	143	142 ± 1.0
Cu-PhOMe	131	131	129	130 ± 1.2
Cu-$PhCl_2$	146	140	144	143 ± 3.1

Table S4. Ratio between j_{CO2RR} and j_{H2} under different applied potential in 1 M KOH for unmodified and modified Cu.

Potential	Cu	Cu-PhBr	Cu-PhOMe	Cu-$PhCl_2$
–0.48 V_{RHE}	9.52	8.44	8.36	9.42
–0.53 V_{RHE}	8.48	9.79	9.31	9.56
–0.58 V_{RHE}	8.05	8.29	9.36	9.95

REFERENCES

(1) Rodríguez-Fernández, L.; Albarrán-Velo, J.; Lavandera, I.; Gotor-Fernández, V. From Diazonium Salts to Optically Active 1-Arylpropan-2-Ols Through a Sequential Photobiocatalytic Approach. *Adv. Synth. Catal.* **2023**, *365* (11), 1883–1892. https://doi.org/10.1002/adsc.202300245.

(2) Mauritz, K. A.; Moore, R. B. State of Understanding of Nafion. *Chem. Rev.* **2004**, *104* (10), 4535–4586. https://doi.org/10.1021/cr0207123.

(3) Lu, X.; Zhao, C. Electrodeposition of Hierarchically Structured Three-Dimensional Nickel–Iron Electrodes for Efficient Oxygen Evolution at High Current Densities. *Nat. Commun.* **2015**, *6* (1), 6616. https://doi.org/10.1038/ncomms7616.

(4) Pamidimukkala, K. M.; Rogers, D.; Skinner, G. B. Ideal Gas Thermodynamic Properties of CH3, CD3, CD4, C2D2, C2D4, C2D6, C2H6, CH3N2CH3, and CD3N2CD3. *J. Phys. Chem. Ref. Data* **1982**, *11* (1), 83–99. https://doi.org/10.1063/1.555656.

(5) Rossini, F. D.; Knowlton, J. W.; Johnston, H. L. Heat and Free Energy of Formation of Deuterium Oxide. *J. Res. Natl. Bur. Stand.* **1940**, *24* (4), 369. https://doi.org/10.6028/jres.024.020.

(6) Yoo, S.; Yoo, S.; Deng, G.; Sun, F.; Lee, K.; Jang, H.; Lee, C. W.; Liu, X.; Jang, J.; Tang, Q.; Hwang, Y. J.; Hyeon, T.; Bootharaju, M. S. Nanocluster Surface Microenvironment Modulates Electrocatalytic CO2 Reduction. *Adv. Mater.* **2024**, *36* (13), 2313032. https://doi.org/10.1002/adma.202313032.

(7) Zhang, D.; Virchenko, V.; Jansen, C.; Bakker, J. M.; Meyer, J.; Kleyn, A. W.; Groot, I. M. N.; Berg, O. T.; Juurlink, L. B. F. Characterization of CO Adsorbed to Clean and Partially Oxidized Cu(211) and Cu(111). *J. Phys. Chem. C* **2023**, *127* (50), 24158–24167. https://doi.org/10.1021/acs.jpcc.3c05954.

(8) Hensley, A. J. R.; Therrien, A. J.; Zhang, R.; Marcinkowski, M. D.; Lucci, F. R.; Sykes, E. C. H.; McEwen, J.-S. CO Adsorption on the "29" CuxO/Cu(111) Surface: An Integrated DFT, STM, and TPD Study. *J. Phys. Chem. C* **2016**, *120* (44), 25387–25394. https://doi.org/10.1021/acs.jpcc.6b07670.

(9) Ma, W.; Xie, S.; Zhang, B.; He, X.; Liu, X.; Mei, B.; Sun, F.; Jiang, Z.; Lin, L.; Zhang, Q.; Ren, B.; Fu, G.; Hu, X.; Wang, Y. Copper Lattice Tension Boosts Full-Cell CO Electrolysis to Multi-Carbon Olefins and Oxygenates. *Chem* **2023**, *9* (8), 2161–2177. https://doi.org/10.1016/j.chempr.2023.03.022.

(10) Chen, D.; Wang, Y.; Liu, D.; Liu, H.; Qian, C.; He, H.; Yang, J. Surface Composition Dominates the Electrocatalytic Reduction of CO2 on Ultrafine CuPd Nanoalloys. *Carbon Energy* **2020**, *2* (3), 443–451. https://doi.org/10.1002/cey2.38.

(11) Celante, V. G.; Freitas, M. B. J. G. Electrodeposition of Copper from Spent Li-Ion Batteries by Electrochemical Quartz Crystal Microbalance and Impedance Spectroscopy Techniques. *J. Appl. Electrochem.* **2010**, *40* (2), 233–239. https://doi.org/10.1007/s10800-009-9996-x.

(12) Wu, H.; Huang, L.; Timoshenko, J.; Qi, K.; Wang, W.; Liu, J.; Zhang, Y.; Yang, S.; Petit, E.; Flaud, V.; Li, J.; Salameh, C.; Miele, P.; Lajaunie, L.; Roldán Cuenya, B.; Rao, D.; Voiry, D. Selective and Energy-Efficient Electrosynthesis of Ethylene from CO2 by Tuning the Valence of Cu Catalysts through Aryl Diazonium Functionalization. *Nat. Energy* **2024**, *9* (4), 422–433. https://doi.org/10.1038/s41560-024-01461-6.

(13) Ma, W.; Xie, S.; Liu, T.; Fan, Q.; Ye, J.; Sun, F.; Jiang, Z.; Zhang, Q.; Cheng, J.; Wang, Y. Electrocatalytic Reduction of CO2 to Ethylene and Ethanol through Hydrogen-Assisted C–C Coupling over Fluorine-Modified Copper. *Nat. Catal.* **2020**, *3* (6), 478–487. https://doi.org/10.1038/s41929-020-0450-0.

(14) Li, F.; Thevenon, A.; Rosas-Hernández, A.; Wang, Z.; Li, Y.; Gabardo, C. M.; Ozden, A.; Dinh, C. T.; Li, J.; Wang, Y.; Edwards, J. P.; Xu, Y.; McCallum, C.; Tao, L.; Liang, Z.-Q.; Luo, M.; Wang, X.; Li, H.; O'Brien, C. P.; Tan, C.-S.; Nam, D.-H.; Quintero-Bermudez, R.; Zhuang, T.-T.; Li, Y. C.; Han, Z.; Britt, R. D.; Sinton, D.; Agapie, T.; Peters, J. C.; Sargent, E. H. Molecular Tuning of CO2-to-Ethylene Conversion. *Nature* **2020**, *577* (7791), 509–513. https://doi.org/10.1038/s41586-019-1782-2.

(15) Liu, Z.; Lv, X.; Kong, S.; Liu, M.; Liu, K.; Zhang, J.; Wu, B.; Zhang, Q.; Tang, Y.; Qian, L.; Zhang, L.; Zheng, G. Interfacial Water Tuning by Intermolecular Spacing for Stable CO2 Electroreduction to C2+ Products. *Angew. Chem. Int. Ed.* **2023**, *62* (43), e202309319. https://doi.org/10.1002/anie.202309319.

(16) Chen, S.; Ye, C.; Wang, Z.; Li, P.; Jiang, W.; Zhuang, Z.; Zhu, J.; Zheng, X.; Zaman, S.; Ou, H.; Lv, L.; Tan, L.; Su, Y.; Ouyang, J.; Wang, D. Selective CO2 Reduction to Ethylene Mediated by Adaptive Small-Molecule Engineering of Copper-Based Electrocatalysts. *Angew. Chem. Int. Ed.* **2023**, *62* (50), e202315621. https://doi.org/10.1002/anie.202315621.

(17) Chen, X.; Chen, J.; Alghoraibi, N. M.; Henckel, D. A.; Zhang, R.; Nwabara, U. O.; Madsen, K. E.; Kenis, P. J. A.; Zimmerman, S. C.; Gewirth, A. A. Electrochemical CO2-to-Ethylene Conversion on Polyamine-Incorporated Cu Electrodes. *Nat. Catal.* **2021**, *4* (1), 20–27. https://doi.org/10.1038/s41929-020-00547-0.

(18) Xu, Y. N.; Li, W.; Fu, H. Q.; Zhang, X. Y.; Zhao, J. Y.; Wu, X.; Yuan, H. Y.; Zhu, M.; Dai, S.; Liu, P. F.; Yang, H. G. Tuning the Microenvironment in Monolayer MgAl Layered Double Hydroxide for CO2-to-Ethylene Electrocatalysis in Neutral Media. *Angew. Chem. Int. Ed.* **2023**, *62* (19), e202217296. https://doi.org/10.1002/anie.202217296.
(19) Tan, Z.; Zhang, J.; Yang, Y.; Zhong, J.; Zhao, Y.; Hu, J.; Han, B.; Chen, Z. Alkaline Ionic Liquid Microphase Promotes Deep Reduction of CO2 on Copper. *J. Am. Chem. Soc.* **2023**, *145* (40), 21983–21990. https://doi.org/10.1021/jacs.3c06860.
(20) Zheng, M.; Wang, P.; Zhi, X.; Yang, K.; Jiao, Y.; Duan, J.; Zheng, Y.; Qiao, S.-Z. Electrocatalytic CO2-to-C2+ with Ampere-Level Current on Heteroatom-Engineered Copper via Tuning *CO Intermediate Coverage. *J. Am. Chem. Soc.* **2022**, *144* (32), 14936–14944. https://doi.org/10.1021/jacs.2c06820.
